# Two parallel lineage-committed progenitors contribute to the developing brain

**DOI:** 10.1101/2025.07.02.662771

**Published:** 2025-07-05

**Authors:** Carolyn E. Dundes, Rayyan T. Jokhai, Hadia Ahsan, Rachel S. Kang, Rachel E.A. Salomon-Shulman, Arjun Rajan, Yoon Seok Kim, Liam J. Stanton, Christine Xu, Stephanie Do, Brennan D. McDonald, José Miguel Andrade López, Hugo A. Urrutia, Hannah Greenfeld, Alicia Wong, Yimiao Qu, Andrew S. Petkovic, Yi Miao, K. Christopher Garcia, Michelle Monje, Daniel E. Wagner, Marianne E. Bronner, Christopher J. Lowe, Kyle M. Loh

**Affiliations:** 1 Department of Developmental Biology, Stanford University, Stanford, CA USA; 2 Institute for Stem Cell Biology & Regenerative Medicine, Stanford University, Stanford, CA USA; 3 Department of Neurology and Neurological Sciences, Howard Hughes Medical Institute, Stanford University, Stanford, CA USA; 4 Hopkins Marine Station, Department of Biology, Stanford University, Pacific Grove, CA USA; 5 Division of Biology and Biological Engineering, California Institute of Technology, Pasadena, CA USA; 6 Department of Obstetrics, Gynecology and Reproductive Science, Center for Reproductive Sciences, Eli and Edythe Broad Center for Regeneration Medicine and Stem Cell Research, University of California San Francisco, San Francisco, CA, USA; 7 Department of Molecular and Cellular Physiology, Department of Structural Biology, Howard Hughes Medical Institute, Stanford University, Stanford, CA, USA

## Abstract

The hindbrain is a life-sustaining brain region. In one model, a common neural progenitor generates all brain regions. Here our studies of mouse embryos and human pluripotent stem cells (hPSCs) support a different model: two parallel brain progenitors emerge simultaneously during gastrulation, anterior neural ectoderm (forebrain/midbrain progenitor) and posterior neural ectoderm (hindbrain progenitor). Not only are they lineage-committed to respectively form forebrain/midbrain vs. hindbrain *in vitro*, but they also have diverging chromatin landscapes foreshadowing future forebrain/midbrain vs. hindbrain identities. Leveraging these differences, we differentiated hPSCs into hindbrain rhombomere 5/6-specific motor neurons, hitherto difficult to generate *in vitro*. We postulate the brain is a composite organ emanating from two lineage-restricted progenitors; these dual progenitors may be evolutionarily conserved across 550 million years from hemichordates to mammals.

## Introduction

Stem cell research strives to create diverse types of human brain cell. Given plentiful successes in converting human pluripotent stem cells (hPSCs) into forebrain and midbrain cells^1–3^, here we focus on generating cells of the human hindbrain, a life-sustaining brain region^4–6^. Whereas the forebrain executes higher-level cognitive processes, the hindbrain constitutes most of the brainstem and coordinates life-sustaining processes such as breathing, eating, sleep, and wakefulness^4–6^. Consequently, hindbrain injury and diffuse intrinsic pontine glioma (a childhood hindbrain cancer) are both deadly, as they impair consciousness, sensation, reflexes, and breathing^7,8^. Additionally, hindbrain motor neurons project through the cranial nerves to innervate face and neck muscles, and degeneration of hindbrain motor neurons likely compromises eating and swallowing in diseases such as spinal muscular atrophy (SMA) and amyotrophic lateral sclerosis (ALS), leading to choking, aspiration, pneumonia, and in some cases, death^9–11^. However, differentiation of hPSCs into specific brain cells that correspond to different brain regions—such as the forebrain, midbrain, and hindbrain—is predicated on a developmental roadmap of when and how brain progenitors diversify from one another^4–6,12–15^.

During gastrulation, pluripotent cells form the ectoderm germ layer in the 7-day-old (~E7.0) mouse embryo^16^. Subsequently, ectoderm forms neural, border, and surface ectoderm by E7.5, which will respectively create the brain, neural crest, and epidermis^16,17^. Arising during gastrulation, neural ectoderm represents the earliest brain-restricted progenitor^13,16^ and it subsequently differentiates into the forebrain, midbrain, and hindbrain by ~E8.5^18,19^.

An open question is whether, even during gastrulation, neural ectoderm cells are diverse and are already committed to generate different brain regions. In 1952, Nieuwkoop^20,21^ proposed that neural ectoderm harbors the broad potential to generate the forebrain, midbrain, and hindbrain^22–25^. Indeed, the forebrain, midbrain, and hindbrain are anatomically contiguous, consistent with a scenario wherein they originate from a common brain precursor. Certain methods to differentiate hPSCs into brain cell-types implicitly assume the existence of a common neural progenitor that can generate the forebrain, midbrain, and hindbrain^22,23^. By contrast, classical fate maps of zebrafish^26^, frog^27^, chicken^28^, and mouse^29,30^ embryos have instead suggested that during gastrulation, neural ectoderm cells are already *fated* to give rise to distinct parts of the brain. However, these fate maps could not exclude the common neural progenitor model, because they generally focused on the fates of neural ectoderm cells if left in their native signaling environment in the gastrulating embryo^26–30^. One possible interpretation of these fate maps is that neural ectoderm cells have the potential to generate all brain regions, but owing to their position, later in development they are fated to encounter anterior- or posterior-inducing signals that only reveal part of their full lineage potential. An open question is whether distinct neural ectoderm cells are already *committed*: i.e., if challenged by ectopic extracellular signals, can they generate brain regions that they normally would not form? Assessing lineage commitment rests on the ability to challenge neural ectoderm cells with various signals, which we attempt here.

Inspired by pioneering embryological studies^12,13,31–33^, the current principal strategy to differentiate hPSCs into neural cells entails inhibition of BMP, TGFβ, and WNT *in vitro*^34–38^. This breakthrough has enabled many successes in generating human forebrain and midbrain cells from hPSCs^34–37,39–57^. Other work has focused on differentiating hPSCs into hindbrain^43,46,58–69^, but we do not fully understand the earliest hindbrain progenitors and the extracellular signals that induce them. To this end, we revisited the first ectodermal precursors in the mouse embryo. As a complementary approach, we also modeled ectoderm differentiation from hPSCs, with the proviso that *in vitro* models may not recreate the full complexity of *in vivo* development.

### Neural and non-neural ectoderm formation

**Figure 1:**
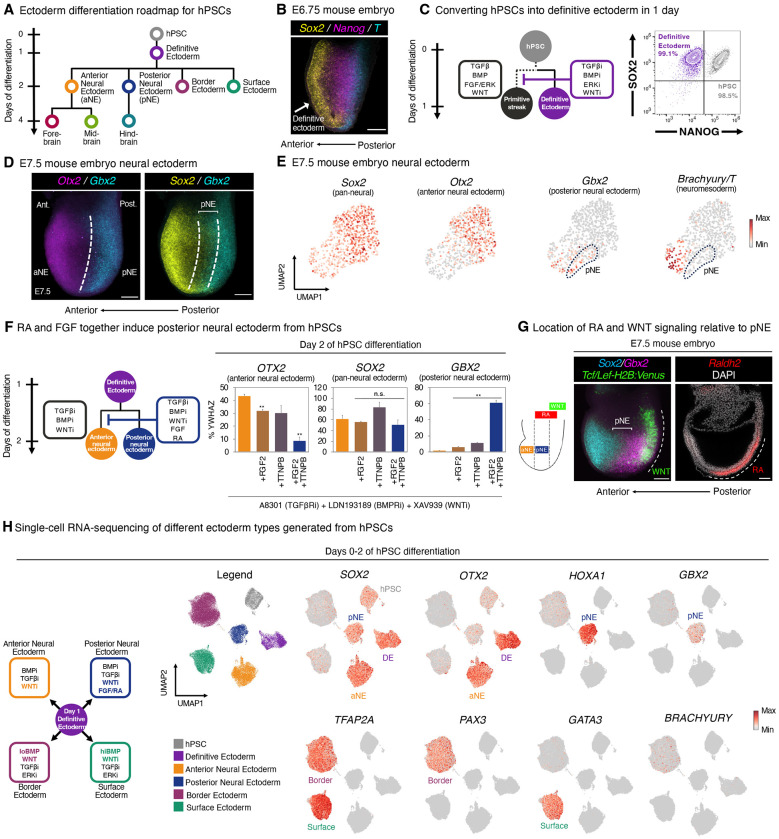
Anterior and posterior neural ectoderm *in vivo* and *in vitro*. A) hPSC differentiation strategy. B) *In situ* staining of E6.75 mouse embryo. Scale: 50 μm. Ant.: anterior. Post.: posterior. C) Flow cytometry of H1 hPSCs prior to, and after, definitive ectoderm differentiation for 24 hours. i: inhibitor. D) *In situ* staining of E7.5 mouse embryo. Scale: 50 μm. aNE: anterior neural ectoderm. pNE: posterior neural ectoderm. E) Analysis of published E7.5 mouse embryo single-cell RNA-sequencing data ^169^. Neural ectoderm cells are shown. F) Quantitative PCR (qPCR) of H1 hPSCs differentiated into definitive ectoderm for 24 hours, and then treated with neural-inducing signals (A-83-01 [1 μM], LDN193189 [250 nM], and XAV939 [1 μM]), in the presence or absence of FGF2 (20 ng/mL) and/or retinoid agonist TTNPB (50 nM), for 24 hours. Gene expression is shown relative to reference gene *YWHAZ*; 100% indicates that a gene is expressed at the same level as *YWHAZ*. G) *In situ* staining of the E7.5 mouse embryo. First, *in situ* staining for *Sox2* and *Gbx2* in a *Tcf/Lef:H2B:Venus* reporter mouse embryo ^173^ shows that *Gbx2*+ *Sox2*+ posterior neural ectoderm is largely devoid of WNT signaling; rather WNT signaling is active in the posterior embryo, where presumptive spinal cord progenitors are located ^25,92–102^ (*left*). A maximum intensity projection of lateral optical sections of a *Tcf/Lef:H2B:Venus* mouse embryo is shown. Second, *in situ* staining shows that *Raldh2/Aldh1a2* (the rate-limiting enzyme in retinoic acid [RA] synthesis) is expressed adjacent to posterior neural ectoderm (*right*), consistent with the notion that posterior neural ectoderm is exposed to RA *in vivo*. Scale bar = 50 μm. H) Single-cell RNA-sequencing of H7 hPSCs differentiated into day-1 definitive ectoderm, day-2 aNE, day-2 pNE, day-2 border ectoderm, or day-2 surface ectoderm. Colors denote differentiation conditions. qPCR data depicts the mean of two biological replicates, with s.e.m. shown. P values were calculated using a t-test. For flow cytometry and *in vitro* staining, a representative image from two biological replicates is shown. *In vitro* scRNAseq data is from a single experiment.

During gastrulation, pluripotent cells first differentiate into either primitive streak or the definitive ectoderm germ layer^16^ (Fig. 1a). *Sox2*+ *Nanog- T*- definitive ectoderm^16,70–72^ arises by E6.75 in the mouse embryo, as shown by multiplexed *in situ* staining (Fig. 1b) and single-cell RNA-sequencing (scRNAseq, Fig. S1a). While BMP, FGF, TGFβ and WNT activation induces primitive streak^73,74^, conversely simultaneous BMP, FGF/ERK, TGFβ and WNT inhibition^34–37,39,75,76^ instead generated 96.5±3.4% pure SOX2+ NANOG- definitive ectoderm within 24 hours of hPSC differentiation (Fig. 1c, Fig. S1b–h). In addition to inhibiting BMP and TGFβ, the suppression of FGF/ERK and WNT signaling further downregulated *NANOG* and drove exit from pluripotency towards ectoderm (Fig. S1b,d,h). hPSC-derived definitive ectoderm appeared to be ectoderm-committed, and failed to respond to endoderm^77^- or paraxial mesoderm^78^-inducing signals (Fig. S1i,j), thereby providing a foundation to subsequently generate ectodermal derivatives.

How do definitive ectoderm cells subsequently adopt neural vs. non-neural ectoderm identities^16^? Within E7.5 mouse embryos, *Sox2*+ neural ectoderm contains two mutually-exclusive *Otx2*+ vs. *Gbx2*+ populations^19,79–81^, as shown by multiplexed *in situ* staining (Fig. 1d, Fig. S1k) and scRNAseq (Fig. 1e) to confirm overlap of neural ectoderm marker *Sox2* with these two other genes. We respectively designate these cell-types as *Sox2*+ *Otx2*+ “anterior neural ectoderm” (aNE) and *Sox2*+ *Gbx2*+ “posterior neural ectoderm” (pNE). The locations of anterior and posterior neural ectoderm in the E7.5 mouse embryo generally comport with the positions of cells fated to respectively generate future forebrain/midbrain vs. hindbrain in classical fate maps^29^. These E7.5 cells represented early neural ectoderm, as they did not express markers of later-stage neural progenitors (*Sox1* and *Pax6*)^72,82^; moreover, posterior neural ectoderm expressed additional posterior markers *Hoxa1* and *Hoxb1*^81,83^ (Fig. S1l). Here we define anterior and posterior neural ectoderm as neural ectoderm that arises during gastrulation; these are distinct from subsequent forebrain, midbrain, and hindbrain neural progenitors that emerge after gastrulation.

What signals induce anterior vs. posterior neural ectoderm *in vitro*? Treating day 1 hPSC-derived definitive ectoderm with prevailing neural induction signals (combined BMP, TGFβ, and WNT inhibition^34–37,39,75^) for 24 hours generated OTX2+ GBX2− anterior neural ectoderm *in vitro* (Fig. 1f, Fig. S1m,n, Fig. S2a). This thus raised the question of what extracellular signals specify GBX2+ posterior neural ectoderm. We found that FGF and RA activation, together with BMP, TGFβ, and WNT blockade, for 24 hours specified GBX2+ OTX2^low^ posterior neural ectoderm (Fig. 1f). FGF and RA were combinatorially required to induce *GBX2*+ posterior neural ectoderm and to repress *OTX2*: either signal alone was insufficient (Fig. 1f, Fig. S2b–d). This thus extends the important roles of FGF and RA in inducing *Gbx2* and repressing *Otx2* in various model organisms^79,84–88^ to human neural ectoderm, and is consistent with how FGF and RA pathway genes (e.g., *Fgf4, Fgf8*, and *Raldh2*) are expressed nearby the E7.5 *Gbx2*+ posterior neural ectoderm *in vivo*^89–91^ (Fig. 1g). The requirement for WNT inhibition to induce posterior neural ectoderm induction *in vitro* (Fig. S2e–i, Fig. S3a) is consistent with the apparent lack of WNT activity in posterior neural ectoderm *in vivo* (Fig. 1g). Instead, WNT signaling was activated in the posterior-most region of the E7.5 mouse embryo (Fig. 1g), which is where *Brachyury* or *Cdx2*—markers of spinal cord or neuromesodermal progenitors—are expressed^92–102^. FGF and RA induced a different type of posterior character than WNT activation, the latter of which has been previously used to differentiate hPSCs into posterior neural cells^43,46,58–60,62,63,66,67,103^ (Fig. S2e,g; Fig. S3a). In particular, posterior neural ectoderm—induced by FGF and RA activation, alongside WNT inhibition—did not express *BRACHYURY* or *CDX2 in vivo* or *in vitro* (Fig. 1e,h; Fig. S3a); these are markers of spinal cord or neuromesodermal progenitors that are known to be induced by WNT^92–102^. Taken together, anterior vs. posterior neural ectoderm arise within 2 days of hPSC differentiation, and we identify the diverging extracellular signals that specify them.

In parallel, we could also differentiate hPSC-derived definitive ectoderm into two types of non-neural ectoderm within 24 hours: PAX3+ TFAP2A^low^ border ectoderm (by WNT activation, alongside low BMP^35,104–107^) and GATA3+ TFAP2A^high^ surface ectoderm (by WNT inhibition, alongside high BMP^35^); both border and surface ectoderm induction also required TGFβ and ERK blockade (Fig. 1h, Fig. S3b–g). This is consistent with how BMP gradients pattern ectoderm into border vs. surface domains *in vivo*^108,109^; we further find that WNT instructs border fate and blocks surface markers (Fig. S3b,c,e). In summary, we generated neural (anterior and posterior neural) and non-neural (border and surface) ectoderm within 2 days of hPSC differentiation, across 4 independent hPSC lines (Fig. 1h, Fig. S4a–d). These two types of neural ectoderm already express anterior or posterior markers, implying early acquisition of region-specific neural identities, which we test below.

### Neural ectoderm lineage tracing *in vivo*

To test the hypothesis that early neural ectoderm cells are already fated to generate either forebrain/midbrain vs. hindbrain *in vivo*^26–30^, we used two complementary genetic lineage-tracing approaches.

**Figure 2:**
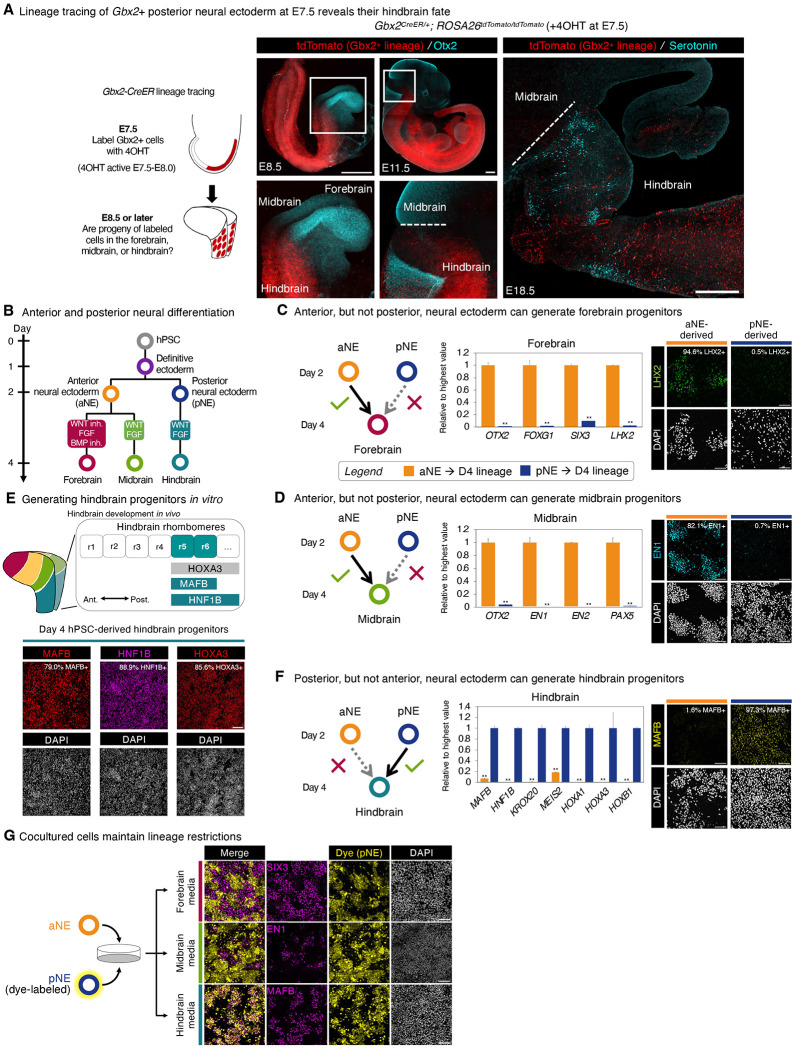
*In vivo* lineage tracing, and *in vitro* lineage commitment assays, of anterior and posterior neural ectoderm. A) E7.5 *Gbx2*^*CreER*^; *ROSA26*^*tdTomato*^ mouse embryos were labeled with 4-hydroxytamoxifen (4OHT) to induce tdTomato expression in pNE. Subsequently, E8.5-E18.5 embryos were stained to visualize *Gbx2*^+^ pNE-derived progeny. The forebrain and midbrain were delimited by *Otx2 in situ* staining (for E8.5) or Otx2 immunostaining (for E11.5). At E18.5, the hindbrain was delimited by immunostaining for serotoninergic neurons present in the hindbrain, but not the midbrain. Scale: 100 μm (for E8.5 and E9.5) and 500 μm (for E11.5 and E18.5). B) hPSC differentiation strategy. C) qPCR (*left*) and immunostaining (*right*) of H1 hPSC-derived day-2 aNE or pNE treated with forebrain-inducing signals for 48 hours. Scale: 120 μm. Gene expression is shown relative to the sample with the highest expression. D) qPCR (*left*) and immunostaining (*right*) of H1 hPSC-derived day-2 aNE or pNE treated with midbrain-inducing signals for 48 hours. Scale: 120 μm. Gene expression is shown relative to the sample with the highest expression. E) Hindbrain gene expression *in vivo* ^4–6^ (*top*). Immunostaining of H1 hPSCs differentiated into definitive ectoderm for 24 hours, followed by posterior neural ectoderm for 24 hours, and hindbrain progenitors for 48 hours (*bottom*). Scale: 250 μm. F) qPCR (*left*) and immunostaining (*right*) of H1 hPSC-derived day-2 aNE or pNE treated with hindbrain-inducing signals for 48 hours. Scale: 120 μm. Gene expression is shown relative to the sample with the highest expression. G) H1 hPSCs were differentiated into either aNE or pNE within 2 days; pNE was fluorescently labeled, and then aNE (uncolored) and pNE (dye-labeled) were mixed. Cocultures were treated with either forebrain-, midbrain-, or hindbrain-inducing signals for 2 additional days, prior to immunostaining. Scale: 100 μm. qPCR data depicts the mean of two biological replicates, with s.e.m. shown. P values were calculated using a t-test. For *in vitro* staining, a representative image from two biological replicates is shown.

First, to test whether during gastrulation, *Gbx2*+ posterior neural ectoderm is already fated to form hindbrain, we crossed a *Gbx2-CreER* driver^110^ to a Cre-dependent *tdTomato* reporter^111^. Past *Gbx2-CreER* lineage-tracing^30^ suggested that *Gbx2*+ cells are posteriorly fated, but exploited tamoxifen, which perdures several days *in vivo*^112,113^ and could thus label *Gbx2*+ hindbrain cells that arise after gastrulation^80,81^. To acutely label E7.5 *Gbx2*+ posterior neural ectoderm, at E7.5 we injected the short-lived tamoxifen metabolite 4-hydroxytamoxifen (4OHT) to achieve a 12-hour labeling window^114,115^ (~E7.5-E8.0). Posterior neural ectoderm-derived tdTomato^+^ progeny were restricted to the E8.5 and E9.5 hindbrain, and were not present in the *Otx2*^+^ forebrain or midbrain (Fig. 2a). At E18.5, posterior neural ectoderm-derived progeny were still restricted to the hindbrain, and were essentially absent from the forebrain and midbrain; we determined the mid/hindbrain boundary by the limit of hindbrain serotonergic neurons (Fig. 2a). Similar results were obtained after 4OHT administration at E7.0 (which would be expected to label from ~E7.0-E7.5) to mark *Gbx2*+ posterior neural ectoderm incipiently emerging at E7.5; in these conditions, posterior neural ectoderm likewise exclusively contributed to the hindbrain, but not the forebrain/midbrain (Fig. S4e,f).

Second, we sparsely labeled *Sox2*+ neural ectoderm cells within the E7.5 mouse embryo (Fig. S4g,h). To this end, we crossed a *Sox2-CreER* driver^116^ to a Cre-dependent multicolor Confetti fluorescent reporter^117^, followed by delivery of a low 4OHT dose to sparsely label E7.5 neural ectoderm cells with one of three detectable colors (red, yellow, or cyan). An advantage of this approach is that it assesses the fate of any *Sox2*+ neural ectoderm progenitor, without regard to whether it already express anterior (*Otx2*) or posterior (*Gbx2*) markers. Analysis of 494 neural ectoderm-derived cell clusters spanning 16 independent embryos revealed each cell cluster was almost exclusively present in either the forebrain/midbrain (62.96%) or hindbrain (32.59%) (Fig. S4g,h). Therefore, by the end of gastrulation, most *Sox2*+ neural ectoderm cells are already fated to become either fore/midbrain or hindbrain.

Lineage tracing reveals the natural fate of progenitors in their native environment, but not their developmental potential ^118^ (i.e., the full range of fates they could adopt if challenged by ectopic signals). It is difficult to assess cellular potential in mammalian embryos *in utero*. Consequently, we turned to an *in vitro* model, whereby hPSC-derived anterior and posterior neural ectoderm were challenged with extracellular signals to test whether they were already committed to form specific brain regions, or whether they retained the potential to adopt alternative fates *in vitro*.

### Studies of lineage commitment *in vitro*

We developed a system to differentiate hPSCs into forebrain, midbrain, and hindbrain progenitors (Fig. 2b). Day 2 anterior neural ectoderm could further bifurcate into *FOXG1*+ *SIX3*+ forebrain progenitors (by blocking posteriorizing signals WNT and BMP, while activating FGF; Fig. 2c, Fig. S5a–d) or *EN1*+ midbrain progenitors (by activating WNT and FGF; Fig. 2d, Fig. S5e–i) within 2 days of further differentiation. This is congruent with the posterior WNT gradient across the developing forebrain and midbrain *in vivo* (Fig. S5j) and how WNT establishes midbrain identity *in vivo*^119–121^ and *in vitro*^34–36,39–41,43–47,56^.

Along the other developmental path, day 2 posterior neural ectoderm could further differentiate into hindbrain progenitors upon combined FGF and WNT activation for 2 days (Fig. 2e,f; Fig. S6a–f). The developing hindbrain is divided into six segments known as rhombomeres^4–6^, and hPSC-derived hindbrain progenitors expressed MAFB, HNF1B, and HOXA3, which together identify rhombomeres 5 and 6 *in vivo*^6,122,123^ (Fig. 2e). Indeed, FGF is likewise required for rhombomere 5 and 6 specification *in vivo*^124,125^. Together, these results reveal that FGF and WNT specify human hindbrain identity, building on observations in model organisms^103,126–128^. To our knowledge, this is the first demonstration that human rhombomere 5 and 6 hindbrain progenitors can be generated *in vitro*, thereby complementing past work differentiating hPSCs into other types of hindbrain progenitor^43,46,58–69^.

Day 2 hPSC-derived anterior vs. posterior neural ectoderm were respectively committed to generating forebrain/midbrain or hindbrain progenitors *in vitro*. Strikingly, when posterior neural ectoderm was treated with forebrain- or midbrain-inducing signals, it largely failed to acquire either of these identities by day 4 (Fig. 2c,d, Fig. S6g). Reciprocally, when anterior neural ectoderm was challenged with hindbrain-inducing conditions, it failed to differentiate into hindbrain by day 4 (Fig. 2f, Fig. S6g, Fig. S7a). Thus, in the *in vitro* conditions tested, anterior and posterior neural ectoderm are both lineage committed. However, it remains formally possible that there may be other experimental interventions that could enable them to interconvert.

Another consideration is that the community effect^129^ could affect the interpretation of these experiments, and we thus tested if hPSC-derived anterior and posterior neural ectoderm remained lineage-committed even when mixed together. hPSC-derived unlabeled anterior neural ectoderm was mixed with fluorescently-labeled posterior neural ectoderm, and these cocultures were then challenged with forebrain-, midbrain-, or hindbrain-inducing signals (Fig. 2g, Fig. S7b). Within these cocultures, anterior neural ectoderm generated SIX3+ forebrain and EN1^+^ midbrain progenitors, but not MAFB+ hindbrain progenitors, and *vice versa* for posterior neural ectoderm, although a few posterior neural ectoderm cells expressed SIX3 in these cocultures (Fig. 2g, Fig. S7b). Therefore, even when cocultured with one another, anterior and posterior neural ectoderm generally obey their respective lineage commitments.

### Divergent chromatin landscapes

**Figure 3:**
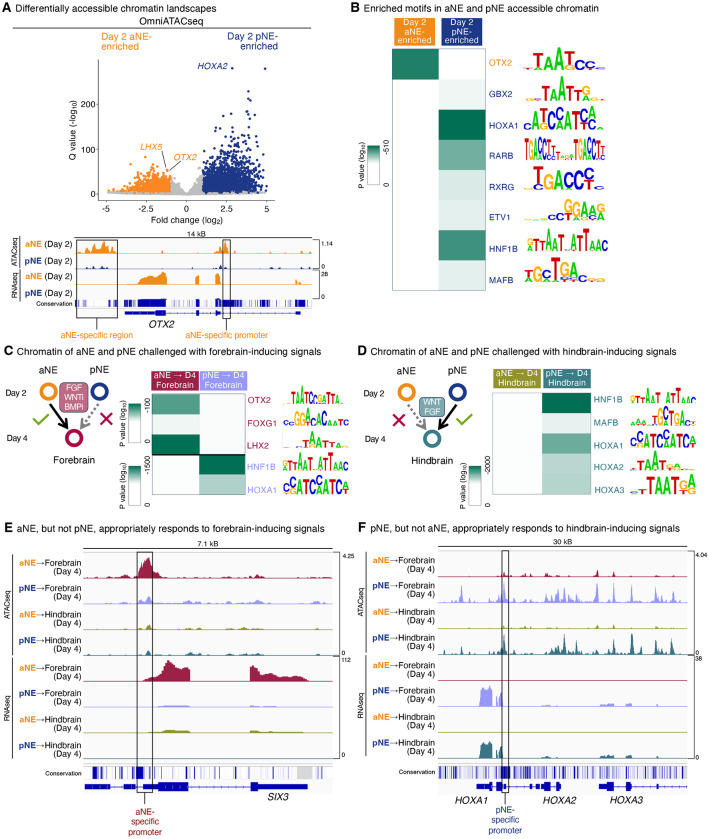
Different accessible chromatin landscapes distinguish hPSC-derived anterior vs. posterior neural ectoderm. A) Differentially accessible chromatin regions in hPSC-derived day-2 aNE or pNE, as detected by OmniATACseq. Top: each dot indicates a single genomic locus, which was assigned to its nearest gene. Bottom: OmniATACseq and bulk-population RNAseq of hPSC-derived aNE or pNE. Conservation: Phastcons evolutionary conservation of genome sequence across 46 vertebrate species. B) Transcription factor motifs that were respectively enriched in either day-2 aNE- or pNE-specific accessible chromatin regions. C) hPSC-derived aNE and pNE were treated with forebrain-inducing signals for 2 days, followed by OmniATACseq to identify chromatin regions that were preferentially accessible in either population. Transcription factor motifs enriched in the accessible chromatin of either aNE or pNE derivatives are shown. D) OmniATACseq and bulk-population RNAseq of hPSC-derived aNE and pNE that were treated with either forebrain- or hindbrain-inducing signals for 2 days. E) hPSC-derived aNE and pNE were treated with hindbrain-inducing signals for 2 days, followed by OmniATACseq to identify chromatin regions that were preferentially accessible in either population. Transcription factor motifs enriched in the accessible chromatin of either aNE or pNE derivatives are shown. F) OmniATACseq and bulk-population RNAseq of hPSC-derived aNE and pNE that were treated with either forebrain-or hindbrain-inducing signals for 2 days. OmniATACseq and bulk-population RNAseq were each performed on two biological replicates. Q values for differential accessibility were calculated using the Wald test, followed by Benjamini-Hochberg adjustment. P values for motif enrichment were calculated using the Fisher exact test.

Anterior and posterior neural ectoderm harbored different accessible chromatin landscapes (Fig. 3a, Fig. S7c, Table S1), foreshadowing their potentials to develop into differing downstream brain lineages. At the genome-wide level, anterior neural ectoderm-enriched accessible chromatin was enriched for OTX2 motifs (Fig. 3b, Table S2). By contrast, posterior neural ectoderm-enriched accessible chromatin was instead enriched for the motifs of future hindbrain transcription factors (HOX, HNF1B, and MAFB), and the motifs of FGF (ETV1) and RA (RARB and RXRG) pathway transcriptional effectors (Fig. 3b, Table S2). This is consistent with how FGF and RA induce posterior neural ectoderm and have key roles in later hindbrain development^4–6^.

How do anterior and posterior neural ectoderm respond at the chromatin level when challenged with developmentally-inappropriate cues? Anterior neural ectoderm treated with forebrain-inducing signals appropriately increased chromatin accessibility at forebrain genes such as *SIX3*, and at the genome-wide level, their accessible chromatin landscape was enriched for key forebrain transcription factor motifs (OTX2, FOXG1, and LHX2; Fig. 3c,e, Fig. S7d, Table S2). Conversely, posterior neural ectoderm challenged with the same forebrain-inducing signals (WNT inhibition and FGF activation) instead adopted a hindbrain-like chromatin landscape. They failed to increase accessibility at forebrain genes (Fig. 3e), and instead increased accessibility at a swath of hindbrain regulatory elements enriched for hindbrain transcription factor motifs (HNF1B and HOX; Fig. 3c, Fig. S7d, e, Table S2). In sum, posterior neural ectoderm resists forebrain-inducing signals, and its chromatin landscape seems committed to subsequently adopt a hindbrain regulatory program.

Anterior neural ectoderm, even when confronted with hindbrain-inducing signals (WNT and FGF activation), instead adopted a forebrain-like regulatory program and failed to increase accessibility at hindbrain genes (e.g., the *HOXA* locus) or hindbrain transcription factor motifs (Fig. 3d,f, Fig. S7e, f, Table S2). On the other hand, posterior neural ectoderm treated with hindbrain-inducing signals activated a hindbrain regulatory program (Fig. 3d,f, Fig. S7e, f, Table S2). Taken together, anterior vs. posterior neural ectoderm have divergent transcriptional and chromatin landscapes that prefigure their respective potentials to subsequently develop into forebrain and hindbrain lineages (Fig. S7g,h). Though it typically takes weeks or months to generate neurons *in vitro*, we propose that cellular competence to generate forebrain/midbrain or hindbrain has already become restricted within the first 2 days of hPSC differentiation.

### Dorsal vs. ventral forebrain and hindbrain

**Figure 4:**
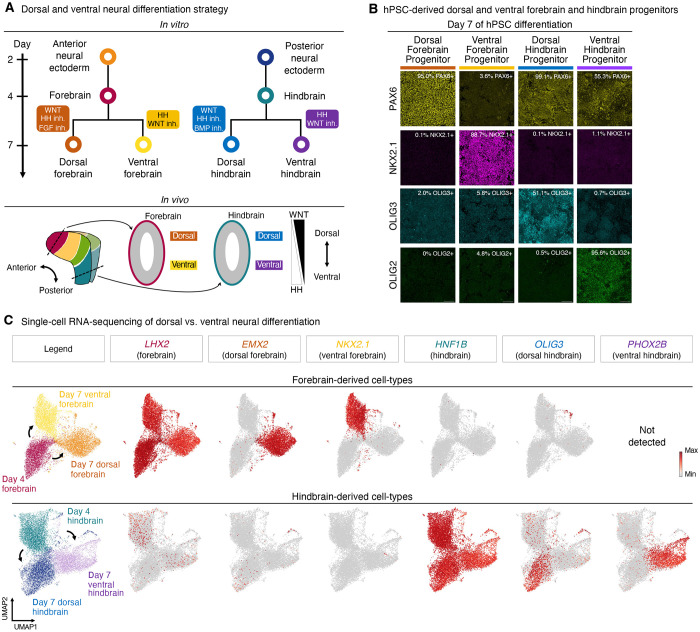
Generation of dorsal forebrain, ventral forebrain, dorsal hindbrain, and ventral hindbrain progenitors from hPSCs *in vitro*. A) Summary of hPSC differentiation *in vitro* (*top*) and brain development *in vivo* (*bottom*). B) Immunostaining of H1 hPSC-derived day-4 forebrain or hindbrain progenitors that were treated with dorsalizing or ventralizing signals for 72 hours. Scale: 120 μm. C) scRNAseq of H7 hPSC-derived day-4 forebrain or hindbrain progenitors, and day-7 dorsal forebrain, ventral forebrain, dorsal hindbrain, or ventral hindbrain progenitors. Colors denote differentiation conditions. For *in vitro* staining, a representative image from two biological replicates is shown. *In vitro* scRNAseq data is from a single experiment.

After its formation, the forebrain undergoes dorsal-ventral patterning into the dorsal forebrain (neocortex, which primarily generates cortical glutamatergic neurons^3,15^) and ventral forebrain (which forms cortical GABAergic interneurons and certain hypothalamic neurons^130,131^) (Fig. 4a). Likewise, the hindbrain also undergoes dorsal-ventral patterning, forming dorsal and ventral hindbrain progenitors that respectively produce hindbrain sensory and motor neurons^132,133^ (Fig. 4a).

Starting from day 4 hPSC-derived forebrain progenitors, we found that WNT activation—combined with HH and FGF inhibition—specified PAX6+ EMX2+ dorsal forebrain progenitors (Fig. 4b,c, Fig. S8a-c). Conversely, HH activation^34^ together with WNT inhibition yielded NKX2.1+ ventral forebrain progenitors (Fig. 4b,c, Fig. S8d). Our *in vitro* results are consistent with how countervailing dorsal WNT vs. ventral HH gradients pattern the forebrain *in vivo*^134–137^. Notably, the requirement for WNT to specify dorsal forebrain agrees with *in vivo* studies^134–137^ and contrasts with prolonged WNT inhibitor treatment to specify hPSC-derived dorsal forebrain progenitors^34–37,39,40^.

Starting from day 4 hPSC-derived hindbrain progenitors, WNT activation together with HH and BMP inhibition generated OLIG3+ PAX3+ dorsal hindbrain progenitors^132,138^ (Fig. 4b,c, Fig. S8e-h). By contrast, application of the converse signals—WNT inhibition and HH activation—generated OLIG2+ NKX6.2+ NKX2.2+ ventral hindbrain progenitors^133,138^ (Fig. 4b,c, Fig. S8g-i). Generation of a given progenitor (e.g., ventral hindbrain) relied on activation of a lineage-instructive signal (e.g., HH), together with simultaneously blocking the signal that induced the opposite lineage (e.g., WNT).

Despite the similarity of the signals added to induce dorsal (WNT) vs. ventral (HH) patterning in forebrain and hindbrain cultures, dorsal and ventral forebrain progenitors minimally expressed hindbrain markers, and *vice versa* (Fig. 4b,c). Therefore, forebrain (anterior neural ectoderm-derived) and hindbrain (posterior neural ectoderm-derived) progenitors continued to maintain their respective identities during dorsal-ventral patterning. This likely reflects the deeply ingrained differences in anterior vs. posterior neural ectoderm laid down earlier during differentiation.

### Making human hindbrain motor neurons

**Figure 5:**
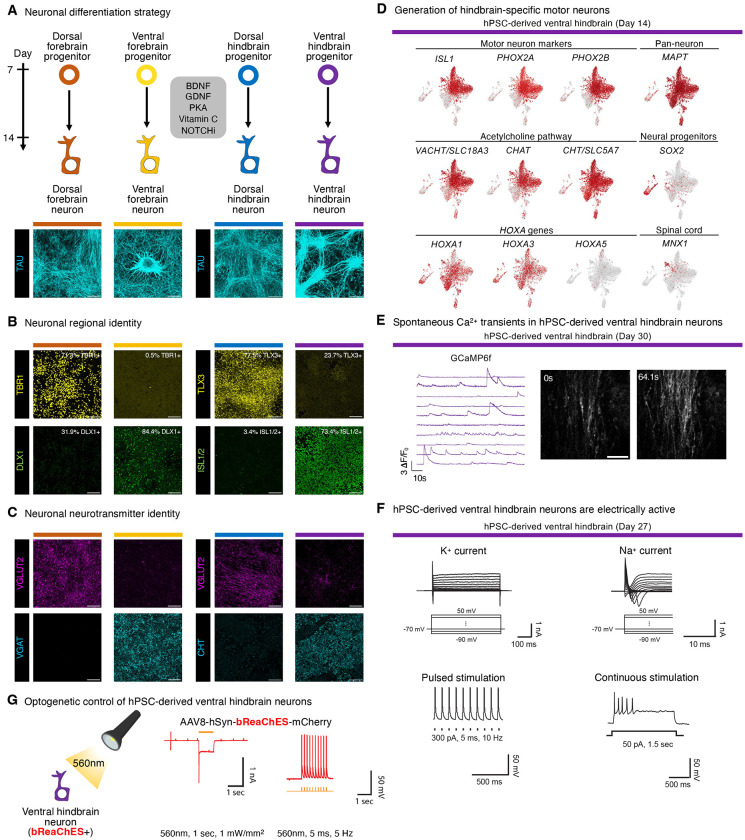
Generation of hindbrain motor neurons from hPSCs *in vitro*. A) Immunostaining of H1 hPSC-derived day-7 dorsal forebrain, ventral forebrain, dorsal hindbrain, or ventral hindbrain progenitors that were exposed to the same neuroninducing signals for 7 additional days *in vitro*. Scale: 120 μm. B) Immunostaining of H1 hPSC-derived day-7 dorsal forebrain, ventral forebrain, dorsal hindbrain, or ventral hindbrain progenitors that were exposed to the same neuroninducing signals for 7 additional days *in vitro*. Scale: 120 μm. C) Immunostaining of H1 hPSC-derived day-7 dorsal forebrain, ventral forebrain, dorsal hindbrain, or ventral hindbrain progenitors that were exposed to the same neuroninducing signals for 7 additional days *in vitro*. Scale: 120 μm. D) scRNAseq of H7 hPSC-derived day-14 ventral hindbrain population. E) Live Ca^2+^ imaging of WTC11 *AAVS1-CAG-GCaMP6f* hPSCs differentiated into ventral hindbrain neurons for 30 days, which exhibited spontaneous Ca^2+^ transients as assessed within individual cells (*left*) and across the culture (*right*). Scale: 50 μm. F) Electrophysiological activity of H1 hPSC-derived ventral hindbrain neurons on day 27 of differentiation, showing voltage-dependent K^+^ or Na^+^ channel currents elicited by depolarization of the holding potential from −90 mV to 50 mV in voltage-clamp mode (*top*) and action potentials elicited by injection of pulsed (300 pA, 5 ms, 10 Hz) or prolonged (50 pA, 1.5 seconds) currents in current-clamp mode (*bottom*). G) Day 28 H1 hPSC-derived ventral hindbrain neurons transduced with AAV8-*hSyn-bReaChES-mCherry* were stimulated with either prolonged (1 second), or pulses of (5 ms, 5 Hz), 560nm light, and electrophysiological recording was performed to detect action potentials. For *in vitro* staining, a representative image from two biological replicates is shown. *In vitro* scRNAseq data is from a single experiment. Ca^2+^ imaging and electrophysiology were each performed on a single experiment.

These four different neural progenitors (day-7 hPSC-derived dorsal forebrain, ventral forebrain, dorsal hindbrain and ventral hindbrain) could further differentiate into mutually-exclusive types of neuron (Fig. 5a). To this end, we provided neurotrophic factors (BDNF, GDNF, Forskolin and Vitamin C) while blocking NOTCH (to drive neural progenitors out of the cell cycle and into postmitotic neurons) for 7 additional days^139^, generating *MAPT*+ *SNAP25*+ neurons alongside some remaining undifferentiated *SOX2*+ neural progenitors (Fig. 5a, Fig. S9a). Dorsal forebrain progenitors exposed to these neuron-inducing signals differentiated into *TBR1*+ cortical glutamatergic Cajal-Retzius neurons (Fig. 5b,c, Fig. S9a,b). Conversely, ventral forebrain progenitors instead formed *DLX1*+ cortical interneurons, in addition to hypothalamic-like *OTP*+ and *ISL1*+ neurons that respectively expressed the *ADCYAP1*^140,141^ and *POMC*^142^ neuropeptide genes (Fig. 5b,c, Fig. S9a, c, d). This could enable *in vitro* modeling of hunger-suppressing *POMC*+ hypothalamic neurons; their importance *in vivo* is underscored by the severe obesity of *POMC*-deficient humans and mice^142^.

In parallel, we differentiated hPSCs into hindbrain neurons. Hindbrain motor neurons project through the cranial nerves and secrete acetylcholine to control face and neck muscles^4–6^. While past work has differentiated hPSCs into certain types of hindbrain neuron^43,46,58–69^, it has remained challenging to generate human rhombomere 6-specific motor neurons, which project through cranial nerve IX to control muscles crucial for swallowing^5^. To meet this challenge, we differentiated hPSC-derived ventral hindbrain progenitors into hindbrain motor neurons that expressed hallmark transcription factor *ISL1*^143^ and acetylcholine pathway genes (*VACHT, CHAT* and *CHT*) (Fig. 5b,c, Fig. S9a,e). Cholinergic motor neurons reside in both the hindbrain and spinal cord, but our hPSC-derived hindbrain motor neurons expressed hallmarks of hindbrain rhombomere 5/6 identity (*HOXA1, HOXA3, PHOX2A*, and *PHOX2B*)^4–6,144^, while minimally expressing spinal cord markers (*HOXA5* and *MNX1/HB9*; Fig. 5d). These hindbrain motor neurons arose alongside smaller subsets of other ventral hindbrain neuron subtypes (Fig. S9f). In contrast, dorsal hindbrain progenitors exposed to the same neuron-inducing signals instead generated *TLX3*+ *LBX1*+ somatosensory-like neurons, which are crucial for sensory information reception and processing *in vivo*^132,145^ (Fig. 5b, Fig. S9g). In short, we generated hPSC-derived ventral and dorsal hindbrain neurons that continued to express rhombomere 5/6-specific markers.

hPSC-derived ventral hindbrain neurons were electrophysiologically active. They displayed spontaneous Ca^2+^ transients, as measured by GCaMP6f^146^ (Fig. 5e, Fig. S10a–c) and fired action potentials when injected with depolarizing currents (Fig. 5f) or optogenetically stimulated with the red-shifted excitatory opsin bReaChES^147^ (Fig. 5g, Fig. S10d). Similar results were observed with hPSC-derived dorsal hindbrain neurons (Fig. S10e–g). Posterior neural ectoderm thus provides a path to generate electrophysiologically-active hindbrain motor neurons *in vitro*.

### Evolutionary conservation

**Figure 6:**
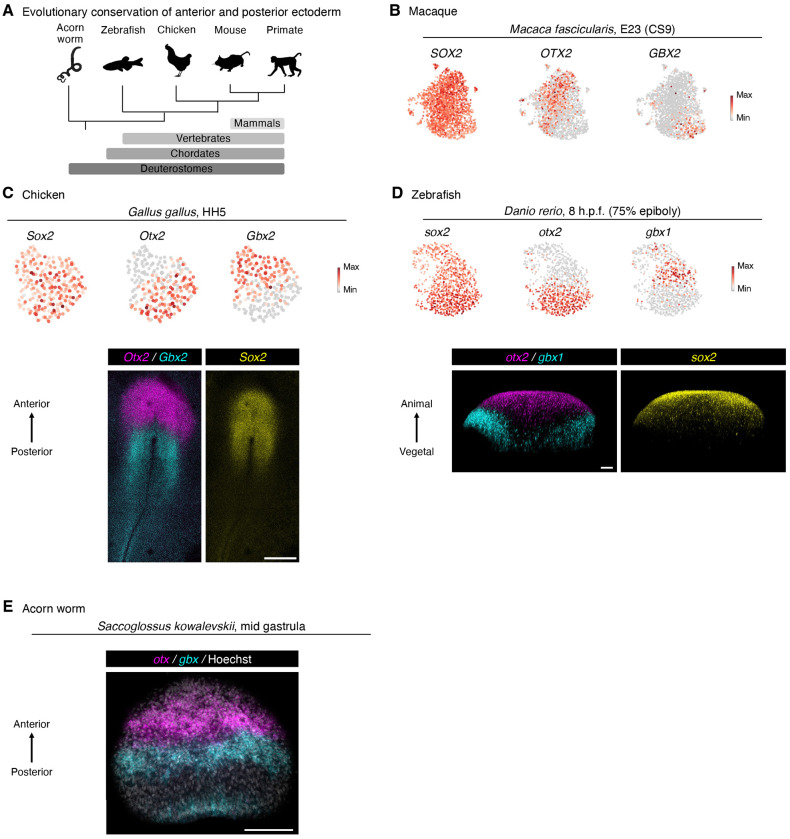
Evolutionary conservation of anterior and posterior ectoderm. A) Summary of evolutionary relationships. B) Single-cell RNA-sequencing of gastrulating macaque embryo (Carnegie Stage 9 [CS9]; embryonic day 23 [E23]); analysis performed on a previously-published resource ^170^. Neural ectoderm cells are shown. C) Single-cell RNA-sequencing of gastrulating chicken embryo (Hamburger-Hamilton stage 5 [HH5]); analysis performed on a previously-published resource ^171^. Neural ectoderm cells are shown (*top*). *In situ* staining of HH5 stage chicken embryo. Scale: 500 μm (*bottom*). D) Single-cell RNA-sequencing of gastrulating zebrafish embryo (8 hours post-fertilization [h.p.f.], 75% epiboly); analysis performed on a previously-published resource ^172^. Neural ectoderm cells are shown (*top*). *In situ* staining of 8 h.p.f. zebrafish embryo, scale bar: 50 μm (*bottom*). E) *In situ* staining of gastrulating acorn worm embryo. A maximum intensity projection of optical sections focusing on the ectoderm is shown. Scale: 100 μm. Staining was performed on a single embryo of each species.

Finally, given that anterior and posterior neural ectoderm arise within the gastrulating mouse embryo, do similar progenitors exist in other species (Fig. 6a)? Building on past studies^28,85,148–150^, we found separate *Otx*+ *Sox2*+ anterior and *Gbx*+ *Sox2*+ posterior neural ectoderm populations in the gastrulating embryos of multiple vertebrate species, including macaque (Fig. 6b), chicken (Fig. 6c), and zebrafish (Fig. 6d). Multiplexed *in situ* staining of chicken and zebrafish embryos confirmed mutually-exclusive *Otx2*+ and *Gbx2*+ subsets of *Sox2*+ neural ectoderm cells (Fig. 6c,d). We additionally found that mutually-exclusive *otx*+ vs. *gbx*+ ectoderm arose during blastulation and gastrulation in a hemichordate lineage, the acorn worm *Saccoglossus kowalevskii*^151^ (Fig. 6e, Fig. S10h). Hemichordates are closely related to chordates, and shared a common ancestor ~550-600 million years ago with the other deuterostome species examined here^152^. Our discovery of posterior ectoderm in gastrulating acorn worms is notable, as our prior work revealed a hindbrain-like molecular program in this non-chordate species^153^. Taken together, these results intimate that two parallel ectodermal progenitors might be deeply evolutionarily conserved across deuterostomes.

## Discussion

At the crux of this work is the question of when forebrain/midbrain vs. hindbrain identities become separated during embryonic development. In one model, neural ectoderm represents a common progenitor for the entire brain^20–25^. By contrast, classical fate maps suggested that even as early as gastrulation, distinct neural ectoderm cells are already fated to form different brain regions^26–30^. However, these pioneering fate mapping studies did not test whether neural ectoderm cells are already committed to form specific brain regions (which requires the ability to isolate neural ectoderm cells and challenge them in various conditions) nor the genomic mechanisms underlying lineage restriction. Our data suggest that anterior and posterior neural ectoderm are already committed to form different brain regions *in vitro*. When challenged with hindbrain-inducing signals *in vitro*, hPSC-derived anterior neural ectoderm cannot “transdifferentiate” into hindbrain. Reciprocally, hPSC-derived posterior neural ectoderm cannot “transdifferentiate” into forebrain/midbrain. Mechanistically, hPSC-derived anterior and posterior neural ectoderm harbor divergent accessible chromatin landscapes, which foreshadow their different lineage potentials and furthermore resist inappropriate lineage-inducing signals.

More broadly speaking, our work relates to the longstanding question in developmental biology of how and when different regions of the central nervous system diversify from one another. Important studies have challenged the assumption that there is a common central nervous system progenitor, and have instead discovered separate progenitors to the brain vs. spinal cord (the latter called “neuromesoderm”)^25,92–102^. There are thus separate anterior (brain-specific) and posterior (spinal cord-specific) precursors that form the central nervous system^25,92–102^. We suggest further complexity: although the brain is often construed as a single organ, the brain may itself derive from two parallel progenitors that emerge early in development and that respectively form the forebrain/midbrain vs. the hindbrain. Analogously, the heart derives from two parallel progenitors, the first and second heart fields, which converge to form a single organ^154^. Taken together, the central nervous system may arise from the coalescence of multiple region-specific progenitors that separately give rise to the forebrain/midbrain (anterior neural ectoderm), hindbrain (posterior neural ectoderm), and spinal cord (neuromesoderm^25,92–102^). However, our results cannot exclude the possibility that a “pan-brain” progenitor exists for a very brief duration *in vivo*, between the emergence of the ectoderm germ layer (~E6.75-E7.0)^16^ and the bifurcation of anterior vs. posterior neural ectoderm (~E7.5).

Classical embryological studies generally support our model that anterior and posterior neural ectoderm represent parallel progenitors. Early forebrain and midbrain—which are both anterior neural ectoderm-derived lineages—can interconvert *in vivo* if confronted with various experimental conditions; however, in these studies, forebrain and midbrain could not apparently adopt hindbrain identity^155–158^. Therefore, anterior and posterior neural ectoderm cannot readily interconvert under normal conditions. However, earlier perturbations that may affect the initial emergence of anterior vs. posterior neural ectoderm during gastrulation—such as manipulating transcription factors that specify anterior vs. posterior neural ectoderm identity (e.g., *Otx2*)^159,160^ or deleting *Fgf8* (which is required for posterior neural ectoderm formation)^30,84^—alters the subsequent acquisition of midbrain vs. hindbrain identities *in vivo*. Perturbing early anterior and posterior neural ectoderm specification thus impacts the development of their downstream progeny. However, it remains to be determined whether anterior and posterior neural ectoderm are lineage committed *in vivo*; this is technically challenging, as it would entail transplanting purified, labeled populations of these progenitor cells into ectopic locations in the gastrulating mammalian embryo.

We find that the fundamental bifurcation between anterior vs. posterior neural ectoderm occurs surprisingly early, within the first 2 days of hPSC differentiation, long preceding the emergence of classically-defined neural progenitors or neurons. This parallels how these anterior vs. posterior neural ectoderm rapidly arise from pluripotent cells *in vivo* within 2 days, between E5.5 to E7.5. Influential studies previously demonstrated that hPSCs can be differentiated into two different types of anterior vs. posterior neural progenitor within 2 weeks *in vitro*, which express the classical neural progenitor markers PAX6 and SOX1^161,162^. However, the bifurcation of anterior vs. posterior neural ectoderm occurs earlier during development, taking place during gastrulation and preceding the expression of neural progenitor markers *Pax6* or *Sox1*^72,163^ at later developmental stages.

Defining and manipulating the early fundamental lineage decision between anterior vs. posterior neural ectoderm is paramount to precisely direct stem cells toward a hindbrain developmental path, in preference to a forebrain/midbrain lineage. If this early time window is missed, and differentiating stem cells proceed down the “wrong” developmental track, they cannot seem to readily crossover to adopt a different brain regional identity. This emphasizes the importance of the early bifurcation of anterior vs. posterior neural ectoderm, and reveals hitherto-cryptic diversity among the earliest human neural ectoderm cells *in vitro*.

Posterior neural ectoderm provides a platform to subsequently generate certain types of hindbrain neuron *in vitro*. We find that prevailing signals used to differentiate hPSCs towards neural fates (BMP, TGFβ, and WNT inhibition)^34–37,75,164–168^ generate anterior neural ectoderm, explaining the past success that the field has enjoyed in creating forebrain and midbrain neurons^1–3^. Conversely, we found that simultaneous FGF and RA activation alongside neural induction signals (BMP, TGFβ, and WNT inhibition) specified posterior neural ectoderm. We subsequently differentiated these precursors into hindbrain progenitors and hindbrain motor neurons corresponding to rhombomeres 5/6, which were electrophysiologically active and expressed defining hindbrain-specific transcription factors (*PHOX2A, PHOX2B* and anterior *HOX* genes)^4–6,144^ and acetylcholine pathway genes. This thus complements past work that successfully differentiated hPSCs into other types of hindbrain neuron^43,46,58–69^. In particular, the ability to create human rhombomere 5/6-specific motor neurons—which are crucial for swallowing *in vivo*—could provide a powerful platform to study SMA, ALS, and other neurodegenerative diseases wherein impaired swallowing can lead to choking and death^9–11^. More broadly, the hindbrain is essential for breathing, eating, sleep, wakefulness, and other life-critical functions^4–6^, and the ability to create human hindbrain neurons holds promise for modeling other deadly diseases that affect the hindbrain^7,8^.

Our results clearly show that recapitulating the initial bifurcation of anterior vs. posterior neural ectoderm is critical to differentiate hPSCs into neurons in a manner that reflects anterior-posterior regional identities (e.g., forebrain, midbrain, or hindbrain). Separate anterior and posterior ectoderm populations arise during gastrulation across deuterostome species as diverse as acorn worm, zebrafish, chicken, mouse, and primate, implying that this distinction between two different types of ectoderm predates the origins of chordates and arose ~550-600 million years ago^152^. We conclude that the emerging notion of two parallel brain progenitors has ramifications for development, differentiation, and evolution.

## Supplementary Material

1
