# Supplementary material for: Two parallel lineage-committed progenitors contribute to the developing brain
Source: bioRxiv. 2025 Jul 5:2025.07.02.662771. Preprint. [Version 2] doi: 10.1101/2025.07.02.662771 (PMC12236623; doi:10.1101/2025.07.02.662771)
Supplement: 1 [file NIHPP2025.07.02.662771v2-supplement-1.pdf]

Figure S1

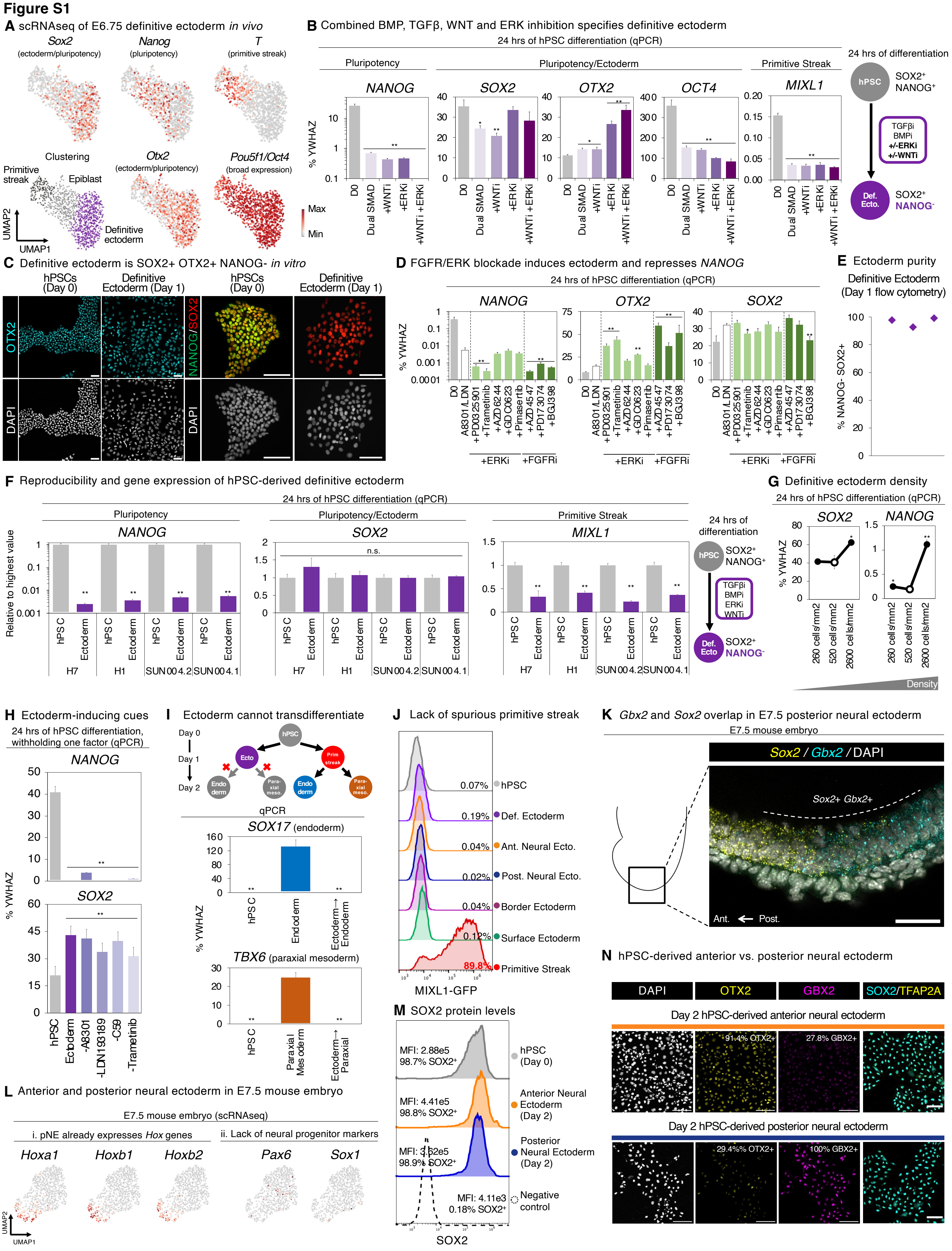
A scRNAseq of E6.75 definitive ectoderm in vivo
Sox2 (ectoderm/pluripotency)
Nanog (pluripotency)
T (primitive streak)
Clustering
Primitive streak
Epiblast
Definitive ectoderm
UMAP2
UMAP1
Otx2 (ectoderm/pluripotency)
Pou5f1/Oct4 (broad expression)
Max
Min
B Combined BMP, TGFβ, WNT and ERK inhibition specifies definitive ectoderm
24 hrs of hPSC differentiation (qPCR)
Pluripotency
Pluripotency/Ectoderm
Primitive Streak
NANOG
SOX2
OTX2
OCT4
MIXL1
% YWHAZ
D0
Dual SMAD
+WNTi
+ERKi
+WNTi +ERKi
24 hrs of differentiation
hPSC
SOX2+ NANOG+
TGFβi
BMPi
+/-ERKi
+/-WNTi
Def. Ecto.
SOX2+ NANOG-
C Definitive ectoderm is SOX2+ OTX2+ NANOG- in vitro
hPSCs (Day 0)
Definitive Ectoderm (Day 1)
OTX2
DAPI
NANOG/SOX2
D FGFR/ERK blockade induces ectoderm and represses NANOG
24 hrs of hPSC differentiation (qPCR)
NANOG
OTX2
SOX2
% YWHAZ
D0
A8301/LDN
+PD0325901
+Trametinib
+AZD6244
+GDC0623
+Pimasertib
+AZD4547
+PD173074
+BGJ398
+ERKi
+FGFRi
E Ectoderm purity
Definitive Ectoderm (Day 1 flow cytometry)
% NANOG- SOX2+
F Reproducibility and gene expression of hPSC-derived definitive ectoderm
24 hrs of hPSC differentiation (qPCR)
Pluripotency
Pluripotency/Ectoderm
Primitive Streak
NANOG
SOX2
MIXL1
Relative to highest value
n.s.
hPSC
Ectoderm
H7
H1
SUN004.2
SUN004.1
24 hrs of differentiation
hPSC
SOX2+ NANOG+
TGFβi
BMPi
ERKi
WNTi
Def. Ecto
SOX2+ NANOG-
G Definitive ectoderm density
24 hrs of hPSC differentiation (qPCR)
SOX2
NANOG
% YWHAZ
260 cells/mm2
520 cells/mm2
2600 cells/mm2
Density
H Ectoderm-inducing cues
24 hrs of hPSC differentiation, withholding one factor (qPCR)
NANOG
SOX2
% YWHAZ
hPSC
Ectoderm
-A8301
-LDN193189
-C59
-Trametinib
I Ectoderm cannot transdifferentiate
Day 0
Day 1
Day 2
hPSC
Ecto
Prim streak
Endo derm
Para-xial meso.
qPCR
SOX17 (endoderm)
TBX6 (paraxial mesoderm)
% YWHAZ
hPSC
Endoderm
Ectoderm→Endoderm
Paraxial Mesoderm
Ectoderm→Paraxial
J Lack of spurious primitive streak
0.07% hPSC
0.19% Def. Ectoderm
0.04% Ant. Neural Ecto.
0.02% Post. Neural Ecto.
0.04% Border Ectoderm
0.12% Surface Ectoderm
89.8% Primitive Streak
MIXL1-GFP
K Gbx2 and Sox2 overlap in E7.5 posterior neural ectoderm
E7.5 mouse embryo
Sox2 / Gbx2 / DAPI
Sox2+ Gbx2+
Ant. ← Post.
L Anterior and posterior neural ectoderm in E7.5 mouse embryo
E7.5 mouse embryo (scRNAseq)
i. pNE already expresses Hox genes
ii. Lack of neural progenitor markers
Hoxa1
Hoxb1
Hoxb2
Pax6
Sox1
UMAP2
UMAP1
M SOX2 protein levels
MFI: 2.88e5 98.7% SOX2+
hPSC (Day 0)
MFI: 4.41e5 98.8% SOX2+
Anterior Neural Ectoderm (Day 2)
MFI: 3.62e5 98.9% SOX2+
Posterior Neural Ectoderm (Day 2)
MFI: 4.11e3 0.18% SOX2+
Negative control
SOX2
N hPSC-derived anterior vs. posterior neural ectoderm
DAPI
OTX2
GBX2
SOX2/TFAP2A
Day 2 hPSC-derived anterior neural ectoderm
91.4% OTX2+
27.8% GBX2+
Day 2 hPSC-derived posterior neural ectoderm
29.4%% OTX2+
100% GBX2+

Figure S2

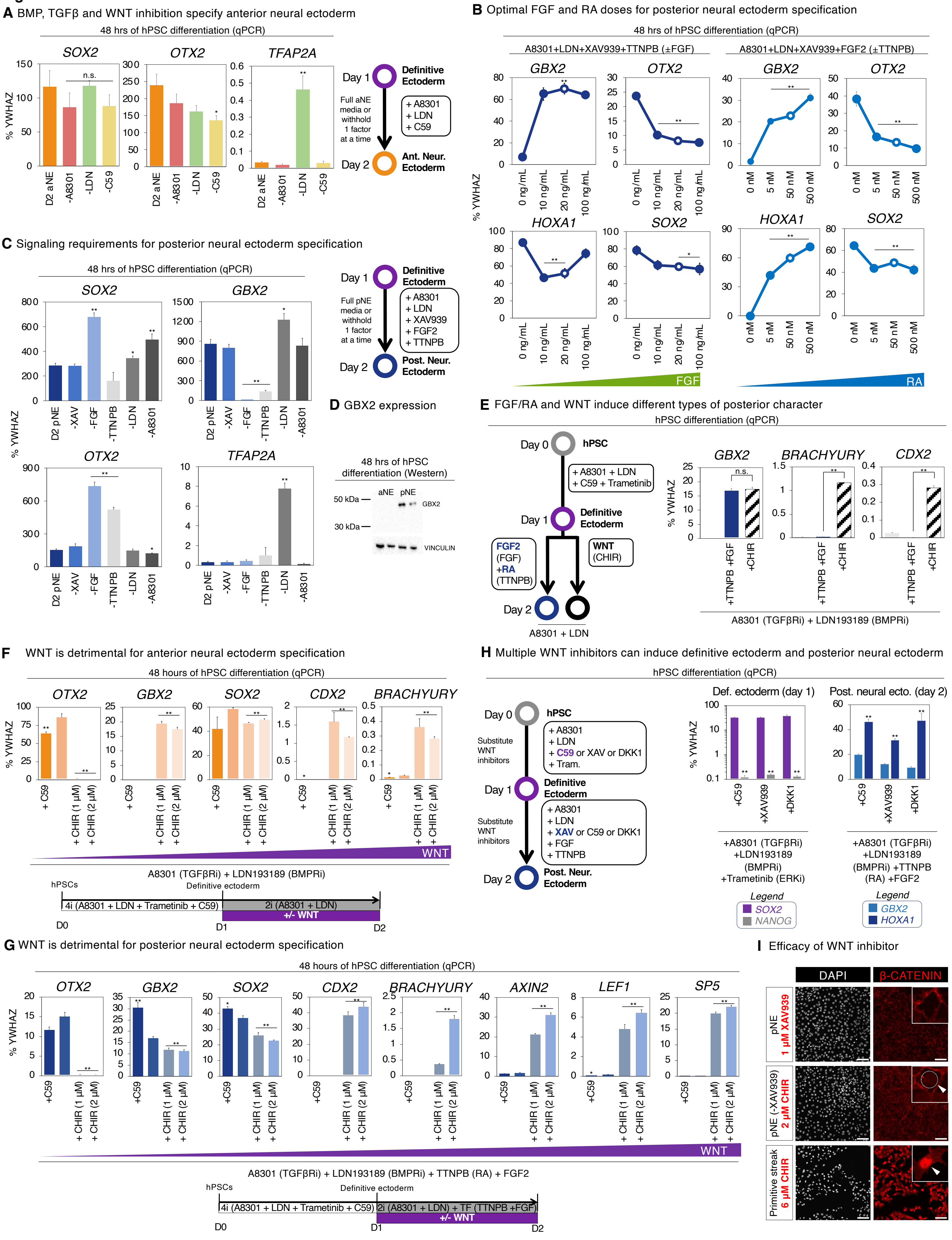
A BMP, TGFβ and WNT inhibition specify anterior neural ectoderm
48 hrs of hPSC differentiation (qPCR)
SOX2
OTX2
TFAP2A
% YWHAZ
n.s.
D2 aNE
-A8301
-LDN
-C59
Day 1
Definitive Ectoderm
Full aNE media or withhold 1 factor at a time
+ A8301
+ LDN
+ C59
Day 2
Ant. Neur. Ectoderm
B Optimal FGF and RA doses for posterior neural ectoderm specification
48 hrs of hPSC differentiation (qPCR)
A8301+LDN+XAV939+TTNPB (±FGF)
A8301+LDN+XAV939+FGF2 (±TTNPB)
GBX2
OTX2
HOXA1
SOX2
% YWHAZ
0 ng/mL
10 ng/mL
20 ng/mL
100 ng/mL
0 nM
5 nM
50 nM
500 nM
FGF
RA
C Signaling requirements for posterior neural ectoderm specification
48 hrs of hPSC differentiation (qPCR)
SOX2
GBX2
OTX2
TFAP2A
% YWHAZ
D2 pNE
-XAV
-FGF
-TTNPB
-LDN
-A8301
Day 1
Definitive Ectoderm
Full pNE media or withhold 1 factor at a time
+ A8301
+ LDN
+ XAV939
+ FGF2
+ TTNPB
Day 2
Post. Neur. Ectoderm
D GBX2 expression
48 hrs of hPSC differentiation (Western)
aNE
pNE
50 kDa
30 kDa
GBX2
VINCULIN
E FGF/RA and WNT induce different types of posterior character
hPSC differentiation (qPCR)
Day 0
hPSC
+ A8301 + LDN
+ C59 + Trametinib
Day 1
Definitive Ectoderm
FGF2 (FGF)
+RA (TTNPB)
WNT (CHIR)
Day 2
A8301 + LDN
GBX2
BRACHYURY
CDX2
% YWHAZ
n.s.
+TTNPB +FGF
+CHIR
A8301 (TGFβRi) + LDN193189 (BMPRi)
F WNT is detrimental for anterior neural ectoderm specification
48 hours of hPSC differentiation (qPCR)
OTX2
GBX2
SOX2
CDX2
BRACHYURY
% YWHAZ
+ C59
+ CHIR (1 µM)
+ CHIR (2 µM)
WNT
A8301 (TGFβRi) + LDN193189 (BMPRi)
hPSCs
Definitive ectoderm
4i (A8301 + LDN + Trametinib + C59)
2i (A8301 + LDN)
+/- WNT
D0
D1
D2
H Multiple WNT inhibitors can induce definitive ectoderm and posterior neural ectoderm
hPSC differentiation (qPCR)
Day 0
hPSC
Substitute WNT inhibitors
+ A8301
+ LDN
+ C59 or XAV or DKK1
+ Tram.
Day 1
Definitive Ectoderm
Substitute WNT inhibitors
+ A8301
+ LDN
+ XAV or C59 or DKK1
+ FGF
+ TTNPB
Day 2
Post. Neur. Ectoderm
Def. ectoderm (day 1)
Post. neural ecto. (day 2)
% YWHAZ
+C59
+XAV939
+DKK1
+A8301 (TGFβRi)
+LDN193189 (BMPRi)
+Trametinib (ERKi)
+A8301 (TGFβRi)
+LDN193189 (BMPRi) +TTNPB (RA) +FGF2
Legend
SOX2
NANOG
Legend
GBX2
HOXA1
G WNT is detrimental for posterior neural ectoderm specification
48 hours of hPSC differentiation (qPCR)
OTX2
GBX2
SOX2
CDX2
BRACHYURY
AXIN2
LEF1
SP5
% YWHAZ
+C59
+ CHIR (1 µM)
+ CHIR (2 µM)
WNT
A8301 (TGFβRi) + LDN193189 (BMPRi) + TTNPB (RA) + FGF2
hPSCs
Definitive ectoderm
4i (A8301 + LDN + Trametinib + C59)
2i (A8301 + LDN) + TF (TTNPB +FGF)
+/- WNT
D0
D1
D2
I Efficacy of WNT inhibitor
DAPI
β-CATENIN
pNE
1 µM XAV939
pNE (-XAV939)
2 µM CHIR
Primitive streak
6 µM CHIR

Figure S3

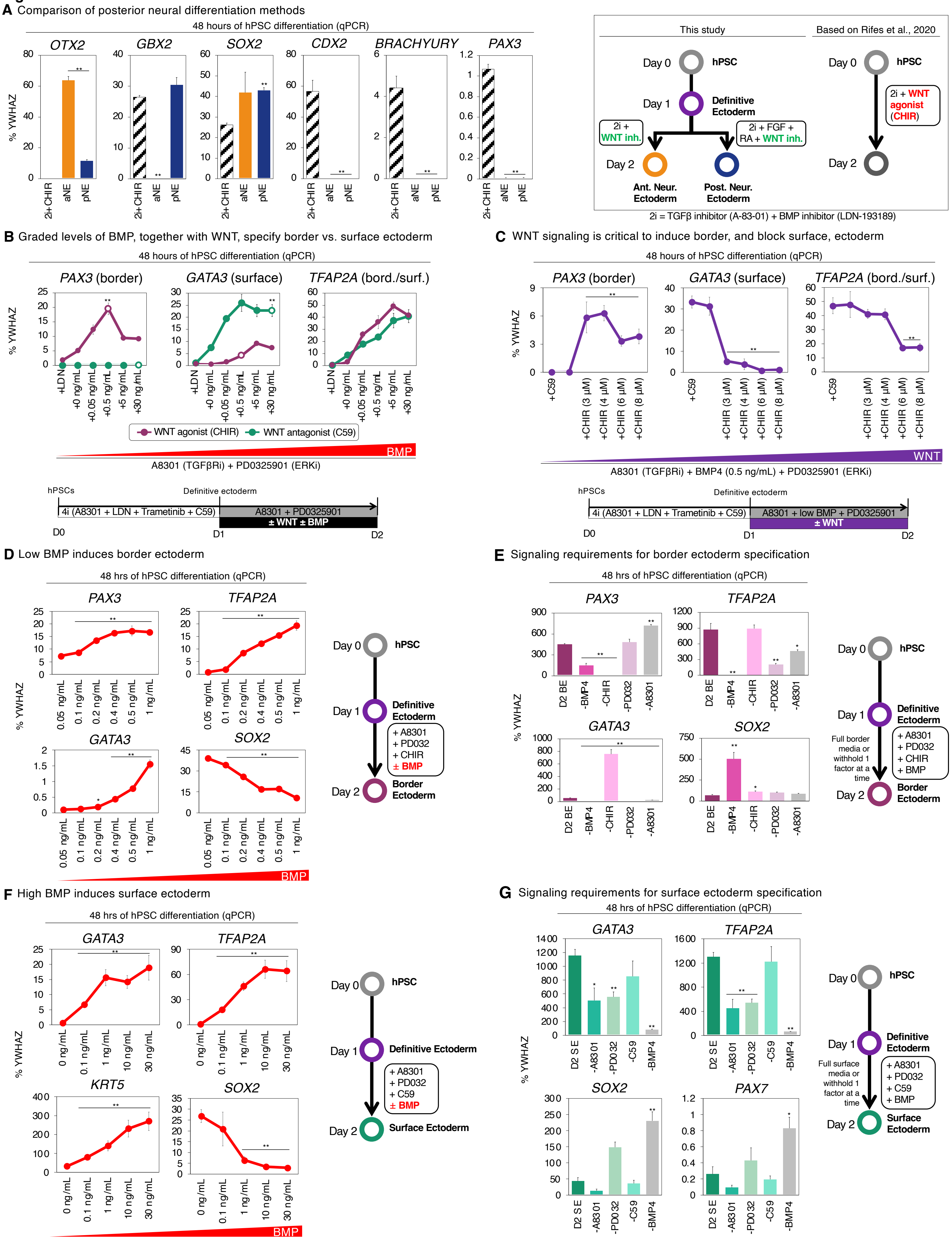
A Comparison of posterior neural differentiation methods
48 hours of hPSC differentiation (qPCR)
OTX2
GBX2
SOX2
CDX2
BRACHYURY
PAX3
% YWHAZ
2i+ CHIR
aNE
pNE
This study
Based on Rifes et al., 2020
Day 0
hPSC
Day 1
Definitive Ectoderm
2i + WNT inh.
2i + FGF + RA + WNT inh.
Day 2
Ant. Neur. Ectoderm
Post. Neur. Ectoderm
2i + WNT agonist (CHIR)
2i = TGFβ inhibitor (A-83-01) + BMP inhibitor (LDN-193189)
B Graded levels of BMP, together with WNT, specify border vs. surface ectoderm
48 hours of hPSC differentiation (qPCR)
PAX3 (border)
GATA3 (surface)
TFAP2A (bord./surf.)
+LDN
+0 ng/mL
+0.05 ng/mL
+0.5 ng/mL
+5 ng/mL
+30 ng/mL
WNT agonist (CHIR)
WNT antagonist (C59)
BMP
A8301 (TGFβRi) + PD0325901 (ERKi)
hPSCs
Definitive ectoderm
4i (A8301 + LDN + Trametinib + C59)
A8301 + PD0325901
± WNT ± BMP
D0
D1
D2
C WNT signaling is critical to induce border, and block surface, ectoderm
48 hours of hPSC differentiation (qPCR)
PAX3 (border)
GATA3 (surface)
TFAP2A (bord./surf.)
+C59
+CHIR (3 μM)
+CHIR (4 μM)
+CHIR (6 μM)
+CHIR (8 μM)
WNT
A8301 (TGFβRi) + BMP4 (0.5 ng/mL) + PD0325901 (ERKi)
hPSCs
Definitive ectoderm
4i (A8301 + LDN + Trametinib + C59)
A8301 + low BMP + PD0325901
± WNT
D0
D1
D2
D Low BMP induces border ectoderm
48 hrs of hPSC differentiation (qPCR)
PAX3
TFAP2A
GATA3
SOX2
0.05 ng/mL
0.1 ng/mL
0.2 ng/mL
0.4 ng/mL
0.5 ng/mL
1 ng/mL
BMP
Day 0
hPSC
Day 1
Definitive Ectoderm
+ A8301
+ PD032
+ CHIR
± BMP
Day 2
Border Ectoderm
E Signaling requirements for border ectoderm specification
48 hrs of hPSC differentiation (qPCR)
PAX3
TFAP2A
GATA3
SOX2
D2 BE
-BMP4
-CHIR
-PD032
-A8301
Day 0
hPSC
Day 1
Definitive Ectoderm
Full border media or withhold 1 factor at a time
+ A8301
+ PD032
+ CHIR
+ BMP
Day 2
Border Ectoderm
F High BMP induces surface ectoderm
48 hrs of hPSC differentiation (qPCR)
GATA3
TFAP2A
KRT5
SOX2
0 ng/mL
0.1 ng/mL
1 ng/mL
10 ng/mL
30 ng/mL
BMP
Day 0
hPSC
Day 1
Definitive Ectoderm
+ A8301
+ PD032
+ C59
± BMP
Day 2
Surface Ectoderm
G Signaling requirements for surface ectoderm specification
48 hrs of hPSC differentiation (qPCR)
GATA3
TFAP2A
SOX2
PAX7
D2 SE
-A8301
-PD032
-C59
-BMP4
Day 0
hPSC
Day 1
Definitive Ectoderm
Full surface media or withhold 1 factor at a time
+ A8301
+ PD032
+ C59
+ BMP
Day 2
Surface Ectoderm

Figure S4

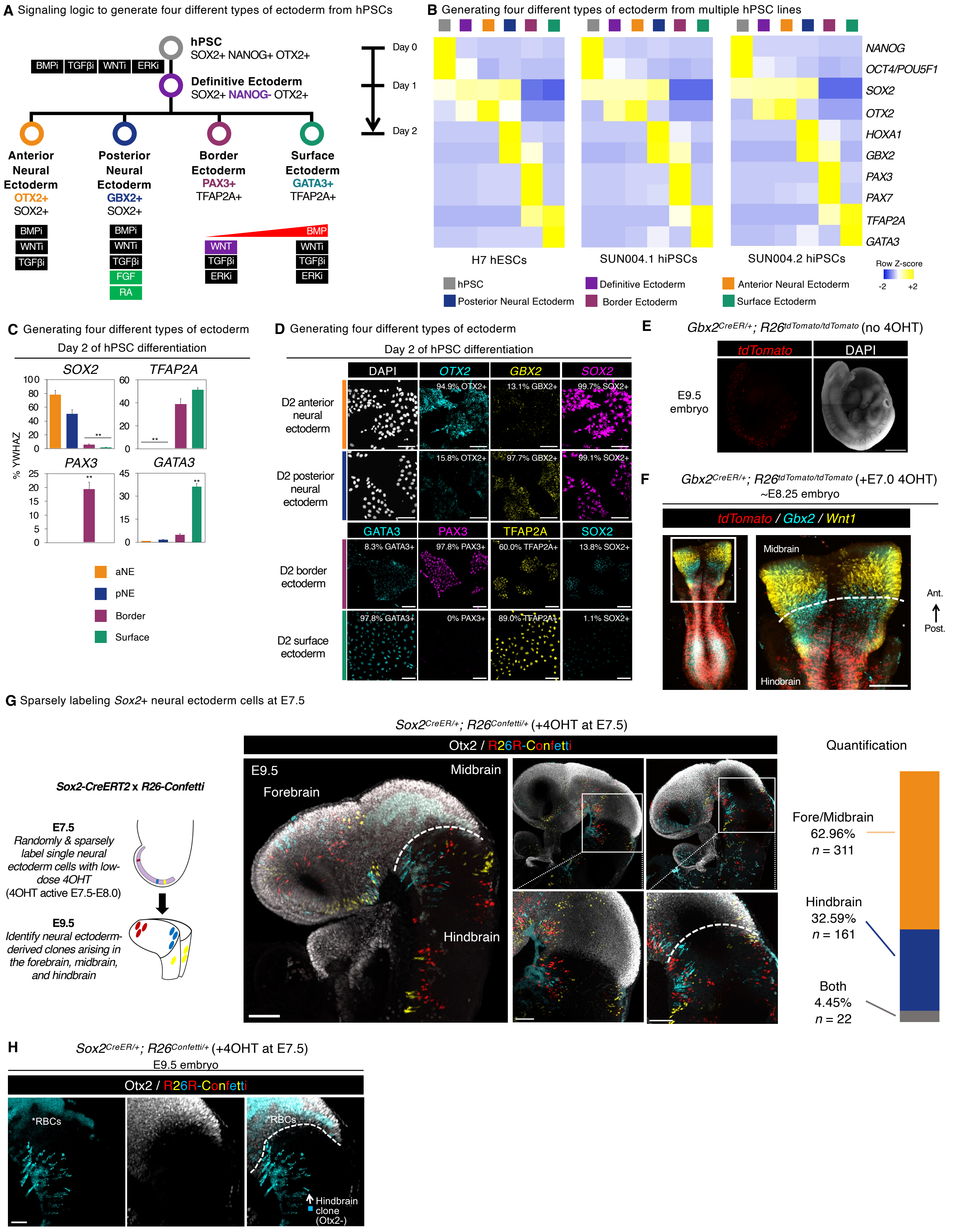
A Signaling logic to generate four different types of ectoderm from hPSCs
hPSC
SOX2+ NANOG+ OTX2+
BMPi TGFβi WNTi ERKi
Definitive Ectoderm
SOX2+ NANOG- OTX2+
Anterior Neural Ectoderm
OTX2+
SOX2+
Posterior Neural Ectoderm
GBX2+
SOX2+
Border Ectoderm
PAX3+
TFAP2A+
Surface Ectoderm
GATA3+
TFAP2A+
BMPi
WNTi
TGFβi
FGF
RA
BMP
WNT
ERKi
Day 0
Day 1
Day 2
B Generating four different types of ectoderm from multiple hPSC lines
NANOG
OCT4/POU5F1
SOX2
OTX2
HOXA1
GBX2
PAX3
PAX7
TFAP2A
GATA3
H7 hESCs
SUN004.1 hiPSCs
SUN004.2 hiPSCs
Row Z-score
-2
+2
hPSC
Definitive Ectoderm
Anterior Neural Ectoderm
Posterior Neural Ectoderm
Border Ectoderm
Surface Ectoderm
C Generating four different types of ectoderm
Day 2 of hPSC differentiation
SOX2
TFAP2A
PAX3
GATA3
% YWHAZ
aNE
pNE
Border
Surface
D Generating four different types of ectoderm
Day 2 of hPSC differentiation
DAPI
OTX2
GBX2
SOX2
D2 anterior neural ectoderm
94.9% OTX2+
13.1% GBX2+
99.7% SOX2+
D2 posterior neural ectoderm
15.8% OTX2+
97.7% GBX2+
99.1% SOX2+
GATA3
PAX3
TFAP2A
SOX2
D2 border ectoderm
8.3% GATA3+
97.8% PAX3+
60.0% TFAP2A+
13.8% SOX2+
D2 surface ectoderm
97.8% GATA3+
0% PAX3+
89.0% TFAP2A+
1.1% SOX2+
E Gbx2CreER/+; R26tdTomato/tdTomato (no 4OHT)
tdTomato
DAPI
E9.5 embryo
F Gbx2CreER/+; R26tdTomato/tdTomato (+E7.0 4OHT)
~E8.25 embryo
tdTomato / Gbx2 / Wnt1
Midbrain
Hindbrain
Ant.
Post.
G Sparsely labeling Sox2+ neural ectoderm cells at E7.5
Sox2CreER/+; R26Confetti/+ (+4OHT at E7.5)
Otx2 / R26R-Confetti
Sox2-CreERT2 x R26-Confetti
E7.5
Randomly & sparsely label single neural ectoderm cells with low-dose 4OHT
(4OHT active E7.5-E8.0)
E9.5
Identify neural ectoderm-derived clones arising in the forebrain, midbrain, and hindbrain
E9.5
Forebrain
Midbrain
Hindbrain
Quantification
Fore/Midbrain
62.96%
n = 311
Hindbrain
32.59%
n = 161
Both
4.45%
n = 22
H Sox2CreER/+; R26Confetti/+ (+4OHT at E7.5)
E9.5 embryo
Otx2 / R26R-Confetti
*RBCs
Hindbrain clone (Otx2-)

Figure S5

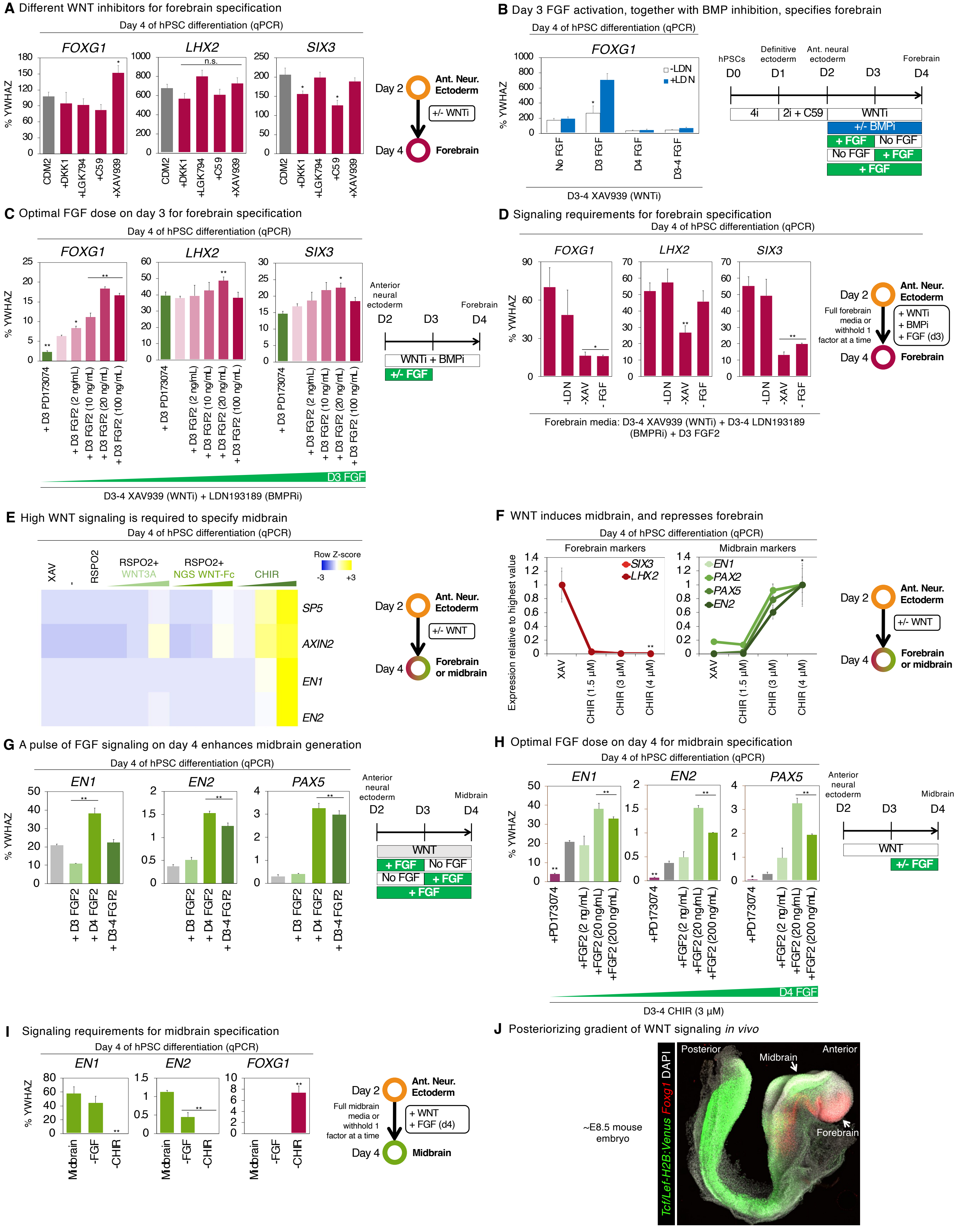
A Different WNT inhibitors for forebrain specification
Day 4 of hPSC differentiation (qPCR)
FOXG1
LHX2
SIX3
% YWHAZ
n.s.
CDM2
+DKK1
+LGK794
+C59
+XAV939
Day 2
Ant. Neur. Ectoderm
+/- WNTi
Day 4
Forebrain
B Day 3 FGF activation, together with BMP inhibition, specifies forebrain
Day 4 of hPSC differentiation (qPCR)
FOXG1
-LDN
+LDN
No FGF
D3 FGF
D4 FGF
D3-4 FGF
D3-4 XAV939 (WNTi)
hPSCs
Definitive ectoderm
Ant. neural ectoderm
Forebrain
D0
D1
D2
D3
D4
4i
2i + C59
WNTi
+/- BMPi
+ FGF
No FGF
No FGF
+ FGF
+ FGF
C Optimal FGF dose on day 3 for forebrain specification
Day 4 of hPSC differentiation (qPCR)
+ D3 PD173074
+ D3 FGF2 (2 ng/mL)
+ D3 FGF2 (10 ng/mL)
+ D3 FGF2 (20 ng/mL)
+ D3 FGF2 (100 ng/mL)
D3 FGF
D3-4 XAV939 (WNTi) + LDN193189 (BMPRi)
Anterior neural ectoderm
Forebrain
WNTi + BMPi
+/- FGF
D Signaling requirements for forebrain specification
Day 4 of hPSC differentiation (qPCR)
-LDN
-XAV
- FGF
Forebrain media: D3-4 XAV939 (WNTi) + D3-4 LDN193189 (BMPRi) + D3 FGF2
Full forebrain media or withhold 1 factor at a time
+ WNTi
+ BMPi
+ FGF (d3)
E High WNT signaling is required to specify midbrain
Day 4 of hPSC differentiation (qPCR)
XAV
-
RSPO2
RSPO2+ WNT3A
RSPO2+ NGS WNT-Fc
CHIR
Row Z-score
-3
+3
SP5
AXIN2
EN1
EN2
+/- WNT
Forebrain or midbrain
F WNT induces midbrain, and represses forebrain
Day 4 of hPSC differentiation (qPCR)
Forebrain markers
Midbrain markers
Expression relative to highest value
SIX3
LHX2
EN1
PAX2
PAX5
EN2
XAV
CHIR (1.5 μM)
CHIR (3 μM)
CHIR (4 μM)
G A pulse of FGF signaling on day 4 enhances midbrain generation
Day 4 of hPSC differentiation (qPCR)
EN1
EN2
PAX5
+ D3 FGF2
+ D4 FGF2
+ D3-4 FGF2
Anterior neural ectoderm
Midbrain
WNT
+ FGF
No FGF
No FGF
+ FGF
+ FGF
H Optimal FGF dose on day 4 for midbrain specification
Day 4 of hPSC differentiation (qPCR)
+PD173074
+FGF2 (2 ng/mL)
+FGF2 (20 ng/mL)
+FGF2 (200 ng/mL)
D4 FGF
D3-4 CHIR (3 μM)
+/- FGF
I Signaling requirements for midbrain specification
Day 4 of hPSC differentiation (qPCR)
EN1
EN2
FOXG1
Midbrain
-FGF
-CHIR
Full midbrain media or withhold 1 factor at a time
+ WNT
+ FGF (d4)
Midbrain
J Posteriorizing gradient of WNT signaling in vivo
~E8.5 mouse embryo
Tcf/Lef-H2B:Venus Foxg1 DAPI
Posterior
Anterior
Midbrain
Forebrain

Figure S6

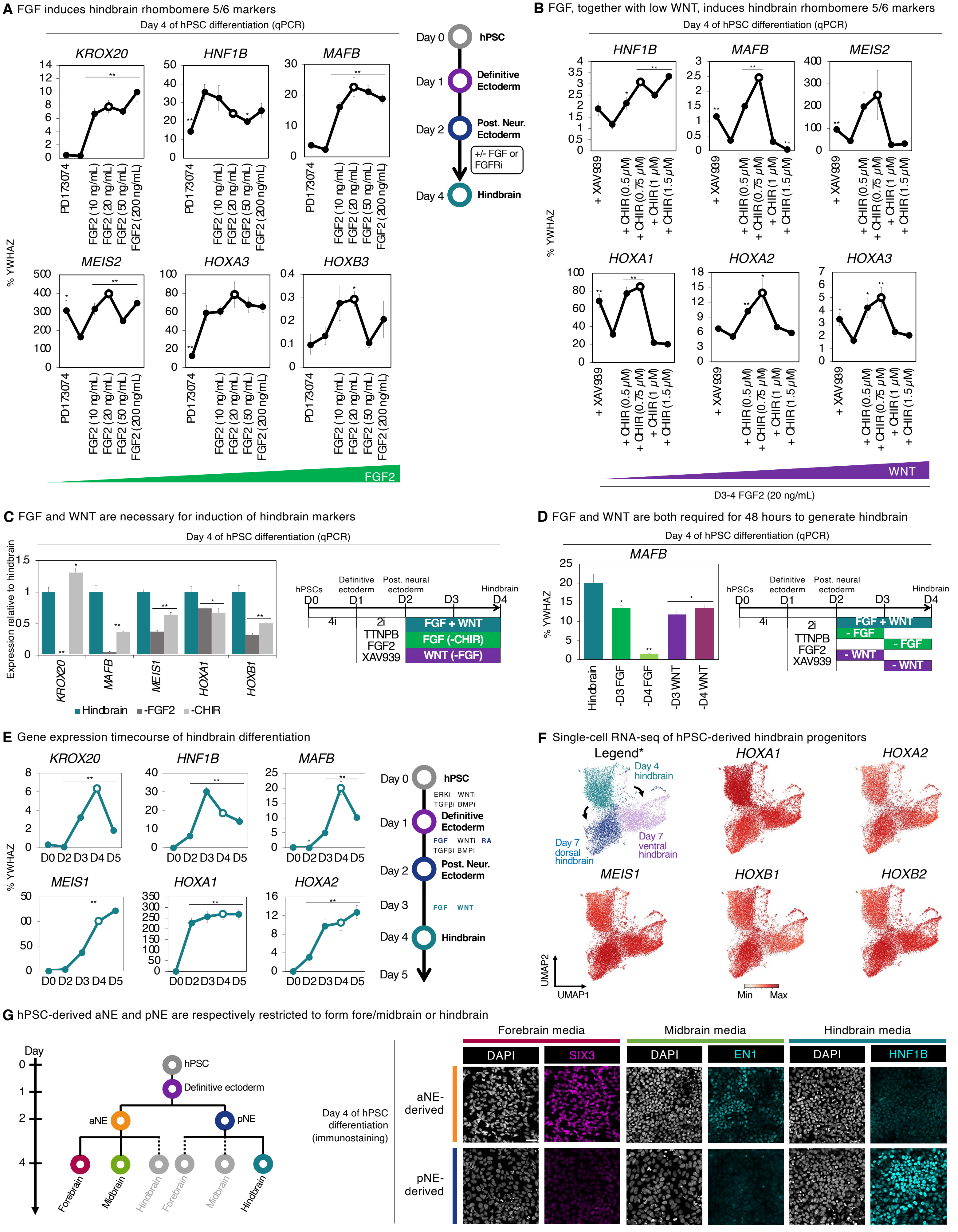

A FGF induces hindbrain rhombomere 5/6 markers
Day 4 of hPSC differentiation (qPCR)
KROX20
HNF1B
MAFB
MEIS2
HOXA3
HOXB3
% YWHAZ
PD173074
FGF2 (10 ng/mL)
FGF2 (20 ng/mL)
FGF2 (50 ng/mL)
FGF2 (200 ng/mL)
FGF2
Day 0
hPSC
Day 1
Definitive Ectoderm
Day 2
Post. Neur. Ectoderm
+/- FGF or FGFRi
Day 4
Hindbrain
B FGF, together with low WNT, induces hindbrain rhombomere 5/6 markers
Day 4 of hPSC differentiation (qPCR)
HNF1B
MAFB
MEIS2
HOXA1
HOXA2
HOXA3
% YWHAZ
+ XAV939
+ CHIR (0.5 μM)
+ CHIR (0.75 μM)
+ CHIR (1 μM)
+ CHIR (1.5 μM)
WNT
D3-4 FGF2 (20 ng/mL)
C FGF and WNT are necessary for induction of hindbrain markers
Day 4 of hPSC differentiation (qPCR)
Expression relative to hindbrain
KROX20
MAFB
MEIS1
HOXA1
HOXB1
Hindbrain
-FGF2
-CHIR
hPSCs
Definitive ectoderm
Post. neural ectoderm
Hindbrain
D0
D1
D2
D3
D4
4i
2i
TTNPB
FGF2
XAV939
FGF + WNT
FGF (-CHIR)
WNT (-FGF)
D FGF and WNT are both required for 48 hours to generate hindbrain
Day 4 of hPSC differentiation (qPCR)
MAFB
% YWHAZ
Hindbrain
-D3 FGF
-D4 FGF
-D3 WNT
-D4 WNT
- FGF
- WNT
E Gene expression timecourse of hindbrain differentiation
KROX20
HNF1B
MAFB
MEIS1
HOXA1
HOXA2
% YWHAZ
D0 D2 D3 D4 D5
Day 0
hPSC
ERKi WNTi
TGFβi BMPi
Day 1
Definitive Ectoderm
FGF WNTi RA
TGFβi BMPi
Day 2
Post. Neur. Ectoderm
Day 3
FGF WNT
Day 4
Hindbrain
Day 5
F Single-cell RNA-seq of hPSC-derived hindbrain progenitors
Legend*
Day 4 hindbrain
Day 7 dorsal hindbrain
Day 7 ventral hindbrain
HOXA1
HOXA2
MEIS1
HOXB1
HOXB2
UMAP1
UMAP2
Min
Max
G hPSC-derived aNE and pNE are respectively restricted to form fore/midbrain or hindbrain
Day
hPSC
Definitive ectoderm
aNE
pNE
Forebrain
Midbrain
Hindbrain
Day 4 of hPSC differentiation (immunostaining)
Forebrain media
Midbrain media
Hindbrain media
DAPI
SIX3
EN1
HNF1B
aNE-derived
pNE-derived

Figure S7

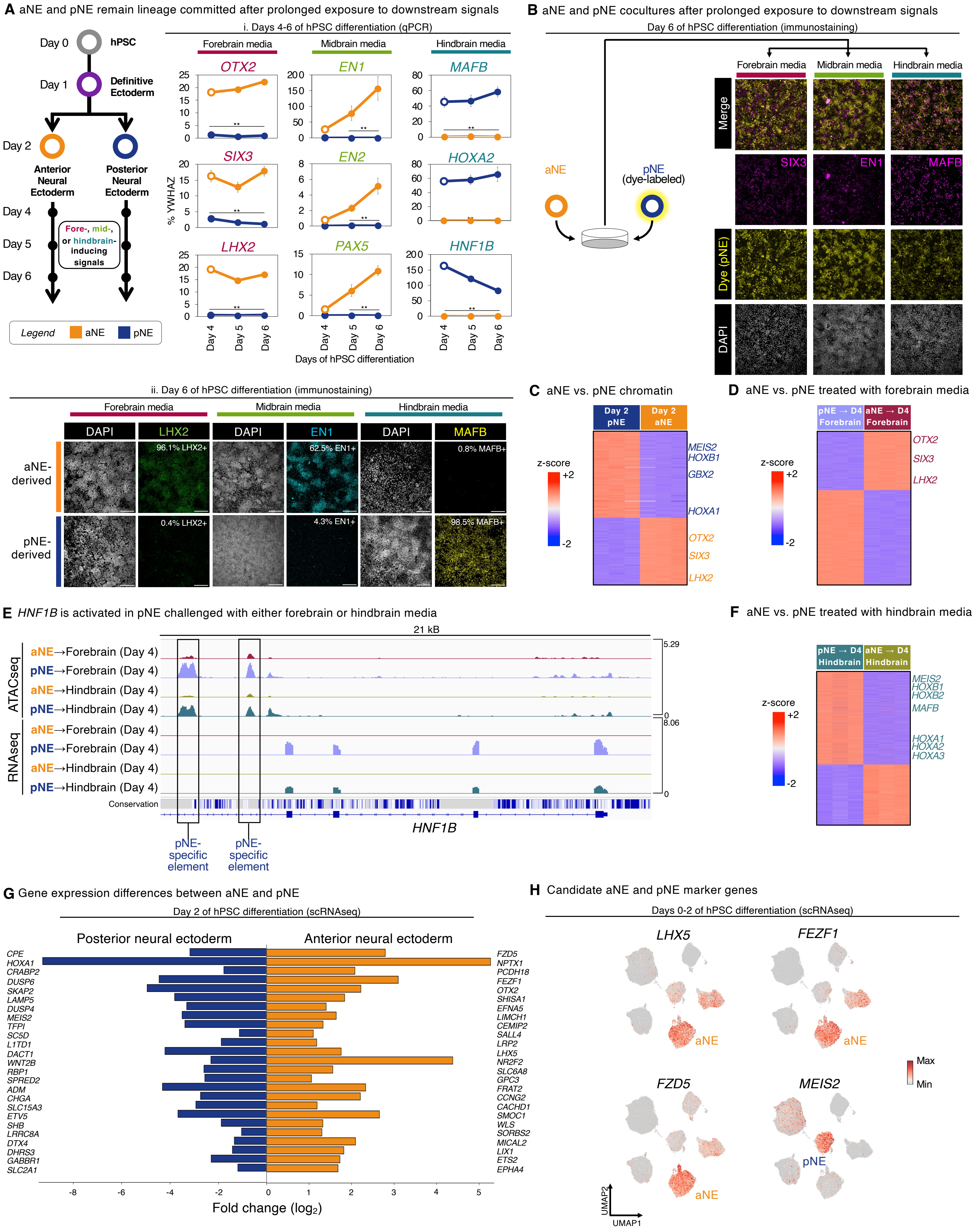

A aNE and pNE remain lineage committed after prolonged exposure to downstream signals
Day 0
hPSC
Day 1
Definitive Ectoderm
Day 2
Anterior Neural Ectoderm
Posterior Neural Ectoderm
Day 4
Day 5
Day 6
Fore-, mid-, or hindbrain-inducing signals
Legend
aNE
pNE
i. Days 4-6 of hPSC differentiation (qPCR)
Forebrain media
Midbrain media
Hindbrain media
OTX2
EN1
MAFB
SIX3
EN2
HOXA2
LHX2
PAX5
HNF1B
% YWHAZ
Days of hPSC differentiation
B aNE and pNE cocultures after prolonged exposure to downstream signals
Day 6 of hPSC differentiation (immunostaining)
aNE
pNE (dye-labeled)
Merge
SIX3
EN1
MAFB
Dye (pNE)
DAPI
ii. Day 6 of hPSC differentiation (immunostaining)
aNE-derived
pNE-derived
96.1% LHX2+
62.5% EN1+
0.8% MAFB+
0.4% LHX2+
4.3% EN1+
98.5% MAFB+
C aNE vs. pNE chromatin
Day 2 pNE
Day 2 aNE
z-score
+2
-2
MEIS2
HOXB1
GBX2
HOXA1
OTX2
SIX3
LHX2
D aNE vs. pNE treated with forebrain media
pNE → D4 Forebrain
aNE → D4 Forebrain
E HNF1B is activated in pNE challenged with either forebrain or hindbrain media
21 kB
ATACseq
RNAseq
aNE→Forebrain (Day 4)
pNE→Forebrain (Day 4)
aNE→Hindbrain (Day 4)
pNE→Hindbrain (Day 4)
5.29
8.06
0
Conservation
HNF1B
pNE-specific element
pNE-specific element
F aNE vs. pNE treated with hindbrain media
pNE → D4 Hindbrain
aNE → D4 Hindbrain
MEIS2
HOXB1
HOXB2
MAFB
HOXA1
HOXA2
HOXA3
G Gene expression differences between aNE and pNE
Day 2 of hPSC differentiation (scRNAseq)
Posterior neural ectoderm
Anterior neural ectoderm
CPE
HOXA1
CRABP2
DUSP6
SKAP2
LAMP5
DUSP4
MEIS2
TFPI
SC5D
L1TD1
DACT1
WNT2B
RBP1
SPRED2
ADM
CHGA
SLC15A3
ETV5
SHB
LRRC8A
DTX4
DHRS3
GABBR1
SLC2A1
FZD5
NPTX1
PCDH18
FEZF1
OTX2
SHISA1
EFNA5
LIMCH1
CEMIP2
SALL4
LRP2
LHX5
NR2F2
SLC6A8
GPC3
FRAT2
CCNG2
CACHD1
SMOC1
WLS
SORBS2
MICAL2
LIX1
ETS2
EPHA4
-8
-6
-4
-2
0
1
2
3
4
5
Fold change (log2)
H Candidate aNE and pNE marker genes
Days 0-2 of hPSC differentiation (scRNAseq)
LHX5
FEZF1
FZD5
MEIS2
aNE
pNE
Max
Min
UMAP1
UMAP2

Figure S8

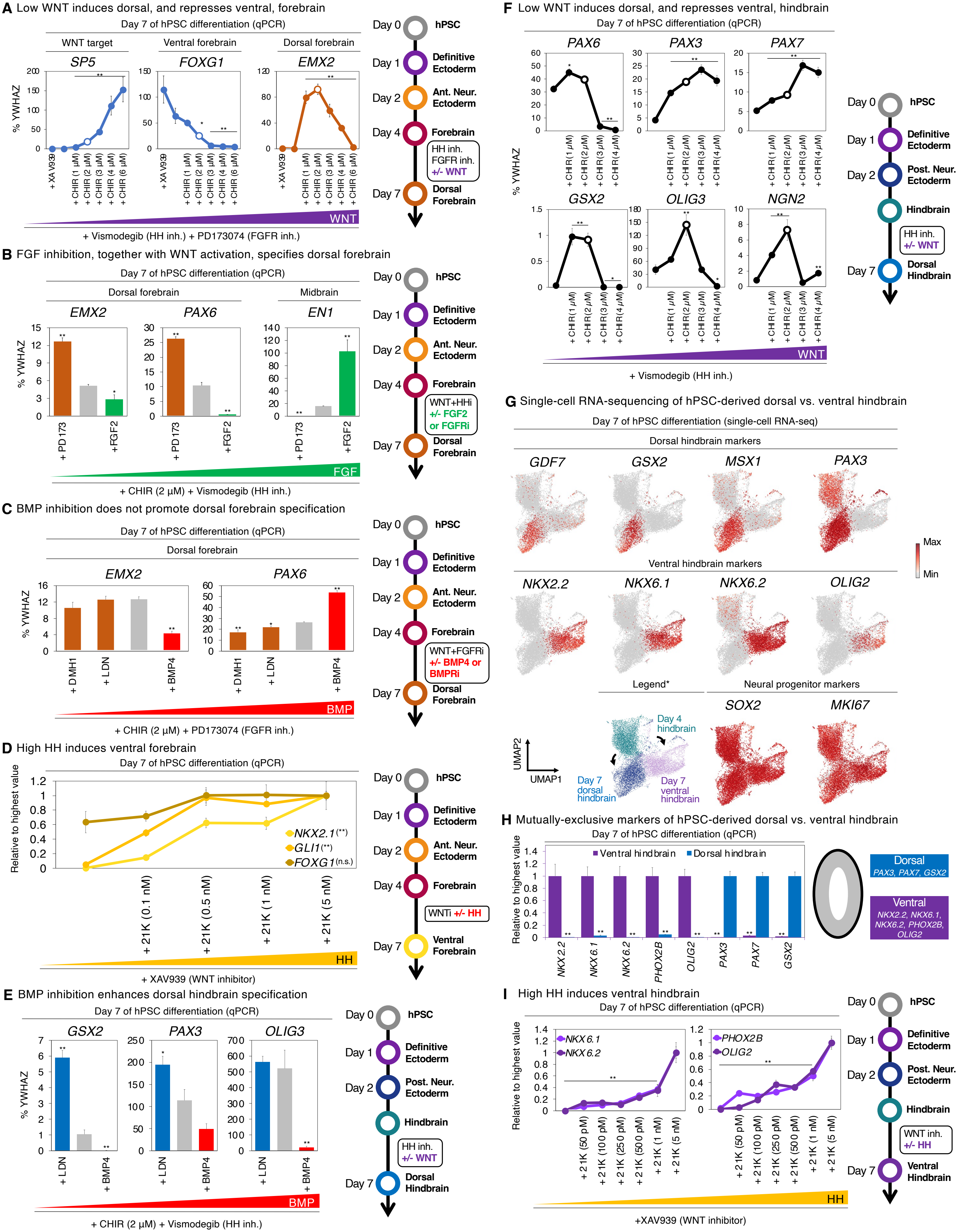
A Low WNT induces dorsal, and represses ventral, forebrain
Day 7 of hPSC differentiation (qPCR)
WNT target
Ventral forebrain
Dorsal forebrain
SP5
FOXG1
EMX2
% YWHAZ
+ XAV939
+ CHIR (1 µM)
+ CHIR (2 µM)
+ CHIR (3 µM)
+ CHIR (4 µM)
+ CHIR (6 µM)
WNT
+ Vismodegib (HH inh.) + PD173074 (FGFR inh.)
Day 0 hPSC
Day 1 Definitive Ectoderm
Day 2 Ant. Neur. Ectoderm
Day 4 Forebrain
HH inh. FGFR inh. +/- WNT
Day 7 Dorsal Forebrain
B FGF inhibition, together with WNT activation, specifies dorsal forebrain
Day 7 of hPSC differentiation (qPCR)
Dorsal forebrain
Midbrain
EMX2
PAX6
EN1
% YWHAZ
+ PD173
+ FGF2
FGF
+ CHIR (2 µM) + Vismodegib (HH inh.)
WNT+HHi +/- FGF2 or FGFRi
C BMP inhibition does not promote dorsal forebrain specification
Day 7 of hPSC differentiation (qPCR)
Dorsal forebrain
EMX2
PAX6
% YWHAZ
+ DMH1
+ LDN
+ BMP4
BMP
+ CHIR (2 µM) + PD173074 (FGFR inh.)
WNT+FGFRi +/- BMP4 or BMPRi
D High HH induces ventral forebrain
Day 7 of hPSC differentiation (qPCR)
Relative to highest value
NKX2.1(**)
GLI1(**)
FOXG1(n.s.)
+ 21K (0.1 nM)
+ 21K (0.5 nM)
+ 21K (1 nM)
+ 21K (5 nM)
HH
+ XAV939 (WNT inhibitor)
WNTi +/- HH
Day 7 Ventral Forebrain
E BMP inhibition enhances dorsal hindbrain specification
Day 7 of hPSC differentiation (qPCR)
GSX2
PAX3
OLIG3
% YWHAZ
+ LDN
+ BMP4
BMP
+ CHIR (2 µM) + Vismodegib (HH inh.)
Day 2 Post. Neur. Ectoderm
Hindbrain
HH inh. +/- WNT
Day 7 Dorsal Hindbrain
F Low WNT induces dorsal, and represses ventral, hindbrain
Day 7 of hPSC differentiation (qPCR)
PAX6
PAX3
PAX7
GSX2
OLIG3
NGN2
% YWHAZ
+ CHIR (1 µM)
+ CHIR (2 µM)
+ CHIR (3 µM)
+ CHIR (4 µM)
WNT
+ Vismodegib (HH inh.)
G Single-cell RNA-sequencing of hPSC-derived dorsal vs. ventral hindbrain
Day 7 of hPSC differentiation (single-cell RNA-seq)
Dorsal hindbrain markers
GDF7
GSX2
MSX1
PAX3
Max
Min
Ventral hindbrain markers
NKX2.2
NKX6.1
NKX6.2
OLIG2
Legend*
Neural progenitor markers
SOX2
MKI67
UMAP2
UMAP1
Day 4 hindbrain
Day 7 dorsal hindbrain
Day 7 ventral hindbrain
H Mutually-exclusive markers of hPSC-derived dorsal vs. ventral hindbrain
Day 7 of hPSC differentiation (qPCR)
Relative to highest value
Ventral hindbrain
Dorsal hindbrain
NKX2.2
NKX6.1
NKX6.2
PHOX2B
OLIG2
PAX3
PAX7
GSX2
Dorsal
PAX3, PAX7, GSX2
Ventral
NKX2.2, NKX6.1, NKX6.2, PHOX2B, OLIG2
I High HH induces ventral hindbrain
Day 7 of hPSC differentiation (qPCR)
Relative to highest value
NKX6.1
NKX6.2
PHOX2B
OLIG2
+ 21K (50 pM)
+ 21K (100 pM)
+ 21K (250 pM)
+ 21K (500 pM)
+ 21K (1 nM)
+ 21K (5 nM)
HH
+XAV939 (WNT inhibitor)
WNT inh. +/- HH
Day 7 Ventral Hindbrain

Figure S9

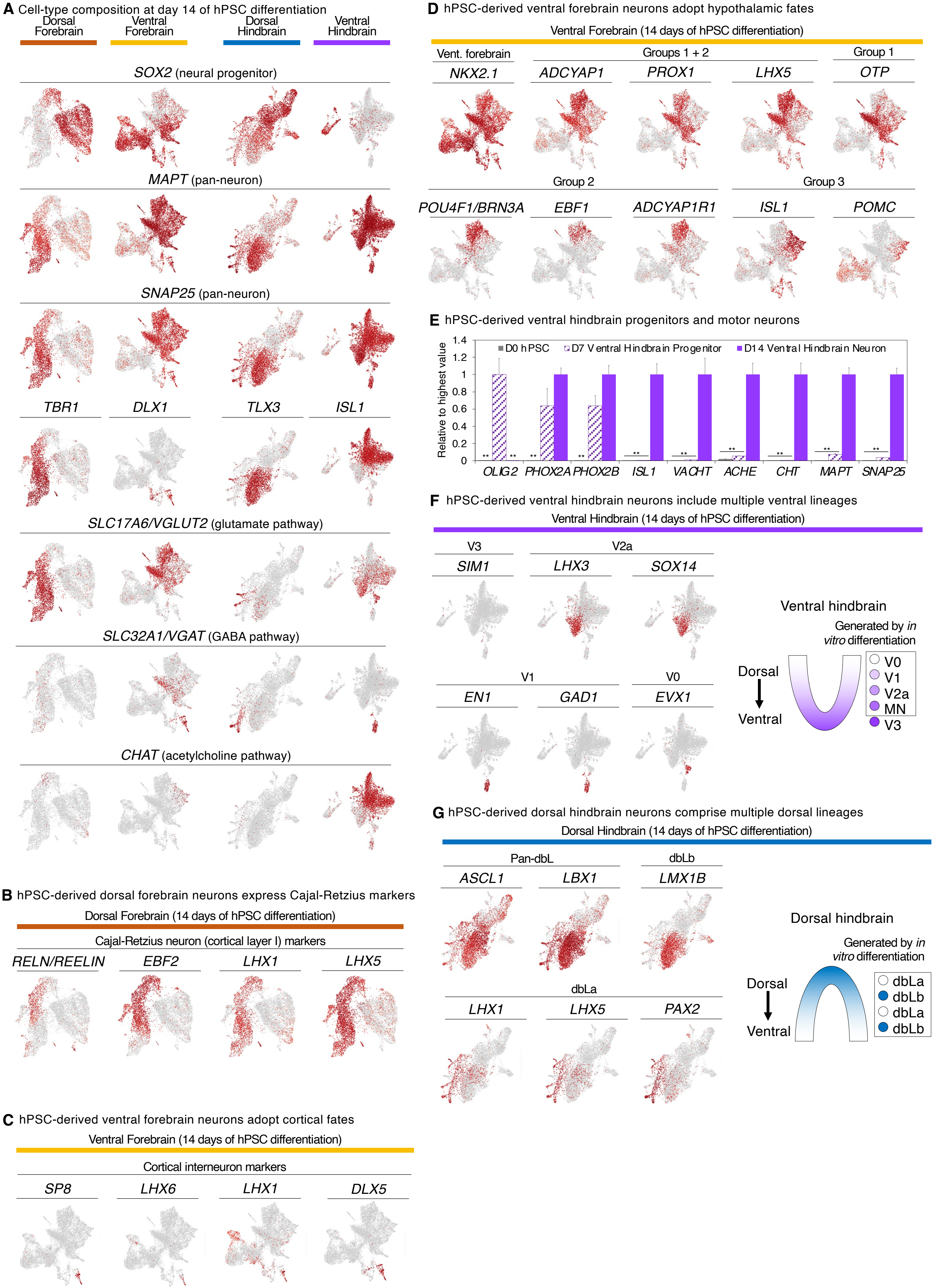
A Cell-type composition at day 14 of hPSC differentiation
Dorsal Forebrain
Ventral Forebrain
Dorsal Hindbrain
Ventral Hindbrain
SOX2 (neural progenitor)
MAPT (pan-neuron)
SNAP25 (pan-neuron)
TBR1
DLX1
TLX3
ISL1
SLC17A6/VGLUT2 (glutamate pathway)
SLC32A1/VGAT (GABA pathway)
CHAT (acetylcholine pathway)
B hPSC-derived dorsal forebrain neurons express Cajal-Retzius markers
Dorsal Forebrain (14 days of hPSC differentiation)
Cajal-Retzius neuron (cortical layer I) markers
RELN/REELIN
EBF2
LHX1
LHX5
C hPSC-derived ventral forebrain neurons adopt cortical fates
Ventral Forebrain (14 days of hPSC differentiation)
Cortical interneuron markers
SP8
LHX6
LHX1
DLX5
D hPSC-derived ventral forebrain neurons adopt hypothalamic fates
Ventral Forebrain (14 days of hPSC differentiation)
Vent. forebrain
Groups 1 + 2
Group 1
NKX2.1
ADCYAP1
PROX1
LHX5
OTP
Group 2
Group 3
POU4F1/BRN3A
EBF1
ADCYAP1R1
ISL1
POMC
E hPSC-derived ventral hindbrain progenitors and motor neurons
Relative to highest value
1.4
1.2
1
0.8
0.6
0.4
0.2
0
D0 hPSC
D7 Ventral Hindbrain Progenitor
D14 Ventral Hindbrain Neuron
**
OLIG2
PHOX2A
PHOX2B
ISL1
VACHT
ACHE
CHT
MAPT
SNAP25
F hPSC-derived ventral hindbrain neurons include multiple ventral lineages
Ventral Hindbrain (14 days of hPSC differentiation)
V3
V2a
SIM1
LHX3
SOX14
V1
V0
EN1
GAD1
EVX1
Ventral hindbrain
Generated by in vitro differentiation
Dorsal
Ventral
V0
V1
V2a
MN
V3
G hPSC-derived dorsal hindbrain neurons comprise multiple dorsal lineages
Dorsal Hindbrain (14 days of hPSC differentiation)
Pan-dbL
dbLb
ASCL1
LBX1
LMX1B
dbLa
LHX1
LHX5
PAX2
Dorsal hindbrain
Generated by in vitro differentiation
Dorsal
Ventral
dbLa
dbLb
dbLa
dbLb

Figure S10

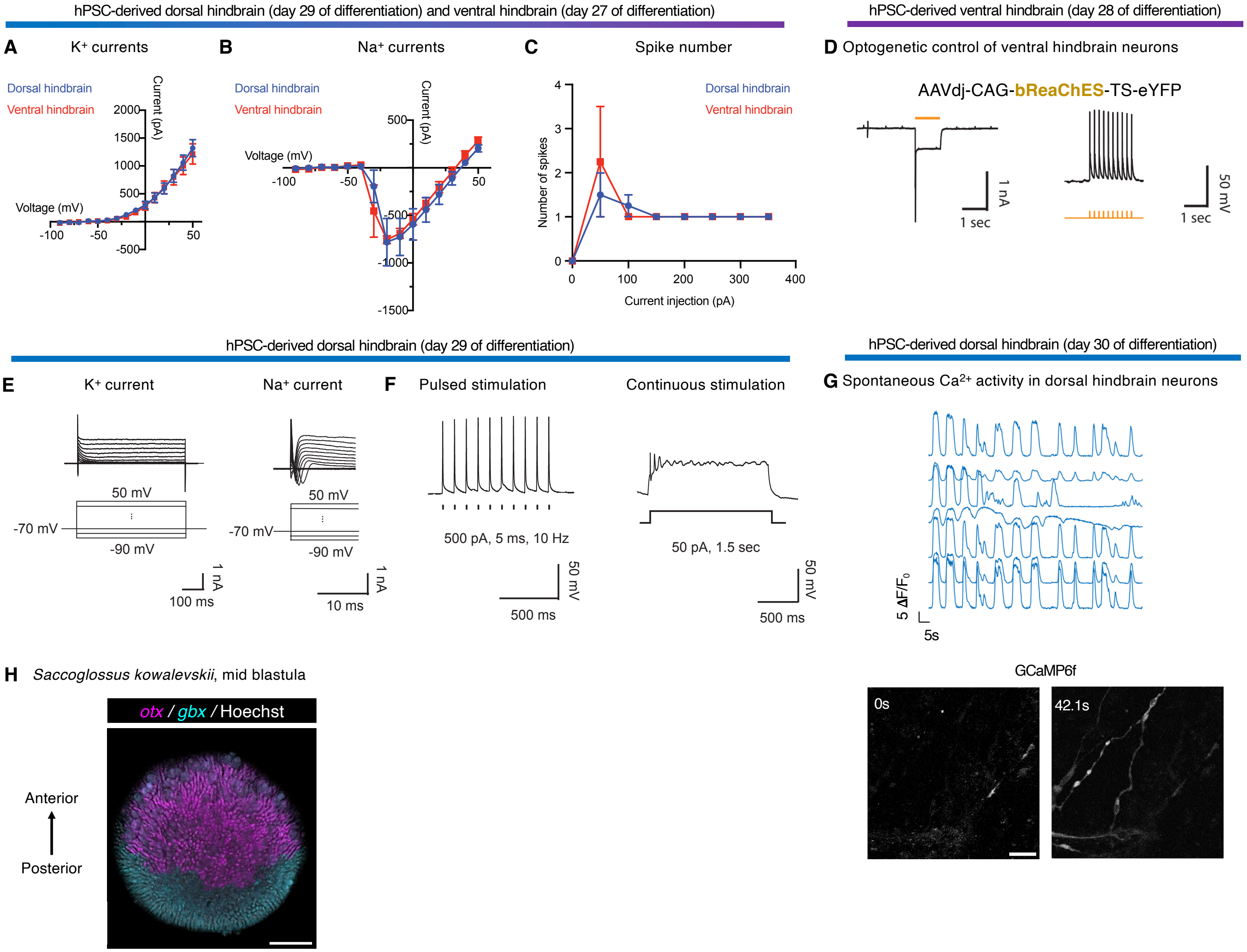

hPSC-derived dorsal hindbrain (day 29 of differentiation) and ventral hindbrain (day 27 of differentiation)
hPSC-derived ventral hindbrain (day 28 of differentiation)
A K+ currents
Dorsal hindbrain
Ventral hindbrain
Current (pA)
Voltage (mV)
B Na+ currents
C Spike number
Number of spikes
Current injection (pA)
D Optogenetic control of ventral hindbrain neurons
AAVdj-CAG-bReaChES-TS-eYFP
1 nA
1 sec
50 mV
hPSC-derived dorsal hindbrain (day 29 of differentiation)
hPSC-derived dorsal hindbrain (day 30 of differentiation)
E K+ current
Na+ current
50 mV
-70 mV
-90 mV
100 ms
10 ms
F Pulsed stimulation
Continuous stimulation
500 pA, 5 ms, 10 Hz
50 pA, 1.5 sec
500 ms
G Spontaneous Ca2+ activity in dorsal hindbrain neurons
5 ΔF/F0
5s
GCaMP6f
0s
42.1s
H Saccoglossus kowalevskii, mid blastula
otx / gbx / Hoechst
Anterior
Posterior

Supplementary Figure Legends

Supplementary Figure 1: Differentiation of hPSCs into definitive ectoderm within 24 hours, and *in vivo* analysis of anterior and posterior neural ectoderm

A) Single-cell RNA-sequencing of E6.75 mouse embryos; analysis performed on a previously-published resource [169]. Embryonic cells are shown, with extraembryonic cells excluded (*Elf5* and *Rhox5* $\log_2$ normalized expression = 0). *Otx2* is expressed in definitive ectoderm and pluripotent cells, consistent with previous studies [16,79]. *Oct4* (*Pou5f1*) is broadly expressed across all cell-types, consistent with past studies [70,71].

B) qPCR of H7 hPSCs differentiated for 24 hours with "dual SMAD inhibitors" (TGFβ inhibitor A-83-01 [1 μM] and BMP inhibitor LDN-193189 [250 nM]), in the presence or absence of WNT inhibitor (C59, 1 μM) and ERK inhibitor (Trametinib, 250 nM). Gene expression is shown relative to reference gene *YWHAZ*; 100% indicates that a gene is expressed at the same level as *YWHAZ*. "D0" = day-0 undifferentiated hPSCs. i: inhibitor.

C) Immunostaining of hPSCs prior to, or after, differentiation into definitive ectoderm for 24 hours. NANOG and SOX2 immunostaining was performed with H7 hPSCs. OTX2 immunostaining was performed with H1 hPSCs. Scale bar = 100 μm.

D) qPCR of H7 hPSCs differentiated for 24 hours with "dual SMAD inhibitors" (TGFβ inhibitor A-83-01 [1 μM] and BMP inhibitor LDN-193189 [250 nM]), in the presence or absence of individual ERK inhibitors (PD0325901, Trametinib, AZD6244, GDC0623 or Pimasertib) or FGFR inhibitors (AZD4547, PD173074 or BGJ398). Gene expression is shown relative to reference gene *YWHAZ*; 100% indicates that a gene is expressed at the same level as *YWHAZ*. D0: day-0 undifferentiated hPSCs. i: inhibitor.

E) Flow cytometry of H1 hPSCs differentiated into definitive ectoderm for 24 hours. The percentage of SOX2+ NANOG- definitive ectoderm cells is shown. Individual datapoints depict the outcomes of independent experiments.

F) qPCR of H1, H7, SUN004.1 and SUN004.2 hPSCs prior to, or after, differentiation into definitive ectoderm with BMP, ERK, TGFβ and WNT inhibitors (LDN-193189, Trametinib, A-83-01 and C59, respectively) for 24 hours. Gene expression is generally shown relative to the sample with the highest expression in each cell line.

G) qPCR of H1 hPSCs seeded at different densities, and then differentiated into definitive ectoderm with BMP, ERK, TGFβ and WNT inhibitors (LDN-193189, Trametinib, A-83-01 and C59, respectively) for 24 hours. qPCR data normalized to undifferentiated hPSCs. Open circle indicates a seeding density of 520 cells/mm^2, which was the baseline used throughout this study. Gene expression is shown relative to reference gene *YWHAZ*; 100% indicates that a gene is expressed at the same level as *YWHAZ*.

H) qPCR of H1 hPSCs differentiated for 24 hours into definitive ectoderm with BMP, ERK, TGFβ and WNT inhibitors (LDN-193189, Trametinib, A-83-01 and C59, respectively), or with each ectoderm-inducing signal individually withheld. Gene expression is shown relative to reference gene *YWHAZ*; 100% indicates that a gene is expressed at the same level as *YWHAZ*.

I) qPCR of H1 hPSCs that were either differentiated into anterior primitive streak [78] or definitive ectoderm for 24 hours, followed by treatment with either endoderm-inducing media [77] (Activin A [100 ng/mL] + LDN193189 [250 nM]) for 24 hours, or paraxial mesoderm-inducing media[78] (A-83-01 [1 μM] + LDN193189 [250 nM] + CHIR99021 [3

μM] + FGF2 [20 ng/mL]) for 24 hours. This revealed that hPSC-derived ectoderm largely failed to express either endoderm or paraxial mesoderm markers. By contrast, hPSC-derived primitive streak exposed to the same signals turned on either endoderm or paraxial mesoderm markers. Gene expression is shown relative to reference gene *YWHAZ*; 100% indicates that a gene is expressed at the same level as *YWHAZ*.

J) Flow cytometry of HES3 *MIXL1-GFP* hPSCs differentiated into definitive ectoderm (day 1), anterior neural ectoderm (day 2), posterior neural ectoderm (day 2), border ectoderm (day 2), surface ectoderm (day 1), or mid primitive streak [78] (day 1, as a positive control to induce MIXL1 expression). Negligible numbers of MIXL1+ primitive streak cells emerged after ectoderm differentiation, reaffirming the suppression of spurious primitive streak differentiation.

K) *In situ* staining of E7.5 mouse embryo, showing overlap of *Sox2* and *Gbx2* expression in pNE. Scale bar = 25 μm.

L) Single-cell RNA-sequencing of E7.5 mouse embryos; analysis performed on a previously-published resource[169]. Neural ectoderm cells (identified by $\log_2$ normalized expression of *Sox2* > 0.5, and *Nanog, Tfap2a, Rhox5, Foxj1,* and *Elf5* = 0) are shown.

M) Intracellular flow cytometry for SOX2 protein expression in H1 hPSCs, prior to or after differentiation into anterior neural ectoderm or posterior neural ectoderm for 2 days. The percentage of SOX2+ cells, and median fluorescence intensity (MFI) of SOX2 expression, are both shown.

N) Immunostaining of H1 hPSCs differentiated into definitive ectoderm for 24 hours, followed by differentiation into aNE or pNE for 24 hours. Scale bar = 100 μm.

qPCR data depicts the mean of two biological replicates, with s.e.m. shown. For flow cytometry and *in vitro* staining, a representative image from two biological replicates is shown.

Supplementary Figure 2: Differentiation of hPSCs into anterior or posterior neural ectoderm within 48 hours, and *in vivo* analysis of signaling fields

A) qPCR of H1 hPSCs differentiated into definitive ectoderm for 24 hours, followed by BMP, TGFβ and WNT inhibitors (LDN-193189, A-83-01 and C59, respectively) for 24 hours to generate anterior neural ectoderm. To assess the importance of each anterior neural ectoderm-inducing signal, each factor was individually withheld. Gene expression is shown relative to reference gene *YWHAZ*; 100% indicates that a gene is expressed at the same level as *YWHAZ*. D2 aNE: day-2 anterior neural ectoderm.

B) qPCR of H1 hPSCs were differentiated into definitive ectoderm for 24 hours, followed by differentiation into posterior neural ectoderm for 24 hours. During posterior neural ectoderm induction, different doses of FGF agonist (FGF2) or RA agonist (TTNPB) were added to assess dose-dependent effects. Open circles indicate the optimal doses of FGF2 (20 ng/mL) and TTNPB (50 nM) for posterior neural ectoderm induction used as a baseline throughout this study. Gene expression is shown relative to reference gene *YWHAZ*; 100% indicates that a gene is expressed at the same level as *YWHAZ*.

C) qPCR of H1 hPSCs differentiated into definitive ectoderm for 24 hours, followed by a combination of FGF and RA agonists (FGF2 and TTNPB, respectively) together with BMP, TGFβ and WNT inhibitors (LDN-193189, A-83-01 and C59, respectively) for 24 hours to generate posterior neural ectoderm. To assess the importance of each posterior neural ectoderm-inducing signal, each factor was individually withheld. Gene

expression is shown relative to reference gene *YWHAZ*; 100% indicates that a gene is expressed at the same level as *YWHAZ*. "D2 pNE": day-2 posterior neural ectoderm.

D) Western Blot to detect the expression of GBX2 protein in H1 hPSC-derived day-2 aNE or day-2 pNE. VINCULIN protein serves as a loading control.

E) qPCR of H1 hPSCs differentiated into definitive ectoderm for 24 hours, followed by exposure to (1) neural-inducing signals (A-83-01 + LDN-193189), (2) neural-inducing signals (A-83-01 + LDN-193189) + FGF2 (20 ng/mL) + TTNPB (50 nM) or (3) neural-inducing signals (A-83-01 + LDN-193189) + CHIR99021 (2 μM) for 24 hours. Gene expression is shown relative to reference gene *YWHAZ*; 100% indicates that a gene is expressed at the same level as *YWHAZ*.

F) qPCR of H1 hPSCs were differentiated into definitive ectoderm for 24 hours, followed by differentiation into anterior neural ectoderm for 24 hours (with A-83-01, LDN-193189, and C59). Alternatively, to test the effect of WNT signaling on anterior neural ectoderm induction, WNT inhibitor (C59) was withheld, and WNT agonist (CHIR99021) was added instead. Gene expression is shown relative to reference gene *YWHAZ*; 100% indicates that a gene is expressed at the same level as *YWHAZ*.

G) qPCR of H1 hPSCs were differentiated into definitive ectoderm for 24 hours, followed by differentiation into posterior neural ectoderm for 24 hours (with A-83-01, LDN-193189, C59, FGF2 and TTNPB). Alternatively, to test the effect of WNT signaling on posterior neural ectoderm induction, WNT inhibitor (C59) was withheld, and WNT agonist (CHIR99021) was added instead. Gene expression is shown relative to reference gene *YWHAZ*; 100% indicates that a gene is expressed at the same level as *YWHAZ*.

H) First, qPCR was performed of H1 hPSCs that were differentiated into definitive ectoderm for 24 hours with A8301, LDN193189, Trametinib, and WNT inhibitor (either C59 [1 μM], XAV939 [1 μM], or DKK1 [300 ng/mL]), revealing that all three WNT inhibitors comparably induced definitive ectoderm (*left*). Second, qPCR was performed of H1 hPSCs that were differentiated into definitive ectoderm for 24 hours with A8301, LDN193189, Trametinib, and XAV939, followed by differentiation into posterior neural ectoderm for 24 hours with A8301, LDN193189, TTNPB, FGF2, and WNT inhibitor (either C59 [1 μM], XAV939 [1 μM], or DKK1 [300 ng/mL]), revealing that all three WNT inhibitors comparably induced posterior neural ectoderm (*right*). Given these comparable results, throughout the rest of this study we applied C59 for definitive ectoderm induction and XAV939 for posterior neural ectoderm induction, as indicated in bold in the accompanying cartoon. Gene expression is shown relative to reference gene *YWHAZ*; 100% indicates that a gene is expressed at the same level as *YWHAZ*.

I) β-CATENIN immunostaining of H1 hPSCs that were differentiated into various cell-types. First, hPSCs were differentiated into definitive ectoderm for 24 hours, and then treated with posterior neural ectoderm-inducing media for 24 hours (A8301 [1 μM] + LDN193189 [100 nM] + TTNPB [50 nM] + FGF2 [20 ng/mL] + XAV939 [1 μM] + Thiazovivin [1 μM]), or as a positive control to induce nuclear β-CATENIN, modified posterior neural ectoderm media lacking WNT inhibitor and including a WNT agonist for 24 hours (A8301 [1 μM] + LDN193189 [100 nM] + TTNPB [50 nM] + FGF2 [20 ng/mL] + CHIR99021 [2 μM] + Thiazovivin [1 μM]). Alternatively, as a positive control to induce nuclear β-CATENIN, hPSCs were differentiated into mid primitive streak, with WNT agonist-containing media (Activin A [30 ng/mL] + BMP4 [40 ng/mL] + CHIR99021 [6 μM]

+ FGF2 [20 ng/mL]) for 24 hours. Nuclear β-CATENIN was absent from hPSC-derived posterior neural ectoderm, and either weakly or strongly positive in the two WNT agonist-treated cell populations. Scale bar = 100 μm.

qPCR data depicts the mean of two biological replicates, with s.e.m. shown. For *in vitro* staining, a representative image from two biological replicates is shown.

Supplementary Figure 3: Differentiation of hPSCs into border or surface ectoderm within 48 hours, and comparison of posterior neural differentiation methods

A) qPCR of H1 hPSCs differentiated for 48 hours, using two separate strategies. First, hPSCs were differentiated into definitive ectoderm for 24 hours, followed by either anterior or posterior neural ectoderm induction for 24 hours (as described in this study). Alternatively, hPSCs were differentiated with BMP and TGFβ inhibitors (LDN-193189 and A-83-01, respectively; "2i") together with WNT agonist (CHIR99021, 2 μM) for 48 hours, similarly to a previous report [43]. Gene expression is shown relative to reference gene *YWHAZ*; 100% indicates that a gene is expressed at the same level as *YWHAZ*.

B) qPCR of H1 hPSCs differentiated into definitive ectoderm for 24 hours, followed by treatment with non-neural signals (A-83-01 and PD0325901), in combination with different levels of BMP signaling (BMP agonist BMP4 [0.05-30 ng/mL] or alternatively, BMP inhibitor LDN-193189), in the presence or absence of either WNT agonist (CHIR99021, 3 μM) or WNT inhibitor (C59, 1 μM), for 24 hours. White circles indicate optimal BMP doses for border ectoderm induction (0.5 ng/mL BMP4) or surface ectoderm induction (30 ng/mL BMP4). Gene expression is shown relative to reference gene *YWHAZ*; 100% indicates that a gene is expressed at the same level as *YWHAZ*.

C) qPCR of H1 hPSCs differentiated into definitive ectoderm for 24 hours, followed by border ectoderm-inducing signals (A-83-01, BMP4 [0.5 ng/mL], and PD0325901), in combination with different levels of WNT signaling (WNT agonist CHIR99021 [3-8 μM] or WNT inhibitor C59), for 24 hours.

D) qPCR of H1 hPSCs differentiated into definitive ectoderm for 24 hours, followed by border ectoderm-inducing signals (A-83-01, CHIR99021 [3 μM], and PD0325901), in combination with different levels of BMP signaling (BMP agonist BMP4 [0.05-30 ng/mL] or alternatively, BMP inhibitor LDN-193189), for 24 hours. Gene expression is shown relative to reference gene *YWHAZ*; 100% indicates that a gene is expressed at the same level as *YWHAZ*.

E) qPCR of H1 hPSCs differentiated into definitive ectoderm for 24 hours, followed by border ectoderm-inducing signals (A-83-01, BMP4, CHIR99021, and PD0325901) for 24 hours to generate border ectoderm. To assess the importance of each border ectoderm-inducing signal, each factor was individually withheld. Gene expression is shown relative to reference gene *YWHAZ*; 100% indicates that a gene is expressed at the same level as *YWHAZ*. D2 BE: day-2 border ectoderm.

F) qPCR of H1 hPSCs differentiated into definitive ectoderm for 24 hours, followed by surface ectoderm-inducing signals (A-83-01, C59, and PD0325901), in the presence or absence of BMP4 (0-30 ng/mL), for 24 hours. Gene expression is shown relative to reference gene *YWHAZ*; 100% indicates that a gene is expressed at the same level as *YWHAZ*.

G) qPCR of H1 hPSCs differentiated into definitive ectoderm for 24 hours, followed by surface ectoderm-inducing signals (A-83-01, BMP4, C59, and PD0325901) for 24 hours.

To assess the importance of each border ectoderm-inducing signal, each factor was individually withheld. Gene expression is shown relative to reference gene *YWHAZ*; 100% indicates that a gene is expressed at the same level as *YWHAZ*. D2 SE: day-2 surface ectoderm.

qPCR data depicts the mean of two biological replicates, with s.e.m. shown.

Supplementary Figure 4: Differentiation of hPSCs into anterior neural, posterior neural, border or surface ectoderm within 48 hours, and lineage tracing of neural ectoderm cells within mouse embryos

A) Summary of the hPSC differentiation strategy described in this study.

B) qPCR of H7 human embryonic stem cells (hESCs), SUN004.1 human induced pluripotent stem cells (hiPSCs), and SUN004.2 hiPSCs differentiated into definitive ectoderm for 24 hours, followed by either anterior neural ectoderm, posterior neural ectoderm, border ectoderm or surface ectoderm, for 24 hours. The heatmap depicts the Z-score, indicating the variation of gene expression across cell-types for a given gene; two biological replicates were used per cell-type.

C) qPCR of H1 hPSCs differentiated into definitive ectoderm for 24 hours, followed by differentiation into either aNE, pNE, border ectoderm or surface ectoderm for 24 hours. Gene expression is shown relative to reference gene *YWHAZ*.

D) Top: *In situ* hybridization of H1 hPSCs differentiated into definitive ectoderm for 24 hours, followed by differentiation into aNE or pNE for 24 hours. Scale: 100 μm. Bottom: Immunostaining of H1 hPSCs differentiated into definitive ectoderm for 24 hours, followed by differentiation into border or surface ectoderm for 24 hours. Scale: 100 μm.

E) *In situ* staining of E9.5 *Gbx2-CreER*; *ROSA26-tdTomato* mouse embryo that was not exposed to 4OHT. This negative control revealed minimal *Gbx2-CreER*-driven recombination in the absence of 4OHT. Scale bar = 500 μm.

F) E7.0 *Gbx2-CreER*; *ROSA26-LoxP-STOP-LoxP-tdTomato* mouse embryos were exposed to 1 mg 4OHT (intraperitoneally delivered into pregnant females) to label *Gbx2*$^+$ pNE with tdTomato. Subsequently, *in situ* staining was performed on E8.25 embryos to visualize pNE-derived progeny in relation to the expression of *Wnt1* (which marks the midbrain/hindbrain boundary) and *Gbx2* (which marks the hindbrain). Scale bar = 100 μm.

G) E7.5 *Sox2-CreER*; *Confetti* mouse embryos were exposed to 0.5 mg 4OHT (intraperitoneally delivered into pregnant females) to label single E7.5 *Sox2*$^+$ neural ectoderm progenitors with one of three detectable fluorescent colors (red, cyan, or yellow). Subsequently, E9.5 embryos were immunostained to visualize neural ectoderm-derived cell clusters and where they resided in relation to the forebrain, midbrain, and hindbrain; photos of three independent embryos are shown (*left*). The forebrain/midbrain domain was molecularly determined by Otx2 immunostaining (*left*). Quantification of the locations of all neural ectoderm-derived cell clusters analyzed in this study, totaling 494 cell clusters from 16 embryos, obtained from 7 litters (*right*). Scale bar = 100 μm.

H) Detailed analysis of the spatial position of a E7.5 *Sox2*$^+$ neural ectoderm-derived cell cluster relative to the midbrain/hindbrain boundary. E7.5 *Sox2-CreER*; *Confetti* mouse embryos were exposed to 0.5 mg 4OHT (intraperitoneally delivered into pregnant females) to label single E7.5 *Sox2*$^+$ neural ectoderm progenitors with one of three detectable fluorescent colors (red, cyan, or yellow). Subsequently, E9.5 embryos were

immunostained to visualize neural ectoderm-derived cell clusters and where they resided in relation to the forebrain, midbrain, and hindbrain. The forebrain/midbrain domain was molecularly determined by Otx2 immunostaining. This is a higher magnification image of an embryo shown in **Fig. S4g**. Asterisks indicate autofluorescence from red blood cells (RBCs). Scale bar = 100 μm.

Supplementary Figure 5: Differentiation of hPSCs into forebrain or midbrain progenitors within 4 days

A) qPCR of H1 hPSCs differentiated into definitive ectoderm for 24 hours, followed by anterior neural ectoderm for 24 hours, and then subsequently treated with either basal medium (CDM2) for 48 hours, or supplemented with WNT inhibitor DKK1 (LRP5/6 inhibitor, 300 ng/mL), LGK974 (Porcupine inhibitor, 100 nM), C59 (Porcupine inhibitor, 1 μM), or XAV939 (Tankyrase inhibitor, 1 μM). This revealed that XAV939 was most effective at upregulating forebrain marker *FOXG1*. Gene expression is shown relative to reference gene *YWHAZ*; 100% indicates that a gene is expressed at the same level as *YWHAZ*.

B) qPCR of H1 hPSCs differentiated into definitive ectoderm for 24 hours, followed by anterior neural ectoderm for 24 hours, and then subsequently treated with XAV939 for 48 hours to generate forebrain progenitors. During the 48 hour period of forebrain induction, FGF2 (20 ng/mL) was added for the first 24 hour interval ("day 3"), the second 24 hour interval ("day 4"), or the entirety of the 48 hour interval ("days 3-4"), in the presence or absence of BMP receptor inhibitor LDN193189 (100 nM). This revealed that day 3 FGF2 treatment, together with BMP inhibition on days 3-4, was most effective at upregulating forebrain markers *FOXG1* and *SIX3*. Gene expression is shown relative to reference gene *YWHAZ*; 100% indicates that a gene is expressed at the same level as *YWHAZ*.

C) qPCR of H7 hPSCs differentiated into definitive ectoderm for 24 hours, followed by anterior neural ectoderm for 24 hours, and then subsequently treated with XAV939 + LDN193189 + FGF2 (2-100 ng/mL) for 24 hours, and finally XAV939 + LDN193189 for 24 hours to generate forebrain progenitors. On the third day of differentiation ("D3"), different doses of FGF2 (2-100 ng/mL) were tested, or alternatively, exogenous FGF2 was withheld (no notation), in the presence or absence of FGFR inhibitor PD173074 (100 ng/mL). This revealed that 20 ng/mL of FGF2 on day 3 of differentiation was most effective at upregulating forebrain markers *SIX3* and *FOXG1*, consistent with how FGF induces *Foxg1* in the mouse embryonic forebrain[203]. Gene expression is shown relative to reference gene *YWHAZ*; 100% indicates that a gene is expressed at the same level as *YWHAZ*.

D) qPCR of H1 hPSCs differentiated into definitive ectoderm for 24 hours, followed by anterior neural ectoderm for 24 hours, and then subsequently treated with XAV939 + LDN193189 + FGF2 for 24 hours, and finally XAV939 + LDN193189 for 24 hours to generate forebrain progenitors. To assess the importance of each forebrain-inducing signal (XAV939, LDN193189 or FGF2), each individual signal was respectively withheld during the 48 hour interval of forebrain induction. Gene expression is shown relative to reference gene *YWHAZ*; 100% indicates that a gene is expressed at the same level as *YWHAZ*.

E) qPCR of H1 hPSCs differentiated into definitive ectoderm for 24 hours, followed by anterior neural ectoderm for 24 hours, and then subsequently treated with the following WNT pathway modulators for 48 hours: WNT inhibitor (XAV939, 1 μM), RSPO2 (25 nM), RSPO2 + WNT3A (increasing concentrations of 10, 100 or 1000 ng/mL), RSPO2 + NGS WNT-Fc[201] (increasing concentrations of 0.03, 0.3 or 3 nM), or CHIR99021 (increasing concentrations of 0.75, 1.5 or 3 μM). For WNT3A or NGS WNT-Fc treatment conditions, RSPO2 was added to increase cell-surface levels of FRIZZLED receptors, thereby enhancing cellular responsiveness to these WNT pathway agonists[201]. NGS-WNT-Fc refers to an Fc-tagged synthetic WNT pathway agonist that acts by heterodimerizing FZD and LRP6 receptors[201].

F) qPCR of H1 hPSCs differentiated into definitive ectoderm for 24 hours, followed by anterior neural ectoderm for an additional 24 hours, and then subsequently treated with either WNT inhibitor (XAV939, 1 μM) or WNT agonist (CHIR99021, 1.5-4 μM) for an additional 48 hours. This showed that WNT induced midbrain progenitors, whereas WNT inhibitor specified forebrain progenitors. Gene expression is shown relative to the sample with the highest expression in this experiment.

G) qPCR of H1 hPSCs differentiated into definitive ectoderm for 24 hours, followed by anterior neural ectoderm for 24 hours, and then subsequently differentiated towards midbrain with WNT agonist (CHIR99021, 3 μM) for 48 hours, in the presence or absence of FGF2 (20 ng/mL) on the first 24 hour interval, the second 24 hour interval, or the entire 48 interval of midbrain induction. Gene expression is shown relative to reference gene *YWHAZ*; 100% indicates that a gene is expressed at the same level as *YWHAZ*.

H) qPCR of H1 hPSCs differentiated into definitive ectoderm for 24 hours, followed by anterior neural ectoderm for 24 hours, and then subsequently differentiated towards midbrain with WNT agonist (CHIR99021, 3 μM) for 48 hours, in the presence or absence of FGF2 (2-200 ng/mL) on last 24 hours of midbrain induction. Gene expression is shown relative to reference gene *YWHAZ*; 100% indicates that a gene is expressed at the same level as *YWHAZ*.

I) qPCR of H1 hPSCs differentiated into definitive ectoderm for 24 hours, followed by anterior neural ectoderm for 24 hours, and then subsequently differentiated towards midbrain for 48 hours (CHIR99021 on days 3-4, and FGF2 on day 4). To assess the importance of each midbrain-inducing signal (CHIR99021 or FGF2), each individual signal was respectively withheld during the 48 hour interval of midbrain induction.

J) *In situ* staining of an E8.5 *Tcf/Lef:H2B:Venus* reporter mouse embryo[173] for *Foxg1* (forebrain marker) and *Venus* (indicating WNT transcriptional response). This disclosed a posteriorizing gradient of WNT signaling, with minimal WNT signaling in *Foxg1*$^+$ forebrain, but high WNT signaling in the midbrain.

qPCR data depicts the mean of two biological replicates, with s.e.m. shown.

Supplementary Figure 6: Differentiation of hPSCs into hindbrain progenitors within 4 days

A) qPCR of H1 hPSCs differentiated into definitive ectoderm for 24 hours, followed by posterior neural ectoderm for 24 hours, and then further differentiated for 48 hours in the presence or absence of FGF2 (10-200 ng/mL) or FGFR inhibitor (PD173074, 100 nM). qPCR data normalized to undifferentiated hPSCs. Gene expression is shown

relative to reference gene *YWHAZ*; 100% indicates that a gene is expressed at the same level as *YWHAZ*. FGFR: FGF receptor.

B) qPCR of H1 hPSCs differentiated into definitive ectoderm for 24 hours, followed by posterior neural ectoderm for 24 hours, and then further differentiated for 48 hours in FGF2 (20 ng/mL), in the presence or absence of WNT agonist (CHIR99021, 0.5-1.5 μM) or WNT inhibitor (XAV939, 1 μM). Gene expression is shown relative to reference gene *YWHAZ*; 100% indicates that a gene is expressed at the same level as *YWHAZ*.

C) qPCR of H1 hPSCs differentiated into definitive ectoderm for 24 hours, followed by posterior neural ectoderm for 24 hours, and then further differentiated into hindbrain for 48 hours by FGF2 + CHIR99021. Alternatively, during the 48 hour interval of hindbrain induction, either FGF2 or CHIR99021 were individually withheld. This revealed that both FGF and WNT signaling are required for maximal *MAFB* expression. qPCR data normalized to levels found in hindbrain progenitors (e.g., cells treated with FGF2 + CHIR99021).

D) qPCR of H1 hPSCs differentiated into definitive ectoderm for 24 hours, followed by posterior neural ectoderm for 24 hours, and then further differentiated into hindbrain for 48 hours by FGF2 + CHIR99021. Alternatively, during the 48 hour interval of hindbrain induction, either FGF2 or CHIR99021 were individually withheld for 24 intervals ("day 3" = the first 24 hour interval, whereas "day 4" = the second 24 hour interval). This revealed that both FGF and WNT signaling are continuously required for 48 hours to achieve maximal *MAFB* expression. Gene expression is shown relative to reference gene *YWHAZ*; 100% indicates that a gene is expressed at the same level as *YWHAZ*.

E) qPCR of H1 hPSCs differentiated into definitive ectoderm for 24 hours, followed by posterior neural ectoderm for 24 hours, and then further differentiated into hindbrain by FGF2 + CHIR99021 for 24, 48 or 72 hours (*left*). This revealed that FGF and WNT treatment for 48 hours was optimal for hindbrain specification (as denoted by a white circle), which was used as a baseline for the remainder of this study. qPCR data normalized to undifferentiated hPSCs. Cartoon of hPSC differentiation into hindbrain progenitors, with various signaling pathways activated or inhibited in a temporally dynamic way at each stage (*right*).

F) scRNAseq of H7 hPSC-derived day-4 hindbrain progenitors, and day-7 dorsal hindbrain or ventral hindbrain progenitors. Colors denote differentiation conditions. An asterisk (*) indicates that the legend is identical to that shown in **Fig. 4c**, and is reproduced here to aid the interpretation of other images shown in this subpanel.

G) H1 hPSCs were differentiated into definitive ectoderm for 24 hours, and then subsequently differentiated into either anterior neural ectoderm (aNE) or posterior neural ectoderm (pNE) within an additional 24 hours. hPSC-derived aNE and pNE cells were then dissociated and then replated in either forebrain-, midbrain-, or hindbrain-inducing signals for 48 hours, and then immunostaining was performed. Scale bar = 50 μm

qPCR data depicts the mean of two biological replicates, with s.e.m. shown. For *in vitro* staining, a representative image from two biological replicates is shown.

Supplementary Figure 7: Accessible chromatin landscapes and lineage commitment of hPSC-derived anterior vs. posterior neural ectoderm

A) H1 hPSCs were differentiated into either anterior or posterior neural ectoderm for 2 days. They were then respectively challenged with either forebrain-, midbrain-, or hindbrain-

inducing signals for 2, 3 or 4 days. i) qPCR was performed on days 4, 5 and 6 of hPSC differentiation. Gene expression is shown relative to reference gene *YWHAZ*; 100% indicates that a gene is expressed at the same level as *YWHAZ*. ii) Immunostaining was performed on day 6 of differentiation. Scale = 100 μm.

B) H1 hPSCs were differentiated into either aNE or pNE within 2 days; pNE was fluorescently labeled, and then aNE (uncolored) and pNE (dye-labeled) were mixed. Cocultures were treated with either forebrain-, midbrain-, or hindbrain-inducing signals for 4 days, prior to immunostaining (i.e., immunostaining was performed on day 6 of hPSC differentiation). aNE: day-2 anterior neural ectoderm. pNE: day-2 posterior neural ectoderm.

C) OmniATACseq was performed on day 2 H7 hPSC-derived aNE vs. pNE. A Z-score heatmap is shown, depicting all differentially accessible regions between these two cell-types (false discovery rate [FDR]<0.05). Each row indicates an individual genomic element.

D) OmniATACseq was performed on day 2 H7 hPSC-derived aNE vs. pNE that were challenged with forebrain-inducing signals for 2 additional days (1 day of XAV939 [1 μM] + LDN193189 [100 nM] + FGF2 [20 ng/mL], followed by 1 day of XAV939 (1 μM) and LDN193189 [100 nM]). A Z-score heatmap is shown, depicting all differentially accessible regions between these two cell-types (FDR<0.05). Each row indicates an individual genomic element.

E) OmniATACseq and RNAseq of hPSC-derived aNE and pNE that were treated with either forebrain- or hindbrain-inducing signals for 48 hours.

F) OmniATACseq was performed on day 2 H7 hPSC-derived aNE vs. pNE that were challenged with hindbrain-inducing signals (CHIR99021 [750 nM] + FGF2 [20 ng/mL]) for 2 additional days. A Z-score heatmap is shown, depicting all differentially accessible regions between these two cell-types (FDR<0.05). Each row indicates an individual genomic element.

G) Top 25 differentially expressed genes between H7 hPSC-derived day-2 anterior neural ectoderm vs. posterior neural ectoderm, as determined by scRNAseq. The top 25 differentially expressed genes were determined by P value, and all these genes showed statistically significant expression differences (P<0.05).

H) Single-cell RNA-sequencing of H7 hPSCs differentiated into day-1 definitive ectoderm, day-2 anterior neural ectoderm, day-2 posterior neural ectoderm, day-2 border ectoderm, or day-2 surface ectoderm. Colors denote differentiation conditions. *Fzd5*, *Lhx5*, and *Fezf1* have previously been associated with anterior neural identity *in vivo* at later developmental stages[204-206], whereas *Meis2* has been previously associated with posterior neural identity *in vivo*[207].

qPCR data depicts the mean of two biological replicates, with s.e.m. shown. For *in vitro* staining, a representative image from two biological replicates is shown. OmniATACseq and bulk-population RNAseq were each performed on two biological replicates. *In vitro* scRNAseq data is from a single experiment.

Supplementary Figure 8: Differentiation of hPSCs into dorsal forebrain and ventral forebrain progenitors within 7 days

A) H1 hPSCs were differentiated into definitive ectoderm within 24 hours, and then anterior neural ectoderm within 24 hours, followed by forebrain progenitors within 48 hours.

Forebrain progenitors were then treated with Vismodegib + PD173074 for 72 hours, in the presence or absence of WNT agonist (CHIR99021, 1-6 μM) or WNT inhibitor (XAV939, 1 μM). This revealed that 2 μM of CHIR99021 (indicated by white circle) was optimal for inducing dorsal forebrain marker *EMX2*. Gene expression is shown relative to reference gene *YWHAZ*; 100% indicates that a gene is expressed at the same level as *YWHAZ*. HH inh.: HEDGEHOG inhibitor. FGFR inh.: FGF receptor inhibitor.

B) H1 hPSCs were differentiated into definitive ectoderm within 24 hours, and then anterior neural ectoderm within 24 hours, followed by forebrain progenitors within 48 hours. Forebrain progenitors were then treated with CHIR99021 (2 μM) + PD173074 (100 nM) for 72 hours, in the presence or absence of FGF2 (20 ng/mL) or FGFR inhibitor (PD173074, 100 nM). This revealed that FGF inhibition enhanced dorsal forebrain markers *EMX2* and *PAX6*, while preventing erroneous WNT-induced expression of midbrain marker *EN1*. Gene expression is shown relative to reference gene *YWHAZ*; 100% indicates that a gene is expressed at the same level as *YWHAZ*. FGFRi: FGF receptor inhibitor.

C) H1 hPSCs were differentiated into definitive ectoderm within 24 hours, and then anterior neural ectoderm within 24 hours, followed by forebrain progenitors within 48 hours. Forebrain progenitors were then treated with CHIR99021 (2 μM) + PD173074 (100 nM) for 72 hours, in the presence or absence of BMP4 (10 ng/mL) or BMPR inhibitors (DMH1 [250 nM] or LDN193189 [100 nM]). Gene expression is shown relative to reference gene *YWHAZ*; 100% indicates that a gene is expressed at the same level as *YWHAZ*. BMPRi: BMP receptor inhibitor.

D) H1 hPSCs were differentiated into definitive ectoderm within 24 hours, and then anterior neural ectoderm within 24 hours, followed by forebrain progenitors within 48 hours. Forebrain progenitors were then treated with XAV939 (1 μM) in the presence or absence of increasing doses of HEDGEHOG pathway agonist 21K (0.1-5 nM) for 72 hours to generate ventral forebrain. This showed that high HEDGEHOG pathway activation, together with WNT inhibition, led to maximum expression of ventral forebrain markers. Gene expression is shown relative to the sample with the highest expression in this experiment. HH: Hedgehog pathway agonist. WNTi: WNT inhibitor.

E) H1 hPSCs were differentiated into definitive ectoderm within 24 hours, and then posterior neural ectoderm within 24 hours, followed by hindbrain progenitors within 48 hours. Hindbrain progenitors were then treated with HEDGEHOG inhibitor (Vismodegib, 150 nM) and WNT agonist (CHIR99021, 2 μM) for 72 hours to generate dorsal forebrain, in the presence or absence of BMP agonist (BMP4, 10 ng/mL) or BMPR inhibitor (LDN193189, 100 nM). This showed that BMP inhibition promoted expression of dorsal forebrain markers *GSX2* and *PAX3*. Gene expression is shown relative to reference gene *YWHAZ*; 100% indicates that a gene is expressed at the same level as *YWHAZ*. BMPRi: BMP receptor inhibitor.

F) H1 hPSCs were differentiated into definitive ectoderm within 24 hours, and then posterior neural ectoderm within 24 hours, followed by hindbrain progenitors within 48 hours. Hindbrain progenitors were then treated with HEDGEHOG pathway inhibitor Vismodegib (150 nM) in the presence or absence of increasing doses of WNT pathway agonist CHIR99021 (1-4 μM) for 72 hours to generate dorsal forebrain. This showed that moderate WNT pathway activation, together with HH inhibition, led to maximum expression of dorsal hindbrain markers *GSX2* and *OLIG3*. Gene expression is shown

relative to reference gene *YWHAZ*; 100% indicates that a gene is expressed at the same level as *YWHAZ*. BMPRi: BMP receptor inhibitor.

G) scRNAseq of H7 hPSC-derived day-4 hindbrain progenitors, and day-7 dorsal hindbrain or ventral hindbrain progenitors. Colors denote differentiation conditions. An asterisk (*) indicates that the legend is identical to that shown in **Fig. 4c**, and is reproduced here to aid the interpretation of other images shown in this subpanel.

H) H1 hPSCs were differentiated into definitive ectoderm within 24 hours, and then posterior neural ectoderm within 24 hours, followed by hindbrain progenitors within 48 hours. Hindbrain progenitors were then treated with either Vismodegib (150 nM) + CHIR99021 (2 μM) + DMH1 (250 nM) for 72 hours to generate dorsal forebrain, or alternatively, 21K (5 nM) + XAV939 (1 μM) for 72 hours to generate ventral forebrain. Gene expression is shown relative to the sample with the highest expression in this experiment.

I) H1 hPSCs were differentiated into definitive ectoderm within 24 hours, and then posterior neural ectoderm within 24 hours, followed by hindbrain progenitors within 48 hours. Hindbrain progenitors were then treated with WNT pathway inhibitor XAV939 (1 μM) in the presence or absence of increasing doses of HEDGEHOG pathway agonist 21K (50 pM-5 nM) for 72 hours to generate ventral hindbrain. This showed that high doses of HEDGEHOG agonist, together with WNT inhibitor, were required to maximally induce ventral hindbrain markers. Gene expression is shown relative to the sample with the highest expression in this experiment. WNT inh.: WNT inhibitor.

qPCR data depicts the mean of two biological replicates, with s.e.m. shown. *In vitro* scRNAseq data is from a single experiment.

Supplementary Figure 9: Differentiation of hPSCs into dorsal and ventral forebrain and dorsal and ventral hindbrain neurons within 14 days

A) scRNAseq of H7 hPSC-derived dorsal forebrain, ventral forebrain, dorsal hindbrain and ventral hindbrain populations on day 14 of differentiation. An asterisk (*) indicates images identical to those shown in **Fig. 5d**, which were reproduced here to aid the interpretation of other images shown in this subpanel.

B) scRNAseq of H7 hPSC-derived dorsal forebrain populations on day 14 of differentiation reveals expression of Cajal-Retzius neuron markers [208].

C) scRNAseq of H7 hPSC-derived ventral forebrain populations on day 14 of differentiation reveals expression of cortical interneuron markers.

D) scRNAseq of H7 hPSC-derived ventral forebrain populations on day 14 of differentiation reveals expression of hypothalamus-associated markers. hPSC-derived groups I and II express *PROX1* [209], *LHX5* [210], and *ADCYAP1* [140,141], which are expressed *in vivo* by various regions of the hypothalamus, including the preoptic area, paraventricular nucleus, and supraoptic nucleus. hPSC-derived group I is additionally defined by *OTP*, the archetypic transcription factor required for the development of paraventricular and supraoptic nuclei [211]. Instead, hPSC-derived group II is additionally defined by *POU4F1* (*BRN3A*) and *EBF1*, and may correspond to an early *Pou4f1*+ *Ebf1*+ *Pomc*- hypothalamic lineage *in vivo* [212]. hPSC-derived group II also expresses *ADCYAP1R1* (the ADYCAP1 receptor), which is expressed by multiple brain regions, including the preoptic area, paraventricular nucleus, and supraoptic nucleus [213]. Finally, hPSC-

derived group III is demarcated by *ISL1* and *POMC*, which are expressed within the arcuate nucleus of the hypothalamus [142].

E) qPCR of H1 hPSC-derived day-7 ventral hindbrain progenitors differentiated towards neurons for 7 additional days. Gene expression is shown relative to the sample with the highest expression in this experiment.
F) scRNAseq of H7 hPSC-derived ventral hindbrain populations on day 14 of differentiation reveals expression of multiple ventral hindbrain neuron subtype markers (*left*), whose identities were extrapolated from ventral spinal cord neuron subtype markers [214]. Cartoon of progenitor domains within the developing ventral hindbrain, based on embryological studies (*right*) [214]. The box reflects the neuron subtypes putatively produced in our *in vitro* differentiation system.
G) scRNAseq of H7 hPSC-derived dorsal hindbrain populations on day 14 of differentiation reveals expression of multiple dorsal hindbrain neuron subtype markers (*left*) [132,145]. Cartoon of progenitor domains within the developing ventral hindbrain, based on embryological studies (*right*) [132,145]. The box reflects the neuron subtypes putatively produced in our *in vitro* differentiation system.

In vitro scRNAseq data is from a single experiment. qPCR data depicts the mean of two biological replicates, with s.e.m. shown.

Supplementary Figure 10: **Functional assessment of hPSC-derived dorsal and ventral hindbrain neurons, and evolutionary conservation of anterior and posterior ectoderm**

A) Summary of the current-voltage relationship of voltage-dependent K^+ currents from dorsal and ventral hindbrain neurons. Currents were measured from -90 mV to +50 mV holding potentials. n (number of cells) = 4. Data are mean ± S.E.M.
B) Summary of the current-voltage relationship of voltage-dependent Na^+ currents from dorsal and ventral hindbrain neurons. Currents were measured from -90 mV to +50 mV holding potentials. n (number of cells) = 4. Data are mean ± S.E.M.
C) Summary of the number of spikes elicited by prolonged current injection.
D) Day 28 H1 hPSC-derived ventral hindbrain neurons transduced with AAVdj-*CAG-bReaChES-eYFP* were stimulated with either prolonged (1 second), or pulses of (5 ms, 5 Hz), 560nm light, and electrophysiological recording was performed to detect action potentials.
E) Electrophysiological activity of H1 hPSC-derived dorsal hindbrain neurons on day 30 of differentiation, showing voltage-dependent K^+ (left) or Na^+ (right) channel currents elicited by depolarization of the holding potential from -90 mV to 50 mV in voltage-clamp mode.
F) Current-clamp characterization of H1 hPSC-derived dorsal hindbrain neurons on day 30 of differentiation, showing action potentials elicited by injection of pulsed (500 pA, 5 ms, 10 Hz, left) or prolonged (50 pA, 1.5 seconds, right) currents in current-clamp mode.
G) Live Ca^{2+} imaging of WTC11 *AAVS1-CAG-GCaMP6f* hPSCs differentiated into dorsal hindbrain neurons for 30 days, which exhibited spontaneous Ca^{2+} transients as assessed within individual cells (left) and across the culture (right). Scale bar = 50 μm.
H) *In situ* staining of acorn worm embryo at the mid blastula stage. A maximum intensity projection of optical sections focusing on the ectoderm is shown. Scale bar = 100 μm.

Ca^{2+} imaging and electrophysiology were each performed on a single experiment. *In vivo* staining was performed on a single embryo.

Materials and Methods

Mouse models

Gbx2$^{CreER/+}$ mice[174] were originally developed by, and generously provided by, James Li's laboratory (JAX #022135) and maintained on a CD1 background, heterozygous for the targeted allele. Mice carrying the *Gbx2^{CreER}* allele were identified by PCR as described by Jackson Laboratory.

Sox2$^{CreER/+}$ mice[116] were originally developed by Konrad Hochedlinger's laboratory, were obtained from Jackson Laboratory (JAX #017593), and maintained on a C57BL/6 background, heterozygous for the targeted allele. Mice carrying the *Sox2^{CreER}* allele were identified by PCR as described by Jackson Laboratory.

ROSA26-CAG-LoxP-STOP-LoxP-tdTomato ("Ai14") mice[111] were originally developed by the Allen Brain Institute, were obtained from Jackson Laboratory (JAX #007914), and were maintained homozygous for the targeted allele.

ROSA26-Confetti mice[175] were originally developed by Hans Clevers's laboratory, were obtained from Jackson Laboratory (JAX #017492), and were respectively maintained as homozygous for the targeted allele.

Tcf/Lef:H2B-GFP mice[173] were originally developed by Anna-Katerina Hadjantonakis's laboratory, were obtained from the Jackson Laboratory (#013752), and maintained on a CD1 background, heterozygous for the targeted allele. These mice were shared by Teni Anbarchian and Roel Nusse.

Mouse husbandry and lineage tracing

Mice of the desired genetic backgrounds were mated, leading to timed pregnancies. Noon on the day a vaginal plug was detected was designated as E0.5. For Gbx2^{CreER}-mediated lineage tracing, 1 mg of (*Z*)-4-Hydroxytamoxifen (henceforth referred to as 4OHT; Sigma, H7904-25MG) was administered. For Sox2^{CreER}-mediated lineage tracing, 0.5 mg of 4OHT was administered.

4OHT was resuspended and administered as described previously [115]. In brief, a 20 mg/mL stock of 4OHT was made by dissolving 25 mg of 4OHT powder in 1250 μL of ethanol. This solution was heated for 10 minutes at 60°C and sonicated briefly for 10-20 seconds. This 20 mg/mL stock of 4OHT was aliquoted to generate 50 μL aliquots (i.e., 1 mg per aliquot), which were stored at -20°C.

When ready to inject, a 50 μL aliquot of 4OHT was thawed at 65°C for 10 minutes. During the thawing process, the solution was vortexed and quickly centrifuged in a microcentrifuge every 2-3 minutes. Simultaneously, 250 μL of corn oil (Sigma, C8267-500mL) was warmed to 65°C. After the 4OHT solution was thawed for 10 minutes, 250 μL of warm corn oil was then added to 4OHT solution, thereby generating a final concentration of 3.33 mg/mL of 4OHT. This mixture

was heated at 65°C for 45 minutes, with vortexing every 15 minutes. This 3.33 mg/mL of 4OHT solution was delivered via intraperitoneal injection, using a U-100 Insulin Syringe (Comfort Point, 26028), to pregnant female mice at the relevant labeling timepoint.

Mouse embryos of specific ages were obtained from pregnant mice. The biological sex of mouse embryos was not determined.

In situ hybridization of whole mount mouse embryos

Hybridization chain reaction v3.0 (HCR3) [176] was used to perform whole-mount fluorescent *in situ* hybridization (FISH) of mouse embryos. Embryos were dissected in ice-cold 4% paraformaldehyde and subsequently fixed either for 1 hour (for E8.0 and earlier embryonic stages), or alternatively overnight (for E8.25 and later embryonic stages), prior to methanol dehydration. Hybridization mRNA probes, amplifiers, and buffers were obtained from Molecular Instruments, and the protocol for whole-mount staining of mouse embryos were followed as per the manufacturer's instructions with the following modifications: Proteinase K digestions varied with embryonic stage; E6.5: 5 min; E7.5: 7.5 min; E8.5: 10 min; E9.5: 15 min. Embryos were incubated in SSCT + DAPI prior to mounting.

Immunostaining of whole mount mouse embryos

Embryos were dissected in ice-cold PBS and fixed overnight on a rocker at 4C in 1% paraformaldehyde. They were washed 3 x 10 min in PBS at room temperature before being blocked overnight in embryo blocking buffer (3% BSA, 0.2% Tween-20 in PBS). Embryos were then incubated with primary antibodies (diluted in embryo blocking buffer) for 48 hours at 4C with gentle rocking. Subsequently, the embryos were washed for 4 hours in PBST (0.2% Tween-20 in PBS) before being transferred to incubation with secondary antibodies for 48 hours at 4C with gentle rocking. Embryos were cleared overnight in Rapiclear 1.49 (Sunjin labs) before being mounted, also in Rapiclear 1.49.

Imaging of mouse embryos

Images were collected at room temperature on Olympus FV3000, Leica SP8, or Zeiss LSM980 confocal microscopes using 405, 488, 514, 561, and 640 nm wavelengths and 4X/0.16, 10X/0.4, and 20X/0.75 dry objectives, and 40X/1.3 oil objective. Data was displayed with FIJI ImageJ software[177].

Computational analysis of mouse embryo images in which neural ectoderm cells were sparsely labeled

Sox2-CreER;Confetti mouse embryo images were imported into Imaris 10 for quantification. Sox2+ neural ectoderm-derived cell clusters were identified in CFP, YFP, and RFP channels separately using the Spots function. Cell clusters were identified as spots within 50 µm of each other in the same channel using the Imaris SplitSpots plugin function. A surface feature of the forebrain and midbrain were generated from the Otx2 immunofluorescence signal in the far-red channel. Cell clusters were defined relative to the forebrain/midbrain surface element. Sox2+ neural ectoderm-derived cell clusters with less than 2 cells across the midbrain-hindbrain boundary were assigned to the region in which the majority of cells resided.

Computational analysis of differentiated hPSC immunofluorescence images

Immunofluorescence images of differentiated hPSCs were quantified using either Fiji [177] or Imaris 9 to determine the percentage of marker-positive cells. The DAPI channel was used to define the nucleus of each individual cell, which was then defined as marker-positive or marker-negative using a threshold.

Maximum intensity projections of immunostaining images were imported into Fiji[177] for cell quantification. A threshold of marker intensity was manually set to define marker-positive and marker-negative cells based on negative controls (i.e., cells that did not express the protein of interest). Subsequently, to define the border of each individual nucleus, we used the DAPI channel and employed the Analyze Particles function in Fiji, using specific size and circularity values that we kept consistent within each image. After the border of each individual nucleus was defined, the Analyze Particles function was used to determine whether the nucleus was marker-positive or marker-negative, in relation to the aforementioned marker intensity threshold.

Separately, Z-stacks of immunostaining images were imported into Imaris 9 to generate 3-dimensional models. For all fluorescence channels, we performed background subtraction via auto-thresholding to reduce noise. We then analyzed the DAPI channel, filtered the DAPI channel via the Intensity Mean function, and the Spots function was used to define the border of each individual nucleus, with a 7 μm radius for each nucleus in 3-dimensional space. To define whether each nucleus was marker-positive or marker-negative, we applied the Intensity Mean function on the fluorescence of each given marker. A minimum value for the Intensity Mean function was manually determined. The same threshold was used for a given marker across all cell-types analyzed.

Cell culture

All cells in this study were cultured in standard incubator conditions (20% O_2, 5% CO_2 and 37 °C).

Basement membrane matrix for cell culture experiments

hPSCs were maintained and differentiated on cell culture plates that been pre-coated with Geltrex basement membrane matrix, as described previously [178]. Geltrex (Thermo Fisher) was diluted 1:100 in DMEM/F12 (Thermo Fisher) and was kept at 4 °C. To coat cell culture plates, Geltrex solution was added to cell culture plastics for at least 1 hour at 37 °C. The volume of Geltrex solution added was roughly equivalent to half the working volume of the well or dish (e.g., 1 mL or 0.5 mL of Geltrex solution was added per well of a 6-well or 12-well plate, respectively). After coating with Geltrex, the basement membrane solution was aspirated, leaving behind a thin film; subsequently, cells were plated on the Geltrex-coated cell culture plastics.

A detailed protocol to coat cell culture plastics with Geltrex is available [179].

Human pluripotent stem cell culture

Multiple hPSC lines, including both human embryonic stem cells (hESCs) and human induced pluripotent stem cells (hiPSCs) were used in this study: wild-type H1, H7, and H9 hESCs [180] (WiCell), wild-type SUN004.1 and SUN004.2 hiPSCs [178] (provided by Hiromitsu Nakauchi's

laboratory), HES3 *MIXL1-GFP* hPSCs [181] (provided by Andrew Elefanty's, Edouard Stanley's, and Elizabeth Ng's laboratories), and WTC11 *AAVS1-CAG-GCaMP6f* hiPSCs [182] (provided by Bruce Conklin's laboratory). Of note, SUN004.1 and SUN004.2 represent two different, clonally-derived hiPSC lines reprogrammed from peripheral blood mononuclear cells obtained from the same healthy adult volunteer (SUN004)[178].

Undifferentiated hPSCs were maintained as described previously [178] in either mTeSR1 or mTeSR Plus media (StemCell Technologies) supplemented with 1% penicillin/streptomycin (Thermo Fisher), on Geltrex-coated cell culture plates. When the undifferentiated hPSC cultures became partially confluent, undifferentiated hPSCs were passaged by treating them with EDTA (Versene, Thermo Fisher) for 7 minutes at room temperature. After removal of EDTA, mTeSR was re-added and the plate was manually scraped to generate clumps, which were reseeded onto new Geltrex-coated plates.

A detailed protocol to culture and passage undifferentiated hPSCs is available [179].

hPSC differentiation into ectodermal derivatives

The day before beginning differentiation ("day -1" [D-1]), hPSCs were enzymatically dissociated using Accutase (Thermo Fisher) and plated as single cells from 30,000-100,000 cells/cm^2 on Geltrex-coated cell culture plates in mTeSR or mTeSR Plus + 1% penicillin/streptomycin + ROCK inhibitor thiazovivin (1 μM, Tocris). Inclusion of ROCK inhibitor was critical to ensure survival of single cells during seeding.

After 24 hours, cells were ready for subsequent differentiation. The first 7 days of hPSC differentiation were conducted in Chemically Defined Medium 2 (CDM2), whose composition has been described previously [73,74]. CDM2 comprises 50% IMDM + GlutaMAX (Thermo Fisher, 31980-097), 50% F12 + GlutaMAX (Thermo Fisher), polyvinyl alcohol (1 mg/mL, Sigma) + chemically defined lipid concentrate (1% v/v, Thermo Fisher), 1-thioglycerol (450 μM, Sigma), recombinant human insulin (0.7 μg/mL, Sigma), human transferrin (15 μg/mL, Sigma), penicillin/streptomycin (1% v/v, Thermo Fisher). To create CDM2, polyvinyl alcohol was brought into suspension by gentle warming and magnetic stirring, and the media was filtered through a 0.22 μm filter prior to use.

A detailed protocol to passage hPSCs for differentiation, and to prepare CDM2 basal medium, is available [179].

Definitive ectoderm (D0-1, 24 hours): D0 hPSCs were briefly washed before differentiation with DMEM/F12 to remove any traces of mTeSR Plus. They were then differentiated toward definitive ectoderm in CDM2 media supplemented with A8301 (1 μM, Tocris), Trametinib (250 nM, Cellagen Technology), C59 (1 μM, Tocris), and LDN193189 (100 nM, Stemgent) for 24 hours. Subsequently, D1 definitive ectoderm was used for either anterior neural ectoderm, posterior neural ectoderm, border ectoderm, or surface ectoderm induction.

Anterior neural ectoderm (D1-2, 24 hours): D1 definitive ectoderm cells were resplit using Accutase (Thermo Fisher) and plated as single cells on Geltrex-coated cell culture plates in CDM2 supplemented with A8301 (1 μM, Tocris), LDN193189 (100 nM, Stemgent), C59 (1 μM,

Tocris), and Thiazovivin (1 µM, Tocris) for 24 hours. Thiazovivin was added to promote cell survival after enzymatic passaging. For experiments ending after two days of differentiation, cells were plated at 37,500 cells/cm^2, and for experiments ending after more than 2 days of differentiation (i.e., towards a forebrain fate), 75,000 cells/cm^2 were plated. After 24 hours of anterior neural ectoderm media, cells were used for either forebrain or midbrain induction.

Posterior neural ectoderm (D1-2, 24 hours): D1 definitive ectoderm cells were resplit using Accutase (Thermo Fisher) and plated as single cells on Geltrex-coated cell culture plates in CDM2 supplemented with A8301 (1 µM, Tocris), LDN193189 (100 nM, Stemgent), TTNPB (50 nM, Tocris), FGF2 (20 ng/mL, R&D Systems), XAV939 (1 µM, Tocris), and Thiazovivin (1 µM, Tocris) for 24 hours. Thiazovivin was added to promote cell survival after enzymatic passaging. For experiments ending after two days of differentiation, cells were plated at 37,500 cells/cm^2, and for experiments ending after more than 2 days of differentiation (i.e., toward a hindbrain fate), 150,000 cells/cm^2 were plated. After 24 hours of posterior neural ectoderm media, cells were used for hindbrain induction.

Border ectoderm (D1-2, 24 hours): D1 definitive ectoderm cells were resplit using Accutase (Thermo Fisher) and plated as single cells at 37,500 cells/cm^2 on Geltrex-coated cell culture plates in CDM2 supplemented with A8301 (1 µM, Tocris), PD0325901 (100 nM, Tocris), CHIR99012 (3 µM, Tocris), BMP4 (0.5 ng/mL, R&D Systems), and Thiazovivin (1 µM, Tocris) for 24 hours. Thiazovivin was added to promote cell survival after enzymatic passaging.

Surface ectoderm (D1-2, 24 hours): D1 definitive ectoderm cells were resplit using Accutase (Thermo Fisher) and plated as single cells at 37,500 cells/cm^2 on Geltrex-coated cell culture plates in CDM2 supplemented with A8301 (1 µM, Tocris), PD0325901 (100 nM, Tocris), BMP4 (30 ng/mL, R&D Systems), C59 (1 µM, Tocris), and Thiazovivin (1 µM, Tocris) for 24 hours. Thiazovivin was added to promote cell survival after enzymatic passaging.

Forebrain (D2-4, 48 hours): D2 anterior neural ectoderm cells were briefly washed with DMEM/F12 prior to addition of forebrain media. D3 forebrain media consisted of CDM2 supplemented with XAV939 (1 µM, Tocris), LDN193189 (100 nM, Stemgent), and FGF2 (20 ng/mL, R&D Systems), and was changed after 24 hours into D4 forebrain media, which consisted of CDM2 supplemented with XAV939 (1 µM) and LDN193189 (100 nM). Subsequently, forebrain cells could be further differentiated toward either dorsal forebrain or ventral forebrain fates.

Midbrain (D2-4, 48 hours): D2 anterior neural ectoderm cells were briefly washed with DMEM/F12 prior to addition of midbrain media. D3 midbrain media consisted of CDM2 supplemented with CHIR99021 (3 µM, Tocris) and was changed after 24 hours into D4 midbrain media, which consisted of CDM2 supplemented with CHIR99021 (3 µM) and FGF2 (20 ng/mL, R&D Systems).

Hindbrain (D2-4, 48 hours): D2 posterior neural ectoderm cells were briefly washed with DMEM/F12 prior to addition of hindbrain media. D3 and D4 hindbrain media consisted of CDM2 supplemented with CHIR99021 (0.75 µM, Tocris) and FGF2 (20 ng/mL, R&D Systems).

Hindbrain media was replaced after 24 hours. Subsequently, hindbrain cells could be further differentiated toward either dorsal hindbrain or ventral hindbrain fates.

Dorsal Forebrain (D4-7, 72 hours): D4 forebrain progenitors were briefly washed with DMEM/F12 prior to addition of dorsal forebrain media. D5, D6, and D7 dorsal forebrain media consisted of CDM2 supplemented with CHIR99021 (2 μM, Tocris), Vismodegib (150 nM, Selleck Chemicals), and PD173074 (100 nM, Tocris). Dorsal forebrain differentiation was conducted for 72 hours, and dorsal forebrain media was replaced every 24 hours.

Ventral Forebrain (D4-7, 72 hours): D4 forebrain progenitors were briefly washed with DMEM/F12 prior to addition of ventral forebrain media. D5, D6, and D7 dorsal forebrain media consisted of CDM2 supplemented with XAV939 (1 μM, Tocris) and SAG 21K (5 nM, Tocris). Ventral forebrain differentiation was conducted for 72 hours, and ventral forebrain media was replaced every 24 hours.

Dorsal Hindbrain (D4-7, 72 hours): D4 hindbrain progenitors were briefly washed with DMEM/F12 prior to addition of dorsal hindbrain media. D5, D6, and D7 dorsal hindbrain media consisted of CDM2 supplemented with CHIR99021 (2 μM, Tocris), Vismodegib (150 nM, Selleck Chemicals), and LDN193189 (100 nM, Tocris). Dorsal hindbrain differentiation was conducted for 72 hours, and dorsal hindbrain media was replaced every 24 hours.

Ventral Hindbrain (D4-7, 72 hours): D4 hindbrain progenitors were briefly washed with DMEM/F12 prior to addition of ventral hindbrain media. D5, D6, and D7 ventral hindbrain media consisted of CDM2 supplemented with XAV939 (1 μM, Tocris) and SAG 21K (5 nM, Tocris). Ventral hindbrain differentiation was conducted for 72 hours, and ventral hindbrain media was replaced every 24 hours.

Neuronal differentiation (D7-14, 7 days): hPSC-derived day 7 dorsal forebrain, day 7 ventral forebrain, day 7 dorsal hindbrain, and day 7 ventral forebrain progenitors were subsequently differentiated into neurons for 7 days. Neuronal differentiation media was modified from a previous report [139]. From day 8-14, cells were differentiated in BDNF (20 ng/ml, PeproTech), GDNF (20 ng/ml, PeproTech), Forskolin (10 μM, Tocris), 2-Phospho-L-ascorbic acid (200 μg/ml, Sigma-Aldrich), and DAPT (10 μM, R&D Systems), with different basal media used on each day of differentiation. The general approach was to gradually transition differentiating cells from CDM2 to Neurobasal differentiation media. Media composition was as follows: 1) D7-8 media (i.e., first 24 hours of neuronal differentiation) contained: 25% Neurobasal media (see below) + 75% CDM2 media was added; 2) D8-9 media (i.e., second 24 hours of neuronal differentiation): 50% Neurobasal media + 50% CDM2 media was added; 3) D9-10 (i.e., third 24 hours of neuronal differentiation), 75% Neurobasal media + 25% CDM2 media was added; 4) D10-14 (i.e., remaining 96 hours of neuronal differentiation), 100% Neurobasal media. In other words, throughout the entire neuronal differentiation phase, cells were exposed to the same concentration of neuronal differentiation signals (BDNF, GDNF, Forskolin, 2-Phospho-L-ascorbic acid, and DAPT) while a gradual change in basal media occurred. "Neurobasal media" is comprised of Neurobasal media (Thermo Fisher) supplemented with 1% v/v N2 (Thermo Fisher), 2% v/v B27 w/o Vitamin A (Thermo Fisher), and 1% v/v penicillin/streptomycin (Thermo Fisher). The media was passed through a sterilizing 0.22 μM filter before use.

hPSC differentiation into non-ectodermal cell-types
As a positive control to achieve MIXL1-GFP expression, hPSCs were differentiated into mid primitive streak as described previously [78], by culturing them in Activin A (30 ng/mL) + BMP4 (40 ng/mL) + CHIR99021 (6 μM) + FGF2 (20 ng/mL) in CDM2 basal media for 24 hours.

To generate definitive endoderm and paraxial mesoderm, hPSCs were first differentiated into anterior primitive streak as described previously [78], by culturing them in Activin A (30 ng/mL) + CHIR99021 (4 μM) + FGF2 (20 ng/mL) in CDM2 basal media for 24 hours. hPSC-derived anterior primitive streak was then briefly washed with DMEM/F12 and then treated with either definitive endoderm-inducing media [77] (Activin A [100 ng/mL] + LDN193189 [250 nM] in CDM2 basal media) for 24 hours, or paraxial mesoderm-inducing media [78] (A-83-01 [1 μM] + LDN193189 [250 nM] + CHIR99021 [3 μM] + FGF2 [20 ng/mL]) for 24 hours.

Fluorescent labeling of hPSC-derived cells for coculture experiments
Day 2 anterior and posterior neural ectoderm (aNE and pNE, respectively) were generated as described above, and then associated using Accutase (Thermo Fisher). aNE cells were kept on ice, while pNE cells were resuspended at 10^6 cells/mL in prewarmed PBS containing 1-5 μM ViaFluor 488 Cell Proliferation dye (Biotium) for 10 minutes at 37 °C. Dye was prepared on the day of the labeling. Cells were washed with DMEM/F12 (Thermo Fisher) prior to pelleting. For mixed cultures, aNE and dye-labeled pNE were mixed at a 1:1 ratio prior to plating and subsequent culture in either forebrain, midbrain, or hindbrain media, as described. After 2-4 days of culture in either forebrain, midbrain, or hindbrain media, cells were fixed and stained as described above, prior to imaging.

Quantitative PCR
Cells were lysed with RNeasy Lysis Buffer (Qiagen) and RNA was subsequently extracted using the RNeasy Plus Kit (Qiagen) as per the manufacturer's instructions. RNA was reverse transcribed into cDNA using the High-Capacity cDNA Reverse Transcription Kit (Applied Biosystems). qPCR was subsequently performed on the cDNA with the SensiFAST SYBR Lo-ROX Kit (Bioline) on a QuantStudio 5 qPCR machine (Thermo Fisher). qPCR primer sequences are provided in **Table S1**. The cycling parameters were as follows: initial dissociation (95 °C, 2 mins) followed by 40 cycles of amplification and SYBR signal detection (95 °C dissociation, 5 seconds; 60 °C annealing, 10 seconds; followed by 72 °C extension, 30 seconds), with a final series of steps to generate a dissociation curve at the end of each run. During qPCR data analysis, the fluorescence threshold to determine Ct values was set at the linear phase of amplification. Expression of all genes was normalized to the levels of the reference gene *YWHAZ*.

In histograms that depict qPCR data, the y-axis label of "% *YWHAZ*" indicates that the expression level of each gene is depicted relative to the expression of reference gene *YWHAZ*, such that "100%" indicates that the gene is expressed at the same level of *YWHAZ*. Alternatively, other histograms depicting qPCR data carry a y-axis label of "Expression relative to highest value", which indicates that the expression of each gene is depicted relative to the biological sample with the highest value of that gene in a given experiment.

qPCR histograms depict the mean of two biological replicates, defined as two independent wells treated with the same differentiation media in a single experiment. Two technical qPCR replicate measurements were performed on each biological replicate. Error bars represent the standard error of the mean (s.e.m.).

qPCR primer sequences used to target human genes are as follows:

Primers	Forward	Reverse
ACHE	GGGTGGTAGACGCTACAACC	GTGCCCTCAAAACCTGGGTAT
AXIN2	CAACACCAGGCGGAACGAA	GCCCAATAAGGAGTGTAAGGACT
BRACHYURY/T	TGCTTCCCTGAGACCCAGTT	GATCACTTCTTTCCTTTGCATCAAG
CDX2	GGGCTCTCTGAGAGGCAGGT	CCTTTGCTCTGCGGTTCTG
CHT/SLC5A7	CACTGCTGTGCATGCCAAAT	TCCACCCAGCATCAACAACA
CK8/KRT8	CAGAAGTCCTACAAGGTGTCCA	CTCTGGTTGACCGTAACTGCG
EMX2	CGGCACTCAGCTACGCTAAC	CAAGTCCGGGTTGGAGTAGAC
EN1	GGACAATGACGTTGAAACGCAGCA	AAGGTCGTAAGCGGTTTGGCTAGA
EN2	CCGGCGTGGGTCTACTGTA	CCTCTTTGTTCGGGTTCTTCTT
EOMES	CAACATAAACGGACTCAATCCCA	ACCACCTCTACGAACACATTGT
FOXG1	CCGCACCCGTCAATGACTT	CCGTCGTAAAACTTGGCAAAG
GATA3	TCCTGTGCGAACTGTCAGAC	TCGGTTTCTGGTCTGGATGC
GBX2	TCCCGGAACCCTAAGATCGTCG	ACAGCTGGGCTGTGACTTTGTTGC
GLI1	AACGCTATACAGATCCTAGCTCG	GTGCCGTTTGGTCACATGG
GSX2	ATGTCGCGCTCCTTCTATGTC	CAAGCGGGATGAAGAAATCCG
HNF1B	ACCAAGCCGGTCTTCCATACT	GGTGTGTCATAGTCGTCGCC
HOXA1	CGTGAGAAGGAGGGTCTCTTG	GTGGGAGGTAGTCAGAGTGTC
HOXA2	CAGAACCGGAGGATGAAGCA	ACGCTAAGGGCTTGCTCAAA
HOXA3	AGCAGCTCCAGCTCAGGCGAAA	TGGCGCTCAGTGAGGTTCAG
HOXB1	CTGGGTCAATCAGAAGGAGACG	GTCTGCTCGTTCCCATAAGGG
HOXB3	CAACAGCAAATCTCCTTGGACCG	ACAAATCTCCCCTCCTTTGCG
ISL1	AGATTATATCAGGTTGTACGGGATCA	ACACAGCGGAAACACTCGAT
KROX20/EGR2	TCAACATTGACATGACTGGAGAG	AGTGAAGGTCTGGTTTCTAGGT
LEF1	AGAACACCCCGATGACGGA	GCATCATTATGTACCCGGAAT
LHX2	TCGGGACTTGGTTTATCACCT	GCAAGCGGCAGTAGACCAG
MAFB	TCAAGTTCGACGTGAAGAAGG	GTTCATCTGCTGGTAGTTGCT
MAPT/TAU	CCAAGTGTGGCTCATTAGGCA	CCAATCTTCGACTGGACTCTGT
MEIS2	CTCGTCATTGATGAAAGAGACGG	TCAGCGAGATTTGTGGAGGAG
MIXL1	GGTACCCCGACATCCACTTG	TAATCTCCGGCCTAGCCAAA
NANOG	CATGAGTGTGGATCCAGCTTG	CCTGAATAAGCAGATCCATGG
NGN2	TGGGTCTGGTACACGATTGC	GTCTTCTTGATGCGCTG
NKX2.1	CTCATGTTCATGCCGCTC	GACACCATGAGGAACAGCG

NKX2.2	ATGTCGCTGACCAACACAAAG	GATGTCCTTGACCGAAAACCC
NKX6.1	ACACGAGACCCACTTTTTCCG	GCCCCGCCAAGTATTTTGTT
NKX6.2	GAGGACGACGACGAATACAAC	GTTCGAGGGTTTGTGCTTCTT
OCT4/POU5F1	AGTGAGAGGCAACCTGGAGA	ACACTCGGACCACATCCTTC
OLIG2	CCAGAGCCCGATGACCTTTTT	CACTGCCTCCTAGCTTGTCC
OLIG3	CCTGCTCGCCAGAAACT	CCCCATAGATCTCGCCAACC
OTX2	GGAAGCACTGTTTGCCAAGACC	CTGTTGTTGGCGGCACTTAGC T
PAX2	GAGCGAGTTCTCCGGCAAC	GTCAGACGGGGACGATGTG
PAX3	AGACTGGCTCCATACGTCCT	CATGCCCGGGTTCTCTCTTT
PAX5	AAACCAAAGGTCGCCACAC	GTTGATGGAACTGACGCTAGG
PAX6	AGTGCCCGTCCATCTTTGC	CGCTTGGTATGTTATCGTTGGT
PAX7	TCCAAGATTCTTTGCCGCTAC	GGTCACAGTGCCCATCCTTC
PHOX2A	CACTTGGCGCCCTACGC	GCTCCTCACGCGTGTAAATG
PHOX2B	AACCCGATAAGGACCACTTTTG	AGAGTTTGTAAGGAACTGCGG
SIX3	CTGCCCACCCTCAACTTCTC	GCAGGATCGACTCGTGTTTGT
SNAP25	ACCAGTTGGCTGATGAGTCG	CAAAGTCCTGATACCAGCATCT T
SOX2	TGGACAGTTACGCGCACAT	CGAGTAGGACATGCTGTAGGT
SP5	GGGCGGTTCCAAACTCTAA	CCCAAACCGGTCCTAGC
TFAP2A	TGCCAACGTTACCCTGCTCACAT C	CCAGCAGGTCGGTGAACTCTT TGC
VACHT/SLC18 A3	GGCATAGCCCTAGTCGACAC	CGTAGGCCACCGAATAGGAG

Immunocytochemistry of hPSC-derived cell-types

Adherent cells were washed once with PBS, fixed for 10 minutes in 4% paraformaldehyde (Fisher Scientific) at room temperature, and then washed three more times with PBS. They were subsequently permeabilized with permeabilization/blocking solution (0.1% Triton-X100 + 5% donkey serum + PBS) for 1 hour at 4 ºC. Subsequently, primary antibodies were added in permeabilization/blocking solution overnight at 4 ºC. Cells were then washed three times with PBS, and secondary antibodies were added in permeabilization/blocking solution for 1 hour at 4 ºC. Cells were then washed twice with PBS, and then nuclear counterstaining was performed with 0.2 µg/mL DAPI + PBS for 10 minutes at room temperature. Imaging was conducted on an Olympus FV3000 confocal microscope.

***In situ* hybridization of hPSC-derived cell-types**

In situ hybridization of hPSC-derived anterior and posterior neural ectoderm was performed using hybridization chain reaction v3.0 (HCR3)[176], in accordance with the Molecular Instruments protocol for cultured mammalian cells. In brief, DNA probe sets were generated by Molecular Instruments, Inc., targeting the RNA sequences of the *Mus musculus* genes *Otx2, Gbx2*, and *Sox2*. In brief after generating hPSC-derived cell types, cells were fixed with 4% formaldehyde, watched twice with PBS, and permeabilized overnight at -20 degrees Celsius in 70% ethanol. Cells were then washed twice with 5x sodium chloride sodium citrate plus 0.1% Tween20 (SSCT), and prehybridized in probe hybridization buffer (from Molecular Instruments). The probe solution was prepared by adding 1.2 pmol of each probe set (*Otx2,*

Gbx2, and *Sox2*) to 300µL of probe hybridization buffer at 30 degrees Celsius, and added to cells after removing the pre-hybridization buffer. Samples were then incubated overnight at 37 degrees Celsius. Cells were then washed four times with probe wash buffer (from Molecular Instruments) and twice with SSCT. Cells were pre-amplified with 300µL of amplification buffer (from Molecular Instruments). Separately, 18 pmol of hairpin h1 and 18 pmol of hairpin h2 (B1-647, B2-546, and B3-488) were prepared by snap cooling 6µL of 3µM stock. Pre-amplification buffer was then removed from samples and replaced with the hairpin solution. Samples were incubated overnight in the dark at room temperature. Samples were washed 5 times with SSCT and prepared for confocal imaging.

Western Blot

hPSC-derived anterior and posterior neural ectoderm cells were dissociated with TrypLE Express, centrifuged at 1400 rpm for 5 minutes, washed in PBS, and then centrifuged again. Cells were then incubated on ice in RIPA buffer for 30-60 minutes to lyse them. RIPA buffer is composed of: 0.05 M Tris-HCL pH 7.5 (Thermo Fisher Scientific, 15567027), 0.15M NaCl (Thermo Fisher Scientific, J60434AE), 0.1% SDS (Thermo Fisher Scientific, 28364), 0.5% sodium deoxycholate (Millipore Sigma, D6750), 0.1% Triton X-100 (Millipore Sigma, T8787), 1:50 protease inhibitor (Millipore Sigma, 11697498001), and 1:10,000 benzonase nuclease (Millipore Sigma, E1014-25KU).

This whole cell lysate was then centrifuged at 12,000g for 10 minutes at 4 °C to remove debris from protein samples. Protein concentration was then quantified using the Pierce bicinchoninic acid (BCA) method (Pierce, 23227).

For each sample, 10 µg of total protein was combined with gel-loading dye (Boster Biological Technology, AR1112), boiled at 99 °C for 5 minutes, briefly centrifuged, and then loaded on a 4-12% Bis-Tris gel (Thermo Fisher Scientific, NW04125BOX). In another lane of the same gel, a protein ladder (Thermo Fisher Scientific, 26620) was added. Electrophoresis was conducted for 30 minutes at 200 volts in MOPS buffer (Thermo Fisher Scientific, B0001).

After electrophoresis, proteins were transferred to a nitrocellulose membrane using the Mini Blot Module (Thermo Fisher Scientific, B1000) for 1 hour at 15 volts. The nitrocellulose membrane was then blocked in blocking solution (PBS [Thermo Fisher, 10010023] + 5% milk [Bio-Rad, 1706404] + 0.1% Tween-20 [Sigma, 11332465001]). The blot was probed with primary antibodies for GBX2 (Thermo Fisher, PA5-66953, diluted 1:1000) and VINCULIN (Cell Signaling Technology, E1E9V, diluted 1:1000), diluted in blocking solution. Primary antibodies were incubated overnight at 4 °C on a shaker. Blots were then washed three times for 5 minutes each with PBS + 0.1% Tween-20 on a shaker. Subsequently, the blot was incubated with horseradish peroxidase (HRP)-conjugated secondary antibodies (Cell Signaling Technology, 7074, diluted 1:500), diluted in blocking solution. Secondary antibodies were incubated for 1 hour at room temperature on a shaker. Finally, blots were then washed three times for 5 minutes each with PBS + 0.1% Tween-20 on a shaker. Proteins were visualized using chemiluminescence substrates (Millipore Sigma, WBLUF0500) and imaged on a ChemiDoc imager (Bioradiations, 170-8280).

Flow cytometry

hPSCs and hPSC-derived ectoderm cells were dissociated by incubating them in TrypLE Express (Thermo Fisher) for 5 minutes at 37 °C. The resulting cell suspension was diluted 1:10 in FACS Buffer, which comprised 0.5% BSA (Thermo Fisher) and 2 mM EDTA (Thermo Fisher) in PBS, and subsequently pelleted at 300 x *g.*

Intracellular protein staining for flow cytometry was conducted using the Inside Stain Kit (Miltenyi Biotec) as per manufacturer's instructions.

Alternatively, live cells were analyzed, in which case no fixation or permeabilization was used.

Flow cytometry was performed on a Beckman Coulter CytoFlex analyzer (Stanford Stem Cell Institute FACS Core). For data analysis, cells were gated in FlowJo software based on forward and side scatter area parameters, followed by height and width parameters for doublet discrimination. An example of the flow cytometry gating strategy used for live cells is shown in **Supplementary Fig. 1**.

Bulk RNA-sequencing of hPSC-derived cell-types

Cells were lysed with RNeasy lysis buffer (Qiagen) and RNA was subsequently extracted using the RNeasy Plus Kit (Qiagen) as per the manufacturer's instructions. RNA-seq libraries were generated by Novogene and sequenced on an Illumina NovaSeq 6000.

Read quality control was performed using FastQC[183]. Subsequently, index adaptors and low-quality base-calls were removed using TrimGalore. RNA-seq reads were aligned to human reference genome hg38 using Kallisto[184]. Gene-level counts were derived from transcript-level counts using tximport[185].

BAM files were converted to bigwig files, which were then averaged across samples for visualization, using deepTools[186]. RNA-seq tracks were visualized in IGV[187].

Single-cell RNA-sequencing of hPSC-derived cell-types

hPSCs and differentiated ectoderm cells were enzymatically dissociated in Accutase (Thermo Fisher) at 37 °C. Day 14 neuron-containing populations were dissociated in papain (Worthington). All samples were run through a 40 µM filter prior to fixation for 24 hours at 4C and storage at -80C. Single cell gene expression was profiled using the Chromium Single Cell Gene Expression Flex kit (10x Genomics) according to the manufacturer's recommendations. A multiplexed experimental design was used in which 10,000 cells were targeted to be recovered per probe barcode, and 5000 cells per sample were targeted to be captured on Chip Q. Library preparation was confirmed using Agilent Bioanalyzer (High Sensitivity DNA kit), and libraries were sequenced on a NovaSeq 6000.

Computational analysis of single-cell RNA-sequencing data from hPSC-derived cell-types

Cell Ranger software[188] (10X Genomics) was used to perform read alignment to the hg38 human reference genome, barcode counting, and unique molecular identifier (UMI) counting. Downstream analysis was conducted using scanpy[189]. Two similar, but distinct, types of single-cell transcriptome quality control criteria were then applied.

First, for days 0-2 of differentiation: we filtered out (1) genes that were expressed in fewer than 3 cells and (2) cells with fewer than 3,000 UMIs, more than 5% mitochondrial reads, more than 100,000 UMIs, and those in which the reference gene *YWHAZ* was not detected.

Second, for all later days of differentiation: we filtered out (1) genes that were expressed in fewer than 3 cells and (2) cells with fewer than 2,000 UMIs, more than 15% mitochondrial reads, and more than 100,000 UMIs.

Subsequently, data were normalized to 10,000 reads per cell and $\log_e$ transformed. Expression of marker genes was denoted by different color shades and superimposed over the entire cell population as visualized on a Uniform Manifold Approximation and Projection (UMAP) projection[190]. All cells within the entire population that passed the aforementioned quality control criteria were shown on the UMAP projection, without pre-selecting any subset of cells.

The top 25 differentially expressed genes between day-2 anterior neural ectoderm vs. posterior neural ectoderm were determined, based on the fold change in expression between these two cell-types. A threshold of P value < 0.05 was applied to exclusively examine statistically significant differences in expression.

An interactive web browser to explore scRNAseq data of the hPSC-derived cell-types generated in this study is available: https://anglohlabs.shinyapps.io/dundes_jokhai_lohlab/.

OmniATAC-seq of hPSC-derived cell-types

OmniATAC-seq was performed using a modified version of the original protocol[191], using modifications described by Klaus Kaestner's laboratory (University of Pennsylvania). In brief, 50,000 live, freshly isolated cells from each sample were washed with 50 µl cold 1X PBS and pelleted. 50 µl of cold lysis buffer (0.1% v/v NP-40, 0.1% v/v Tween-20, 0.01% v/v Digitonin in resuspension buffer, comprised of 10 mM Tris-HCL, 10 mM NaCl, and 3 mM $MgCl_2$) was added to each sample and incubated for 3 minutes on ice. 1 mL of wash buffer (resuspension buffer with 0.1% v/v Tween-20) was added to each sample before centrifugation at 500xg for 10 minutes at 4C. The supernatant was discarded, and each nuclei pellet was resuspended in 50 µl of transposition buffer (25 µl 2X TD Buffer, 16.5 µl 1X PBS, 0.5 µl 10% Tween-20, 0.5 µl 1% Digitonin, 2.5 µl Tn5 Transposase, 5 µl nuclease-free water). The mixture was incubated for 30 minutes at 37°C on a thermomixer set to 1000 rpm.

DNA was isolated using the Qiagen MinElute Reaction Cleanup Kit, as per manufacturer's instructions. Libraries were generated using Illumina Nextera DNA Unique Dual Indexes and NEBNext High-Fidelity 2X PCR Master Mix. Libraries were double-side purified using AMPure XP beads, and quality was assessed using Agilent Bioanalyzer (High Sensitivity DNA kit). Sequencing was conducted on a NovaSeq 6000.

Computational analysis of OmniATAC-seq data

Read quality for ATAC-seq data was checked using FastQC and size-distribution and transcriptional start site (TSS) enrichment was confirmed using ATAQV. Reads were trimmed using NGMerge and aligned to human genome hg38 using bowtie2[192] with the flags --very-

sensitive -I 10 -X 2000. Duplicate reads were removed using samblaster, and samtools was used to convert file formats. Peaks were called on individual replicates using MACS2[193]. All peaks across all samples were merged and blacklisted regions were excluded using bedtools to create a set of peaks that define all possible accessible regions across the assayed cell-types.

TMM (Trimmed Mean of M-Values) normalization was performed on each ATAC-seq replicate to accurately account for differences in library composition and sequencing depth. First, featureCounts was used to count reads in accessible regions from each library. EdgeR was then used to calculate size factors for each library, and the final normalization factor was calculated as 1,000,000/(size factor * number of reads in peaks). Normalized bigwig files were generated for individual replicates by using deeptools, with scale factor equal to the final normalization factor that was calculated, and fragments larger than 120 bases were excluded. Replicates were then merged using wiggletools. Tracks were visualized in IGV[187]. Evolutionary conservation of genome sequence across 46 vertebrate species, as determined using Phastcons[194], was also depicted as an additional track in IGV.

DESeq2[195] was used to quantify differences in accessibility between cell-types using the count matrix generated from featureCounts. The statistical significance of differential accessibility was determined using the DESeq2 pipeline[195]. In brief, a Wald test was first applied to determine the P value. These P values were then adjusted for multiple hypothesis testing using the Benjamini-Hochberg procedure to controls for the false discovery rate, resulting in Q values.

Replicate similarity was confirmed by visual inspection of TMM normalized bigwigs and by PCA. Peaks were annotated using ChIPseeker, and volcano plots of differential regions between cell types were visualized using EnhancedVolcano.

Enriched motifs were determined using HOMER[196]. The statistical significance of motif enrichment was determined using the Fisher exact test, which is based on the hypergeometric distribution[196].

Ca^{2+} live imaging

WTC11 *AAVS1-CAG-GCaMP6f* hiPSCs[182] (which express the fluorescent Ca^{2+} sensor GCaMP6f[146]) were differentiated into dorsal and ventral hindbrain neurons in 14 days as described above. From days 15-30, neurons were maintained in the same neuron differentiation media described above (Neurobasal media + N2 + B27 + BDNF + GDNF + Forskolin + 2-Phospho-L-ascorbic acid + DAPT + penicillin/streptomycin), and this media was changed every other day. On day 30, cells were imaged on an Olympus FV3000 confocal microscope at room temperature with an air 20X/0.75 objective using a 488 nm laser at 7.9 Hz. Time-lapse movies were imported into FIJI ImageJ, where cells were identified with user-defined ROIs on a maximum intensity projection. A background ROI was also user-defined. For each frame, the pixel value of the background ROI was subtracted from each cellular ROI prior to calculating $\Delta F/F_0$. A representative subset of ROI traces was selected for plotting.

Electrophysiology

hPSC-derived neurons were placed in an extracellular Tyrode's medium (150 mM NaCl, 4 mM KCl, 2 mM $CaCl_2$, 2 mM $MgCl_2$, 10 mM HEPES [pH 7.3 - 7.4], and 10 mM glucose [osmolarity 335-345]). A borosilicate patch pipette (Harvard Apparatus) with a resistance of 5-6 MΩ was filled with intracellular medium (140 mM potassium-gluconate, 10 mM EGTA, 2 mM $MgCl_2$, and 10 mM HEPES [pH 7.2]). Signals were amplified and digitized using the Multiclamp 700B and DigiData1400 (Molecular Devices), and a Leica DM LFSA microscope was used to visualize the cells. Voltage-clamp experiments were conducted at a membrane potential of –70 mV.

For Na^+ and K^+ current measurement, cells were held at potentials from -80 mV to +50 mV in steps of 10 mV. Liquid junction potentials (LJPs) were corrected using the Clampex build-in LJP calculator as previously described[197]. Current clamp measurements in neurons were conducted with the current injected to hold the potential at -65 mV to -70 mV, followed by stepwise tonic current injection (from – 100 pA to 350 pA, 1.5 second) to elicit action potentials. Phasic currents (200-500 pA, 5 ms, 10 Hz) were injected to generate action potentials. Analyses of electrophysiological results were performed using ClampFit software (Axon Instruments).

Optogenetics

At day 21 of hPSC differentiation, ventral hindbrain neurons were transduced with adeno-associated viruses (AAVs) encoding either AAVdj-CAG-bReaChES-TS-EYFP or AAV8-hSyn-bReaChES-mCherry for 7 days. bReaChES encodes a red-shifted excitatory opsin[147]. Both *CAG* and human *SYNAPSIN* (hSYN) promoters have been shown to drive expression in motor neurons[198]. We confirmed that viral transduction was successful, owing to expression of the virally-encoded fluorescent protein (either EYFP or mCherry), as visualized using fluorescent microscopy.

On day 28 of hPSC differentiation (i.e., after 7 days of AAV transduction), optogenetic manipulation was performed. The Spectra X Light engine (Lumencor) served as a light source, and a 560 nm filter was used for orange light stimulation, followed by coupling into a Leica DM LFSA microscope.

Patch pipettes (3–6 MΩ) were pulled using a P2000 micropipette puller (Sutter Instruments). For the voltage clamp characterization, cells were recorded at -70 mV holding potential, and 1.0 mW/mm^2 light was delivered for 1 second. For the current clamp characterization, neurons were stimulated with 5-ms light pulses of 560 nm light at 1.0 mW/mm^2 with 5 Hz frequency.

Analysis of embryonic scRNAseq datasets

Single-cell RNA-sequencing data of gastrulating mouse embryos was described in a previous study (Pijuan-Sala et al., 2019)[169], and was downloaded from ArrayExpress accession number E-MTAB-6967. E6.75 epiblast cells were identified by filtering for cells with *Elf5* and *Rhox5* $\log_2$ normalized expression = 0, thereby excluding extraembryonic cells. E7.5 neural ectoderm cells were identified with $\log_2$ normalized expression of *Sox2* > 0.5, and *Nanog, Tfap2a, Rhox5, Foxj1,* and *Elf5* = 0.

Single-cell RNA-sequencing data of gastrulating non-human primate embryos was described in a previous study (Zhai et al., 2022)[170], and was downloaded from NCBI GEO accession

number GSE193007. Neural ectoderm cells were identified with log_2 normalized expression of *SOX2* > 1 and, *TFAP2A*, *HAND1*, and *APLNR* = 0.

Single-cell RNA-sequencing data of gastrulating chicken embryos was described in a previous study (Williams et al., 2022)[171], and was downloaded from NCBI GEO accession number GSE181577. Neural ectoderm cells were identified from the annotated "ectoderm" cluster with log_2 normalized expression of *Sox2* > 0.

Single-cell RNA-sequencing data of gastrulating zebrafish embryos was described in a previous study (Wagner et al., 2018)[172], and was downloaded from NCBI GEO accession number GSE112294. Neural ectoderm cells were identified with log_2 normalized expression of *sox2* > 0.5 and *tfap2a* < 0.5.

***In situ* hybridization of whole mount hemichordate embryos**

DNA probe sets were generated by Molecular Instruments, Inc., targeting the RNA sequences of the *Saccoglossus kowalevskii* genes *otx* (NM_001164888.1) and *gbx* (NM_001164886.1). Twenty probe sets were generated for each gene, and DNA oligo pools were resuspended to a final probe concentration of 1µmol/µl in 50 mM Tris buffer, pH 7.5. HCR amplifiers, B1-Alexa Fluor-546 for *otx* and B2-Alexa Fluor-647 for *gbx* were ordered from Molecular Instruments, Inc.

Hybridization chain reaction v3.0 (HCR3)[176] was performed as described previously, in combination with the EZ Clear tissue clearing method[199]. Embryos were fixed for 30 minutes at room temperature in fixation buffer (3.7% formaldehyde, 0.1 M MOPS pH 7.5, 0.5 M NaCl, 2 mM EGTA pH 8.0, 1 mM MgCl2, 1X PBS), and subsequently stored in ethanol (EtOH) at -20°C until use. Embryos were rehydrated from EtOH into RNAse-free water using an EtOH/water series (75%/25%, 50%/50%, 25%/75%, 100% water, 5 minutes each step) and then rinsed twice in water for 10 minutes. The fixed embryos were cleared overnight (12-16 hours) in 50% v/v tetrahydrofuran in water at 4ºC, followed by washing three times for 30 minutes each in water, and then twice for 5 minutes each in PBST (1X PBS + 0.1% Triton X-100). Embryos were then incubated in 500 µl of Detergent Solution (1% SDS, 0.5% Tween, 50 mM Tris-HCl, pH 7.5, 1 mM EDTA, pH 8.0, 150 mM NaCl) for 30 minutes. Samples were rinsed twice with PBST and then washed four times for 5 minutes each with PBST, followed by 5 minutes in 5X SSCT (5X sodium chloride sodium citrate, 0.1% Tween 20). Embryos were pre-hybridized in 100 µl of probe hybridization buffer (30% formamide, 5X sodium chloride sodium citrate, 9 mM citric acid, pH 6.0, 0.1% Tween 20, 50 µg/mL heparin, 1X Denhardt's solution, 10% dextran sulfate) for 1 hour at 37°C before adding 50 µl of probe solution (6 µl of each probe mixture to 50 µl final volume of probe hybridization buffer) and incubated overnight (12-16 hours) at 37°C. Samples were then washed 4 × 30 min with 1 mL of probe wash buffer (30% formamide, 5X sodium chloride sodium citrate, 9 mM citric acid, pH 6.0, 0.1% Tween 20 , 50 µg/mL heparin) at 37°C. Samples were further washed twice for 5 minutes each with 1 mL of 5X SSCT at room temperature. Embryos were then pre-amplified with 100 mL of amplification buffer (5X sodium chloride sodium citrate, 0.1% Tween 20, 10% dextran sulfate) for 30 minutes at room temperature before adding the prepared hairpin solution. Hairpin solution was prepared by heating 4 µl of hairpin h1 and 4 µl of hairpin h2 in separate tubes at 95°C for 90 seconds, then cooled to room temperature in the dark for 30 minutes. The h1 and h2 hairpins were

combined with 50 µl final volume amplification buffer, and then added to the pre-incubating samples. Samples were incubated in the hairpin solution overnight (12-16 hours) at room temperature in the dark. Hairpin solution was removed by washing with 1 mL of 5X SSCT at room temperature following a series (2 x 5 min, 2 x 30 min, 1 x 5 min). Finally, samples were incubated in NaCl-PBS Hoechst solution (1X PBS, 500 mM NaCl, 1:1000 Hoechst) in the dark at 4ºC overnight (12-16 hours) before rinsing in 1X PBS. Embryos were then mounted on slides. The PBS was removed from the embryos while on the slide, and then 30 µl of EZ Clear mounting medium (80% Nycodenz, 7M urea, 0.05% sodium azide, 0.02 M sodium phosphate) was added to clear the embryos. Imaging was conducted on a Zeiss LSM 700 confocal microscope using the Zen software package (Carl Zeiss). For each sample, series of optical sections were acquired with a z-step interval of 6 µm. Optical sections of ectodermal tissues were compiled into a maximum intensity z-projection using FIJI v2.14.0.

In situ **hybridization of whole mount zebrafish embryos**

Procedures involving zebrafish were approved by the University of California San Francisco IACUC. Adult zebrafish were kept at 28.5 °C in a 14-hour light and 10-hour dark cycle. All zebrafish husbandry was performed in accordance with institutional and national ethical and animal welfare guidelines. Sex/gender was not accounted for as zebrafish sex determination takes place later, during juvenile stages of development.

Zebrafish embryos at 8 hours post-fertilization (hpf) were fixed overnight in 4% paraformaldehyde at 4°C and gradually dehydrated into methanol. Whole mount *in situ* hybridization was performed using hybridization chain reaction v3.0 (HCR3)[176]. Hybridization mRNA probes, amplifiers, and buffers were purchased from Molecular Instruments, and the protocol provided by the manufacturer for whole mount zebrafish embryos was followed with no modifications. Embryos were mounted in ProLong Gold Antifade Mountant (Thermo Fisher, Cat# P10144) and imaged on an upright Stellaris 5 scanning confocal microscope with a Fluotar 25X water immersion lens. Images were visualized using Imaris to generate 3D renderings (Imaris 9.3.1, Oxford Instruments).

In situ **hybridization of whole mount chicken embryos**

Hybridization chain reaction v3.0 (HCR3)[176] was performed using the protocol suggested by Molecular Technologies, with minor modifications. Briefly, embryos were fixed in 4% paraformaldehyde (PFA) overnight at 4°C, washed in 0.1% PBS-Tween (PBSTw), dehydrated in a series of 25%, 50%, 75%, and 100% methanol washes, and stored in 100% methanol overnight at -20°C. The following day, embryos were rehydrated and incubated with 10 pmol of *Otx2* (B10 initiator), *Gbx2* (B2 initiator), and *Sox2* (B1 initiator) probes dissolved in hybridization buffer overnight at 37°C. The subsequent day, following several washes in hybridization probe wash buffer and 0.1% 5x-SSC-Tween, embryos were incubated in 30 pmol of amplifier hairpins H1 and H2 labeled with Alexa 488, Alexa 546, and Alexa 647 diluted in amplification buffer at room temperature overnight. The next day, embryos were washed in 0.1% 5x-SSC-Tween and imaged. All probes were designed and produced by Molecular Technologies.

Statistical analysis

As aforementioned, qPCR histograms depict the mean of two biological replicates, and error bars represent the standard error of the mean (s.e.m.). Sample sizes were based on those typically used in past studies from our laboratory. No datapoints were excluded, apart from qPCR technical replicates that failed. Experiments were repeated adequately to ensure reproducibility, and the number of experiments is generally described either in the figure legends or the **Methods** section. No randomization was performed. Investigators were not blinded. A two-tailed, unpaired t-test was used to test for statistical significance for qPCR data shown in histograms. One asterisk (*) denotes P<0.05, and two asterisks (**) denotes P< .01. For each experiment, we compared all values relative to the following controls:

**Figure**	**Control**
1G	Baseline condition (A8301 + LDN193189 + XAV939)
1H	SOX2: anterior neural ectoderm and posterior neural ectoderm<br>TFAP2A: border ectoderm and surface ectoderm<br>PAX3: border ectoderm<br>GATA3: surface ectoderm
2C	aNE → D4 lineage
2D	aNE → D4 lineage
2F	pNE → D4 lineage
Ext. 1B	D0 (undifferentiated day-0 hPSCs)
Ext. 1D	A8301/LDN
Ext. 1F	hPSC (undifferentiated day-0 hPSCs)
Ext. 1G	520 cells/mm2
Ext. 1H	hPSC (undifferentiated day-0 hPSCs)
Ext. 1I	SOX17: Endoderm<br>TBX6: Paraxial Mesoderm
Ext. 2A	D2 aNE (day-2 anterior neural ectoderm)
Ext. 2B	0 ng/mL FGF2 (for left panels) and 0 nM TTNPB (for right panels)
Ext. 2C	D2 pNE (day-2 posterior neural ectoderm)
Ext. 2E	A8301 (TGFβRi) + LDN193189 (BMPRi) + CHIR99021 (WNT)
Ext. 2F	Baseline condition: A8301 (TGFβRi) + LDN193189 (BMPRi)
Ext. 2G	Baseline condition: A8301 (TGFβRi) + LDN193189 (BMPRi) + TTNPB (RA) + FGF2
Ext. 2H	Def. ectoderm (day 1) : SOX2<br>Post. Neural ecto (day 2) : GBX2
Ext. 3A	2i + CHIR
Ext. 3B	0.5 ng/mL CHIR against 30 ng/mL C59
Ext. 3C	Baseline condition: A8301 (TGFβRi) + BMP4 (0.5 ng/mL) + PD0325901 (ERKi))
Ext. 3D	0.05 ng/mL BMP4
Ext. 3E	D2 BE (day-2 border ectoderm)
Ext. 3F	0 ng/mL BMP
Ext. 3G	D2 SE (day-2 surface ectoderm)
Ext. 5A	CDM2
Ext. 5B	D3-4 XAV939 (WNTi) + LDN

Ext. 5C	D3-4 XAV939 (WNTi) + LDN193189 (BMPRi)
Ext. 5D	Baseline condition: D3-4 XAV939 (WNTi) + D3-4 LDN193189 (BMPRi) + D3 FGF2
Ext. 5F	XAV
Ext. 5G	Baseline condition: D3-4 CHIR (3 µM)
Ext. 5H	Baseline condition: D3-4 CHIR (3 µM)
Ext. 5I	Baseline condition: Midbrain
Ext. 6A	Baseline condition: CDM2 basal medium alone
Ext. 6B	Baseline condition: D3-4 FGF2 (20 ng/mL)
Ext. 6C	Hindbrain
Ext. 6D	Hindbrain
Ext. 6E	D0 (undifferentiated day-0 hPSCs)
Ext. 7A	Day 4 pNE
Ext. 8A	Baseline condition: Vismodegib (HH inh.) + PD173074 (FGFR inh.)
Ext. 8B	Baseline condition: CHIR (2 µM) + Vismodegib (HH inh.)
Ext. 8C	Baseline condition: CHIR (2 µM) + PD173074 (FGFR inh.)
Ext. 8D	0 nM 21K was compared against 5 nM 21K
Ext. 8E	Baseline condition: CHIR (2 µM) + Vismodegib (HH inh.)
Ext. 8F	Baseline condition: Vismodegib (HH inh.)
Ext. 8H	NKX2.2, NKX6.1, NKX6.2, PHOX2B, and OLIG2: Ventral hindbrain<br>PAX3, PAX7, and GSX2: Dorsal hindbrain
Ext. 8I	5 nM 21K + XAV939
Ext. 9E	OLIG2, PHOX2A, PHOX2B: D7 Ventral Hindbrain Progenitor<br>ISL1, VACHT, ACHE, CHT, MAPT, SNAP25: D14 Ventral Hindbrain Neuron

Data collection
Confocal images were acquired on an Olympus FV3000 confocal microscope using Olympus Fluoview software. Flow cytometry data were acquired on a Beckman Coulter Cytoflex flow cytometer using BD Biosciences FACSDiva software. qPCR data were acquired on a Thermo Fisher QuantStudio 5 qPCR machine using QuantStudio Design and Analysis software. Deep sequencing was performed on a NovaSeq 6000 sequencer using NovaSeq Control Software. Electrophysiological signals were amplified and digitized using the Multiclamp 700B amplifier and DigiData 1440 digitizer (Molecular Devices) followed by data acquisition and recording with pCLAMP 10 software.

Data analysis
Confocal image analysis was performed using Imaris and ImageJ software. Flow cytometry data analysis was performed using FlowJo software. qPCR data analysis and statistical tests were performed on Microsoft Excel and GraphPad Prism software. Deep sequencing datasets were analyzed in Python, and further details regarding specific computational analyses of sequencing datasets are provided in the respective sections of the **Methods** statement. Electrophysiological data were analyzed using ClampFit software (Axon Instruments).

Data visualization
Confocal microscope images were plotted using Imaris and ImageJ software. Flow cytometry plots were plotted using FlowJo software. qPCR histograms were plotted using Microsoft Excel. Genomics plots were plotted using Python, with the exception of genomics tracks, which were

visualized using IGV[187]. qPCR heatmaps were generated using the online Heatmapper platform[200] (http://heatmapper.ca/). Animal silhouettes were modified from open-source images, including those from https://www.phylopic.org/. Figures were assembled with Microsoft PowerPoint.

Ethics and regulatory approval

All hPSC experiments were performed under the regulatory oversight of the Stem Cell Research Oversight (SCRO) committee. All experiments on living mice were performed under the regulatory oversight of the Administrative Panel on Laboratory Animal Care (APLAC) committee.

Data and materials availability

Bulk-population RNA-sequencing, single-cell RNA-sequencing, and OmniATAC-sequencing datasets generated as part of this study are available at the NCBI Gene Expression Omnibus (GEO): accession numbers GSE286146 (OmniATAC-sequencing), GSE286147 (bulk RNA-sequencing), and GSE286148 (single-cell RNA-sequencing). These datasets are grouped under SuperSet accession number GSE286214. An interactive web browser to explore single-cell RNA-sequencing data of the hPSC-derived cell-types generated in this study is available: https://anglohlabs.shinyapps.io/dundes_jokhai_lohlab/.

In this study, we also analyzed published single-cell RNA-sequencing datasets of gastrulating embryos; these datasets were generated from various species. Single-cell RNA-sequencing data of gastrulating mouse embryos were generated in a previous study (Pijuan-Sala et al., 2019) [169], and was downloaded from ArrayExpress accession number E-MTAB-6967. Single-cell RNA-sequencing data of gastrulating non-human primate embryos were generated in a previous study (Zhai et al., 2022) [170], and was downloaded from NCBI GEO accession number GSE193007. Single-cell RNA-sequencing data of gastrulating chicken embryos were generated in a previous study (Williams et al., 2022) [171], and was downloaded from NCBI GEO accession number GSE181577. Single-cell RNA-sequencing data of gastrulating zebrafish embryos were generated in a previous study (Wagner et al., 2018) [172], and was downloaded from NCBI GEO accession number GSE112294.

Computational scripts used in this study to analyze sequencing datasets are available on GitHub: https://github.com/lohlaboratory/ane-pne.

Experimental reagents

Reagent or Resource	Source	Identifier
**Antibodies**		
Rabbit anti-TFAP2A (for immunostaining)	Abcam	ab108311, concentration 1:250
Mouse anti-GATA3 (for immunostaining)	Thermo Fisher	MA1-028, concentration 1:1000
Mouse anti-PAX3 (for immunostaining)	R&D Systems	mAB2457, concentration 1:50
Goat anti-SOX2 (for immunostaining)	R&D Systems	AF2018, concentration 1:100

Rabbit anti-RFP (for immunostaining)	Rockland	600-401-379, concentration 1:500
Goat anti-5-HT (for immunostaining)	Immunostar	20079, concentration 1:500
Goat anti-OTX2 (for immunostaining)	R&D Systems	AF1979, concentration 1:200
Mouse anti-LHX2 (for immunostaining)	Invitrogen	MA5-15834, concentration 1:2000
Mouse anti-EN1 (for immunostaining)	DSHB	4611, concentration 1:35
Goat anti-HNF1B (for immunostaining)	R&D Systems	AF3330, concentration 1:250
Rabbit anti-MAFB (for immunostaining)	Sigma	HPA005653, concentration 1:800
Rabbit anti-HOXA3 (for immunostaining)	Novus	NBP1-83234, concentration 1:100
Rabbit anti-SIX3 (for immunostaining)	Rockland	600-401-A26S, concentration 1:1000
Mouse anti-PAX6 (for immunostaining)	Santa Cruz Biotechnology	sc81649, concentration 1:50
Rabbit anti-NKX2.1 (for immunostaining)	Abcam	ab76013, concentration 1:250
Rabbit anti-OLIG3 (for immunostaining)	Sigma	HA018303, concentration 1:1000
Rabbit anti-OLIG2 (for immunostaining)	Millipore	AB9610, concentration 1:500
Mouse anti-TAU/MAPT (for immunostaining)	Millipore	MAB3420, concentration 1:1000
Rabbit anti-TBR1 (for immunostaining)	Abcam	ab31940, concentration 1:2000
Rabbit anti-DLX1 (for immunostaining)	Thermo Fisher	PA5-28899, concentration 1:1000
Mouse anti-VGLUT2 (for immunostaining)	Thermo Fisher	MA5-31378, concentration 1:200
Rabbit anti-VGAT (for immunostaining)	Synaptic	131 003, concentration 1:1000
Rabbit anti-CHT/SLC5A7 (for immunostaining)	Abcam	ab56074, concentration 1:100
Mouse anti-ISL1/2 (for immunostaining)	DSHB	39.4D5, concentration 1:500
Rabbit anti-TLX3 (for immunostaining)	Thermo Fisher	PA5-83146, concentration 1:500
Rabbit anti-NANOG (for immunostaining)	Abcam	AB21624, concentration 1:200
Rabbit anti-GBX2 (for immunostaining)	Proteintech	21639-1-AP, concentration 1:1000

Mouse anti-β-catenin (for immunostaining)	Abcam	Ab305261, concentration 1:50
Human anti-NANOG-APC (for intracellular flow cytometry)	Miltenyi	130-105-079, concentration 1:11
Mouse anti-SOX2-PE (for intracellular flow cytometry)	BD Biosciences	245610, concentration 1:5
Rabbit anti-Gbx2 (for Western Blot)	Thermo Fisher	PA5-66953, concentration 1:1000
Rabbit anti-Vinculin (for Western Blot)	Cell Signaling Technology	E1E9V, concentration 1:1000
Donkey anti-goat Alexa 647	Thermo Fisher	A21447, concentration 1:500
Donkey anti-guinea pig Alexa 488	Jackson ImmunoResearch	706-545-148, concentration 1:500
Donkey anti-mouse Alexa 555	Thermo Fisher	A32773, concentration 1:500
Donkey anti-mouse Alexa 647	Thermo Fisher	A31571, concentration 1:500
Donkey anti-rabbit Alexa 555	Thermo Fisher	A-31572, concentration 1:500
Donkey anti-rabbit Alexa 647	Jackson ImmunoResearch	711-607-003, concentration 1:500
Donkey anti-goat Alexa 488	Thermo Fisher	A32814, concentration 1:500
**Chemicals, peptides, and recombinant proteins**		
mTesR Plus medium	STEMCELL Technologies	#100-0276
Penicillin/streptomycin	Thermo Fisher Scientific	15-140-122
Geltrex LDEV-Free, hESC-Qualified, Reduced Growth Factor Basement Membrane Matrix	Thermo Fisher Scientific	A1413302
Versene solution	Thermo Fisher Scientific	15040066
Accutase - Enzyme Cell Detachment Medium	Thermo Fisher Scientific	00-4555-56
TrypLE Express Enzyme (1X)	Thermo Fisher Scientific	12604013
Fetal Bovine Serum Premium Select	Atlanta Biologicals	S11550H
2-mercaptoethanol	Gibco	21985023
DMEM/F12 + GlutaMAX	Gibco	10565042
IMDM + GlutaMAX	Thermo Fisher	31980-097
F12 + GlutaMAX	Thermo Fisher	31765-092
Polyvinyl alcohol	Sigma	P8136-250G
Chemically defined lipid concentrate	Thermo Fisher	11905-031
1-thioglycerol	Sigma	M6145-100ML
Recombinant human insulin	Sigma	11376497001
Recombinant human VEGF	R&D Systems	293-VE-0500
Recombinant human FGF2	R&D Systems	233-FB-01M
Human transferrin	Sigma	10652202001
Recombinant human BMP4	R&D Systems	314-BP-050

Penicillin/streptomycin	Thermo Fisher	15070-063
Recombinant Activin	R&D Systems	338-AC-500/CF
GDC-0941	Cellagen Technology	C4321-25
Forskolin	Tocris	1099
Thiazovivin	Tocris	3845
XAV939	Tocris	3748
Ascorbic acid-2-phosphate	Sigma	49752-10G
DMH1	Tocris	4126
CHIR99201	Tocris	4423
PD0325901	Tocris	4192
Bovine serum albumin (BSA)	Sigma	A2153
Neurobasal medium	Thermo Fisher Scientific	21103049
B27	Thermo Fisher Scientific	17504044
N2	Thermo Fisher Scientific	17502001
NGS WNT-Fc	K. Christopher Garcia's laboratory	[201]
RSPO2/R-spondin 2	K. Christopher Garcia's laboratory	[201]
Recombinant human WNT3A	R&D Systems	5036-WN-010
**Critical commercial assays**		
RNeasy Plus Mini Kit	Qiagen	10010049
RNeasy Micro kit	Qiagen	74004
High-Capacity cDNA Reverse Transcription Kit	Applied Biosystems	4368814
SYBRGreen	Thomas Scientific	BIO-94050
10x Genomics Chromium single cell 3' GEM reagent kit (v3)	10x Genomics	1000075
Triton X-100	Sigma	X100-500ML
PBS, pH 7.4	Thermo Fisher	10010049
Molecular Probes DAPI (4',6 Diamidino 2 Phenylindole, Dihydrochloride)	Thermo Fisher Scientific	D1306
**Experimental models: cell lines**		
H1 hESCs	WiCell	WiCell, WA01
H7 hESCs	WiCell	WiCell, WA07
H9 hESCs	WiCell	WiCell, WA09
SUN004.1 hiPSCs	Hiromitsu Nakauchi's laboratory	[178]
SUN004.2 hiPSCs	Hiromitsu Nakauchi's laboratory	[178]

HES3 *MIXL1-GFP* hPSCs	Andrew Elefanty's, Edouard Stanley's, and Elizabeth Ng's laboratories	[181]
WTC *AAVS1-CAG-GCaMP6f* hiPSCs	Bruce Conklin's laboratory	[182]
**Software and algorithms**		
ImageJ/Fiji	[177]	https://imagej.net/software/fiji/
FastQC	[183]	http://www.bioinformatics.babraham.ac.uk/projects/fastqc/
Trim Galore	[202]	http://www.bioinformatics.babraham.ac.uk/projects/trim_galore/
Bowtie2	[192]	
MACS2	[193]	
DESeq2	[195]	
HOMER	[196]	
